# Sulfoximine and Triazine-Acid Adduct Behavior in Aqueous HPMC: A Fast and Convenient *N*‑Functionalization of Sulfoximines

**DOI:** 10.1021/acs.oprd.6c00160

**Published:** 2026-05-28

**Authors:** Dhanush Pradeep Chethikkattil, Ashish Dusunge, Michael Harmata, Wilfried M. Braje, Sachin Handa

**Affiliations:** † Department of Chemistry, 14716 University of Missouri, 601 S College Ave, Columbia, Missouri 65211, United States; ‡‡ Small Molecule Therapeutics & Platform Technologies, AbbVie Deutschland GmbH & Co. KG, Ludwigshafen 67061, Germany

**Keywords:** hydroxypropyl methylcellulose, chemistry in water, *N*-arylation, sulfoximines, reaction intermediates

## Abstract

Sulfoximines are valuable scaffolds in pharmaceuticals, yet their aqueous-phase stability and reactivity remain underexplored. We report a systematic study of sulfoximine–carboxylic acid coupling in water using hydroxypropyl methylcellulose (HPMC) as a microenvironment. In situ ^19^F NMR revealed potential key intermediates and the critical role of LiCl in their stabilization in water. The optimized protocol tolerates diverse functional groups, operates under mild conditions, and scales efficiently. These findings enable efficient sulfoximine functionalization and provide mechanistic insights.

## Graphical abstract

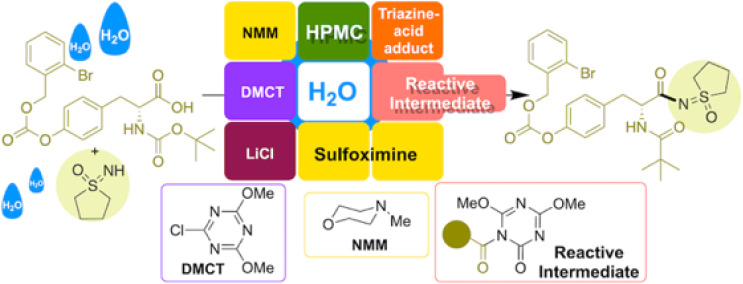

## Introduction

Over the past decade, sulfoximines have evolved from niche auxiliaries and ligands into broadly useful motifs in medicinal and process chemistry.^1–3^ This transformation is driven by their favorable physicochemical properties—enhanced polarity,^3^ tunable hydrogen bonding,^4^ stereogenic sulfur,^5^ and improved aqueous solubility^6^—combined with expanding synthetic accessibility.^7^ Their growing presence in clinical candidates (e.g., ceralasertib and BAY 1000394)^8^ and their utility as enabling groups for late-stage diversification^9^ underscore their practical value across drug discovery and scale-up (Figure 1A). Furthermore, the incorporation of sulfoximines into catalytic systems has opened new avenues for reactivity and selectivity.^7,10^

**Figure 1:**
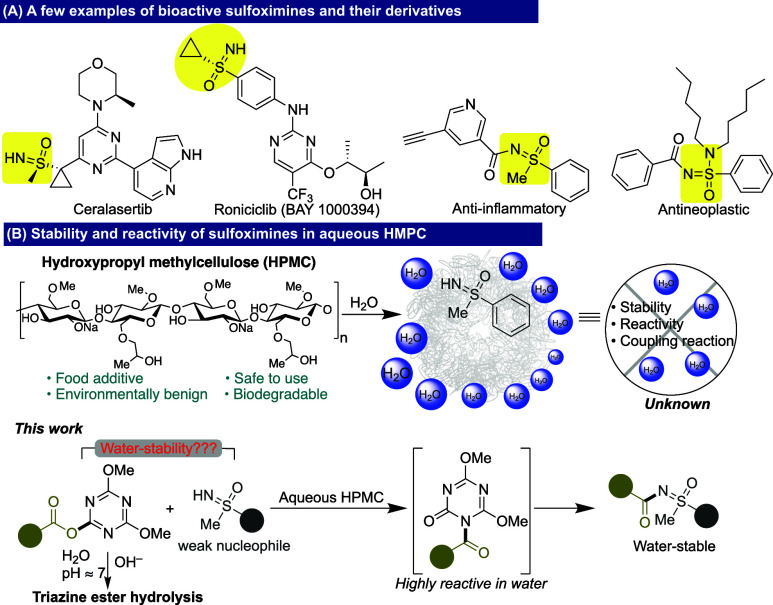
Significance of sulfoximines in medicinal chemistry and their limited reactivity in aqueous media. (A) Few examples of bioactive sulfoximines and their derivatives. (B) Stability and reactivity in aqueous HMPC.

As safer chemistry practices increasingly prioritize aqueous media for synthesis, understanding the behavior of sulfoximines in water becomes critical. In general, water has a profound effect on reaction pathways through solvation,^11^ hydrogen bonding,^4^ and pH-dependent equilibria.^10^ Yet, the stability and transformation of sulfoximines under these conditions remain poorly understood. Understanding how water influences sulfoximines persistence and conversion to *N*-acyl sulfoximines after coupling with carboxylic acid is crucial for pharmaceutical synthesis.^12,13^

Reactivity and mechanistic data of sulfoximines in aqueous media potentially enable chemists to (i) select substituents that stabilize the S­(=O)=N core against hydrolysis at a targeted pH; (ii) exploit water’s hydrogen-bond network to accelerate desired *N*-functionalizations (e.g., *N*-acylation and cross-coupling) while suppressing decomposition; and (iii) anticipate impurities or cyclized byproducts unique to acidic or basic aqueous regimes. Additionally, as sulfoximines are frequently employed as bioisosteres to modulate polarity and solubility,^12^ understanding their aqueous stability and reactivity windows could inform medicinal chemistry decisions on which analogues will endure physiological and formulation environments without off-target hydrolysis or redox liabilities. Moreover, clarifying water-mediated transformations reduces late-stage risk by aligning impurity profiles with known degradation pathways (oxidation, reduction, and C–S cleavage), which is essential for regulatory filings and environmental fate assessments.^14^

Despite growing interest, most mechanistic studies on the reactivity of sulfoximines have focused on organic solvents, leaving a significant gap in understanding the reactivity of these compounds in aqueous environments (Figure 1B).^4,8^ The conversion of sulfoximines to *N*-acyl sulfoximines via coupling with carboxylic acid—a transformation relevant to pharmaceutical intermediates and functional materials—^7^ has not been systematically explored under water-based conditions. Key questions persist: how do sulfoximines couple with carboxylic acids under aqueous media? Can the coupling reaction proceed under neutral pH (where most sensitive functional groups can be retained) conditions despite the weak nucleophilicity of sulfoximines? Addressing these gaps could help design robust and sustainable processes.

## Results and Discussion

Hydroxypropyl methylcellulose (HPMC) is a widely used pharmaceutical excipient that creates a unique aqueous microenvironment, influencing solubility, reaction kinetics, and selectivity.^15^ Leveraging this property, aqueous HPMC provides an ideal platform to study the behavior and reactivity of sulfoximines in combination with carboxylic acids under aqueous conditions. To explore this, we initiated a stability and reactivity study using the in situ-formed triazine adduct **1a**, derived from aryl carboxylic acid **1**, which features a carboxylic acid group attached to an sp^2^ carbon (also known as an sp^2^ acid). The reaction progress was monitored by ^19^F nuclear magnetic resonance (NMR) spectroscopy, as illustrated in Figure 2A. This analysis tracked (i) the hydrolysis of adduct **1a**, (ii) the formation and disappearance of intermediate **1b**, (iii) the formation of product **3** after the nucleophilic attack of sulfoximine **2**, and (iv) the subsequent degradation of **3**. The ^19^F NMR signal for acid **1** appeared at approximately −105 ppm, while adduct **1a** resonated at −103.4 ppm (w.r.t. the reference benzotrifluoride as an internal standard in a sealed capillary, δ −63.7 ppm) and product **3** at −108.4 ppm. Within 30 min, a significant amount of product **3** was detected, indicated by the emergence of the signal at −108.4 ppm, alongside a new signal for intermediate **1b** at −102.9 ppm. As the reaction progressed, the signal for **1a** disappeared within 1.5 h due to its conversion into intermediate **1b**. After 2 h, no trace of **1a** remained, while the signal intensity of **1b** decreased and **3** increased. At the same time, some Li-product adduct was observed at −107.5 ppm. By 3 h, the reaction was complete, and product **3** was isolated in 52% yield. This study confirms the transient formation of intermediate **1b** during the reaction pathway and demonstrates the sufficient stability of adduct **1a**, as well as the high stability of the sulfoximine and its coupled product **3**, enabling efficient couplings in water. Notably, in a separate control experiment, it was observed that the triazine adduct in aqueous conditions degrades significantly within 2.5 h when sulfoximine is absent (for details, see the Supporting Information on page S7).

**Figure 2:**
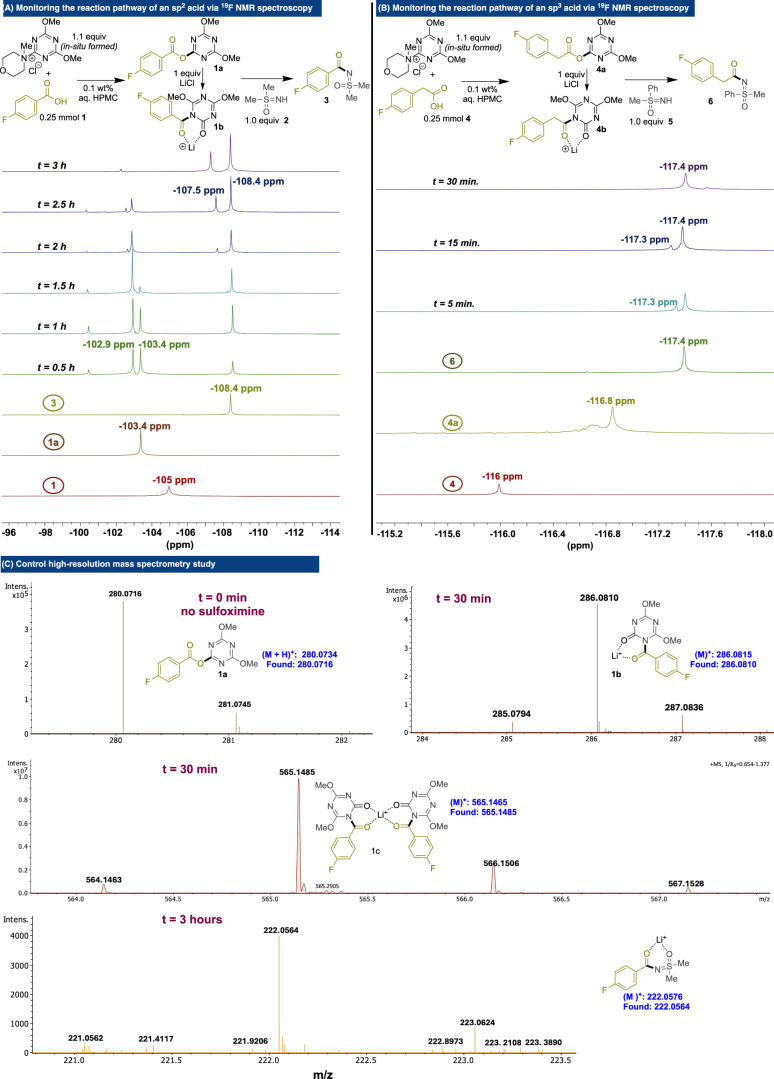
Stability analysis of triazine ester and sulfoximine and reaction pathway elucidation via ^19^F NMR spectroscopy. (A) Monitoring the reaction pathway of an sp^2^ acid via ^19^F NMR spectroscopy. (B) Monitoring the reaction pathway of an sp^3^ acid via ^19^F NMR spectroscopy. (C) Control high-resolution mass spectrometry study.

To establish generality, a ^19^F NMR control study was conducted on the coupling of an sp^3^ carboxylic acid with sulfoximine **5**. In this study, acid **4** appeared at −116 ppm, while its triazine adduct **4a** resonated at −116.8 ppm. Upon reaction with sulfoximine **5**, the product signal shifted to −117.4 ppm. Notably, the intermediate **4b** was detected only in trace amounts at −117.3 ppm, as the reaction proceeded rapidly and the desired coupling was complete within 30 min, as indicated by the product’s ^19^F signal. This study demonstrates that the triazine adduct of an sp^3^ acid undergoes a faster rearrangement and consumption for the coupling reaction than an sp^2^ acid. Furthermore, the sulfoximine-coupled product is highly stable in water, while intermediate **4a** remains sufficiently stable to participate in reaction completion. After 30 min, 78% of product **6** was isolated.

High-resolution mass spectrometry (HRMS) analysis was also conducted to investigate the formation and stability of reaction intermediate **1b** (Figure 2C). Initially, HRMS of the adduct **1a** in the reaction mixture displayed an [M + H]^+^ signal at 280.0716 Da. After 30 min, HRMS revealed additional signals corresponding to intermediates **1b** and **1c** at 286.0810 and 565.1485 Da, respectively. After 3 h, these signals were no longer detected, indicating that the final product formed from **1b** and **1c** had been completely consumed. The signal of (**3** + Li)^+^ was detected at 222.0564 Da, and no M^+^ peak was observed, indicating that the product coordinates with the lithium ion, which likely contributes to its enhanced stability in water. The detection of strong signals of **1b** and **1c**, together with supporting NMR data, strongly suggests the formation of relatively stable intermediates following the rearrangement of **1a** prior to nucleophilic attack by the sulfoximine.

Before evaluating the substrate scope, reaction parameters were further optimized for maximum efficiency. The transformation was significantly less effective in micellar media; however, 3.0 wt % PS-750-M^16,17^ provided superior performance compared to TPGS-750-M,^18^ Pluronic, Triton X, and SDS (see the Supporting Information, page S14, Table S5). The reaction proceeded in neat water, but its generality was limited. In 0.1 wt % HPMC, approximately 80% conversion was achieved within 30 min, yet the reaction was allowed to run for 3 h to ensure complete conversion. Although the process was viable at room temperature (62% yield), a temperature of 60 °C was selected to balance reaction kinetics with triazine adduct stability (Table S7, entry 3, page S15).

Following the establishment of optimized reaction conditions, the substrate scope was evaluated (Table 1, entries 3, **6**–**32**). A broad range of carboxylic acids was successfully transformed under the optimized conditions. Aryl (**3**, **16**, **19**, **30**, **31**), benzylic (**6**–**8**, **13**, **15**, **17**, **18**, **21**, **22**, **27**, **32**), alkyl (**9**, **10**, **14**), and alkenyl acids **(11**, **12)** were well tolerated, demonstrating the versatility of the protocol. Similarly, various sulfoximine coupling partners, including dimethyl sulfoximine (**3**, **27**–**32**), diphenyl sulfoximine (**7**, **8**, **10**, **12**, **13**), methylphenyl sulfoximine (**6**, **9**, **11**, **14**, **15**, **17**, **20**, **24**, **26**), and cyclic sulfoximines (**18**, **19**, **21**–**23**, **25**), participated efficiently in the coupling reaction. The method also exhibited excellent functional group tolerance. Substrates bearing fluoro (**3**, **6**, **19**), methoxy (**8**, **15**), trifluoromethyl **(14**, **16**), bromo (**9**, **10**, **21**), chloro (**13**), and carbonate (**21**) groups reacted smoothly. Notably, the reactive sp^3^-bromide functionality remained intact without undergoing undesired nucleophilic substitution (**9**, **10**). This stability is likely attributed to the mild reaction conditions and the neutral-to-mildly basic pH employed (see the Supporting Information, page S2). Likewise, terminal olefins (**11**, **12**) were unaffected by side reactions, as anticipated. Protecting groups such as benzyl carbamate (Cbz), (**20**, **23**), *tert*-butyl carbamate (Boc), (**21**, **25**, **26**), and benzyl carbonate (**21**) were well tolerated, highlighting the compatibility of the protocol with common protecting strategies. Additionally, diphenylacetic acid (**27**) and α-keto acid (**30**) underwent efficient coupling, and amino acids (**21**, **23**, **25**, **26**) were successfully incorporated. Importantly, no double-bond scrambling was observed in α-olefinic carboxylic acid (**28**), and substrates containing terminal alkynes (**31**) also furnished the desired products. Isolated yields across the scope ranged from moderate to excellent. The robustness of the method was further demonstrated by a gram-scale reaction (**15**), which delivered the product in 90% yield, comparable to the small-scale reaction. All products were stable under the aqueous conditions employed.

**Table 1:**
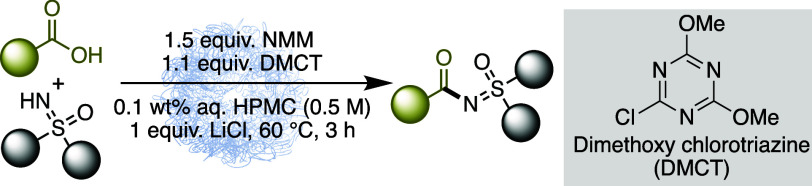

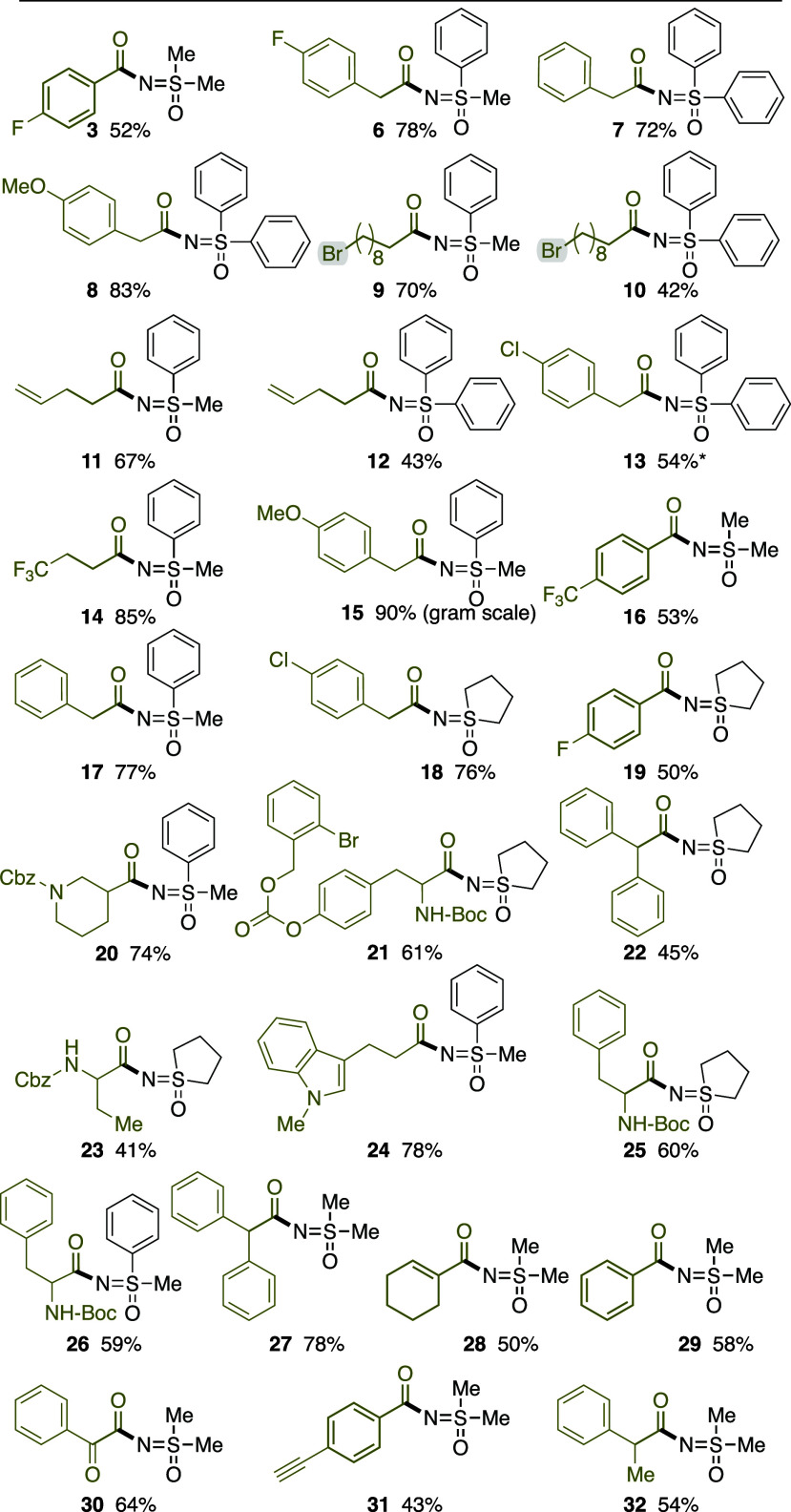
Substrate Scope^a^

a Conditions. (i) DMCT (0.55 mmol), NMM (0.75 mmol), 02 mL of THF, 5 min, rt; (ii) 0.1 mL aq. HPMC (2 mL), carboxylic acid (0.5 mmol), 5 min, rt; (iii) sulfoximine (0.5 mmol), LiCl (0.5 mmol), 60 °C. *12 h.

Control experiments revealed that, in the absence of LiCl, the reaction remained incomplete and significant decomposition of the triazine–acid adduct occurred. This observation highlights the critical role of LiCl in promoting the in situ formation and stabilization of key intermediates (e.g., **1b** and **4b**), which are crucial for achieving the desired reactivity. Based on control experiments and NMR studies, the proposed mechanism begins with the formation of adduct **a** through the reaction of NMM and DMCT (Figure 3). Adduct **a** rapidly reacts with the carboxylic acid to generate intermediate **b**. Under aqueous conditions and in the presence of LiCl, intermediate **b** undergoes a rearrangement to form intermediate **c**, which is both stable and sufficiently reactive to couple with the sulfoximine present in the reaction mixture, yielding the desired product. Importantly, both LiCl and aqueous HPMC are indispensable for this pathway. When both LiCl and aqueous HPMC or just aqueous HPMC were omitted, NMR analysis showed no evidence of intermediate **1c**, confirming their synergistic role in facilitating the reaction.

**Figure 3:**
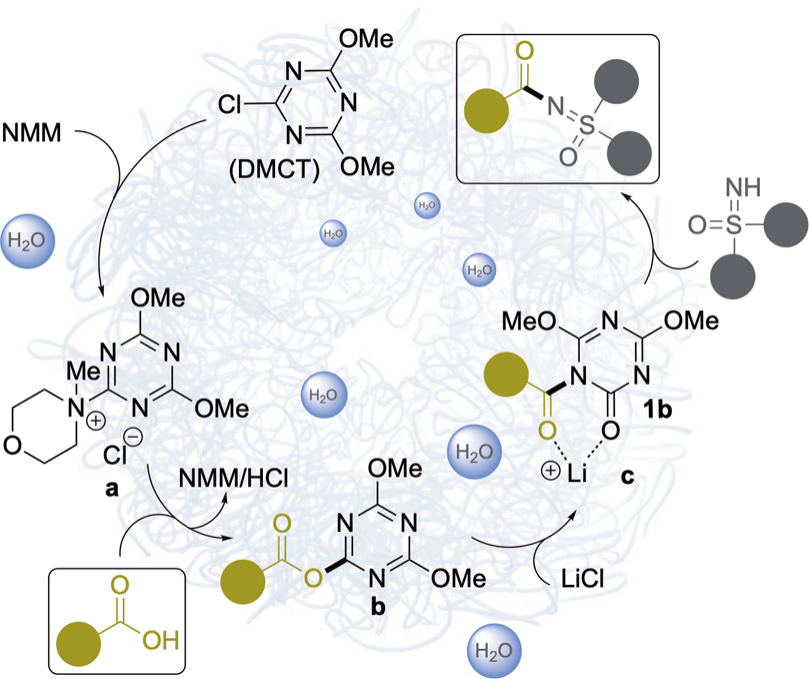
Plausible reaction mechanism.

## Conclusions

In summary, this study addresses a critical gap in understanding sulfoximine reactivity under aqueous conditions by demonstrating efficient coupling with carboxylic acids in the presence of HPMC. Mechanistic investigations reveal the stability and reactivity of triazine–acid adducts and highlight the essential role of LiCl in promoting intermediate formation and reaction completion. The optimized protocol exhibits broad functional group tolerance, compatibility with common protecting groups, and scalability, underscoring its potential for pharmaceutical and materials applications. Beyond synthetic utility, these results offer fundamental insights into sulfoximine behavior in water, paving the way for greener and more predictable processes in medicinal and process chemistry.

## Supplementary Material



## Notes

Experimental details, control experiments, analytical data, scanned NMR and HRMS data, and details of the material used (PDF)
