# Supplementary material for: Sulfoximine and Triazine-Acid Adduct Behavior in Aqueous HPMC: A Fast and Convenient N‑Functionalization of Sulfoximines
Source: Org Process Res Dev. 2026 May 28;30(6):1723–7. doi: 10.1021/acs.oprd.6c00160 (PMC13288916; doi:10.1021/acs.oprd.6c00160)

Supporting Information

Sulfoximine and Triazine-Acid Adduct Behavior in Aqueous HPMC: A Fast and Convenient *N*-Functionalization of Sulfoximines

Dhanush Pradeep Chethikkattil,[†] Ashish Dusunge,[†] Michael Harmata,[†] Wilfried M. Braje,[‡] Sachin Handa[†*]

[†]Department of Chemistry, 601 S College Ave, University of Missouri, Columbia, MO 65211, United States

[‡‡]Small Molecule Therapeutics & Platform Technologies, AbbVie Deutschland GmbH & Co. KG, Ludwigshafen, Germany

*sachin.handa@missouri.edu

Contents	Page
1. General experimental details	S1
2. General reaction procedure	S2
3. ^{19}F NMR studies of aryl carboxylic acid reactivity	S3-S4
4. Mechanistic study	S5-S9
5. Control HRMS study	S10-S12
6. Reaction optimization	S13-S15
7. Gram-scale reaction	S16
8. Analytical data	S17-S26
9. Unsuccessful examples	S27
10. References	S28
11. NMR data	S29-S52
12. HRMS data	S53-S59

1. GENERAL EXPERIMENTAL DETAILS

1.1. General reagent and material information

Analytical thin-layer chromatography (TLC) was performed on pre-coated silica gel (UV 254 indicator, thickness 200 mm), and silica gel (standard grade, 230 – 400 mesh) was purchased from Silicycle (Lot # A-000983). Visualization on TLC was achieved using UV (ultraviolet) light (254 nm), iodine on silica gel, or basic $KMnO_4$ indicator. Ethyl acetate, hexane, dichloromethane, and acetone were purchased from Fisher Scientific. Sigma-Aldrich supplied methanol and pentane. Carboxylic acids were purchased from Ambeed, Sigma-Aldrich, Oakwood, and J. T. Baker. NMR solvents were obtained from Sigma-Aldrich. *N*-methylmorpholine was purchased from Apollo Scientific. Dimethoxy chlorotriazine was purchased from Ambeed. HPMC was supplied by AbbVie.

1.2. General analytical information

All products were purified by column chromatography using silica gel (60 Å pore size, 230 - 400 mesh). GC-MS data were obtained using a Thermo Scientific Trace 1300 Gas Chromatograph coupled with a Thermo Scientific ISQ-QD Single Quadrupole Mass Spectrometer. Reported chemical shifts are referenced to residual solvent peaks. All ^{1}H and ^{13}C NMR spectra were recorded on 600 MHz (14.09 T Oxford magnet interfaced with a Bruker Avance III HD spectrometer, equipped with a commercial $^{1}H/^{13}C$ 5mm cryo-probe) and 500 MHz NMRs (11.74 T magnet operated by a Bruker Avance III HD spectrometer with a commercial broadband 5 mm probe). $CDCl_3$ was used as the solvent for NMR. The residual $CHCl_3$ for the peaks in the ^{1}H NMR (δ = 7.26 ppm) and ^{13}C NMR (δ = 77.16 ppm) spectra was used as a reference. The following abbreviations describe peak splitting patterns when appropriate: s = singlet, d = doublet, t = triplet, q = quartet, dd = doublet of doublet, m = multiplet. Coupling constants (*J*) were reported in Hertz (Hz).

Direct Infusion TOF-MS data were collected in positive-ion time-of-flight (TOF) mode from 100 to 2500 m/z and ion mobility from 0.45 to 1.6 1/K. ESI-positive data (4000kV cap voltage) were collected by infusing the compound at 3 µL/min from a 500 µL Hamilton syringe. Data were acquired over a 1-minute infusion period. The MS was calibrated with ESI-low (Agilent) just prior to acquisition; no internal recalibration was conducted. LC-TOF-MS: MS data were collected in positive-ion timsTOF-MS mode from 100 to 1300 m/z and ion mobility from 0.45 to 1.6 1/K0. ESI-positive data (1500kV cap voltage) was collected by loading 0.1 µL (~100ng) onto a Bruker Pepsep25 Ultra column (25cm x 75um x 1.5um Sapphir-C18). MS data were acquired during a short gradient LC-MS run - initial conditions were 3% B (A: 0.1% FA in water, B 99.9% ACN + 0.1%FA); rapid ramp to 80% B over 2 min, hold at 80% B for 10 min, ramp back to (2 min) and hold at initial conditions (3min). Data were summed over ~1min of peak elution. The MS was calibrated with ESI-low (Agilent) immediately prior to acquisition; no internal recalibration was performed.

2. GENERAL REACTION PROCEDURE

2.1. Synthesis of sulfoximines

Sulfoximines were synthesized using the reported procedure.[1]

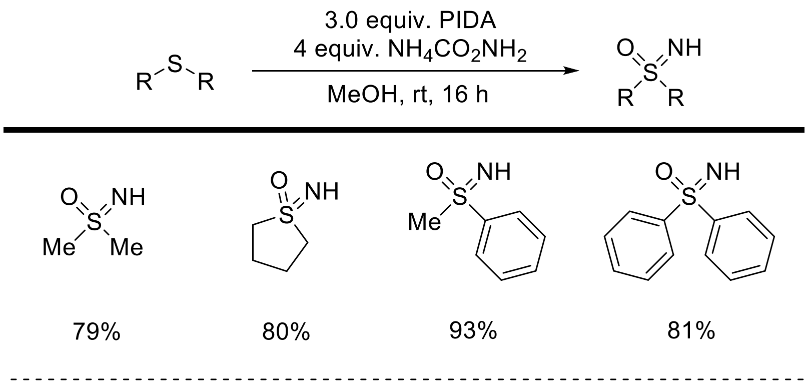

Scheme S1. Sulfoximine synthesis.

2.2. Sulfoximide synthesis

Dimethoxy chlorotriazine (DMCT, 1.1 equiv.) was added to a solution of *N*-methylmorpholine (NMM, 1.5 equiv.) in THF (0.2 mL), and the mixture was stirred for 5 min to form the adduct. 0.1 wt. % aqueous solution of hydroxypropyl methylcellulose (HPMC, 1.0 mL) was then added, followed by the addition of carboxylic acid (1.0 equiv.). The mixture was stirred for 5 min, and sulfoximine was subsequently introduced. Lithium chloride (LiCl) (1.0 equiv.) was added, and the reaction was heated at 60 °C until completion as monitored by TLC. The mixture was cooled to room temperature and extracted with ethyl acetate. The combined organic layers were dried over anhydrous Na_2SO_4, and volatiles were removed under reduced pressure. The crude product was purified by column chromatography using ethyl acetate/hexane as the eluent, affording the desired compound.

Note: Before and after the reaction, the pH of the reaction mixture was tested and was found to be *ca.* neutral. The pH was calculated using a calibrated Mettler Toledo pH meter.

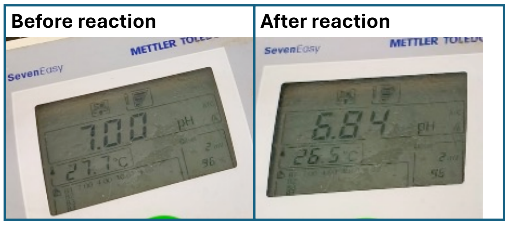

Fig. S1. pH study before and after reaction.

3. ^{19}F NMR STUDIES OF ARYL CARBOXYLIC ACID REACTIVITY

[Probing the reaction pathway of aryl carboxylic acid and the involvement of intermediates]

3.1. ^{19}F NMR study in the reaction with sp² carboxylic acid derivatives

An extensive ^{19}F NMR study was performed to monitor the reaction progress to elucidate the plausible pathway. A set of ten reactions (labeled 1–10) was conducted under identical conditions. Reaction 1 was quenched after 30 minutes, and the mixture was extracted with dichloromethane (DCM). Similarly, reactions 2–10 were quenched and extracted with DCM at intervals of 1 h, 1.5 h, 2 h, 2.5 h, and 3 h, respectively. Each sample was analyzed by ^{19}F NMR after evaporating DCM (Figure S2).

[a]Note: All reactions were performed under the standard reaction conditions.

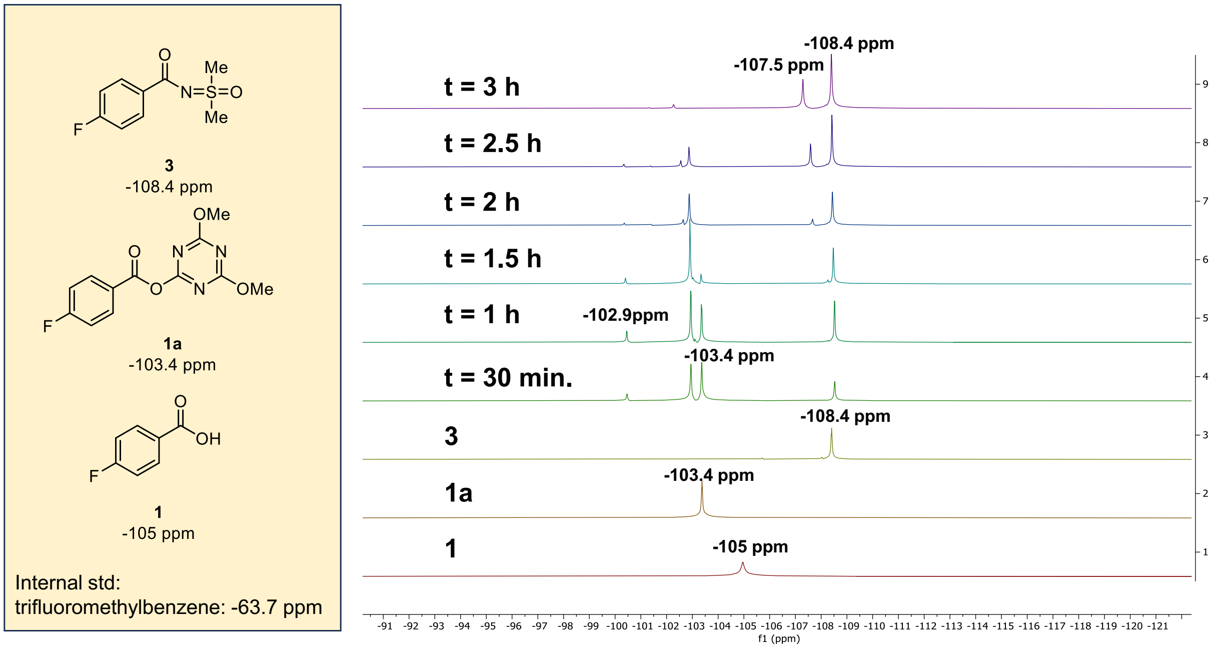

[a]Conditions: 4-Fluorobenzoic acid (0.25 mmol, 1 equiv.), dimethyl sulfoximine (0.25 mmol, 1 equiv.), DMCT (0.28 mmol, 1.1 equiv.), NMM (0.38 mmol, 1.5 equiv.), 0.1 mL THF, 0.5 mL 0.1 wt % HPMC in H_2O.

Fig. S2. Monitoring the reaction progress involving sp^2 carboxylic acid by ^{19}F NMR spectroscopy.

Note: The reaction reached completion within 3 hours, with no further conversion observed thereafter.

3.2. ^{19}F NMR study in the reaction with sp^3 carboxylic acid derivatives

Three parallel reactions were carried out under standard conditions (labeled 1–3). Reaction 1 was quenched after 5 minutes and extracted with DCM. Similarly, reactions 2 and 3 were quenched and extracted at 15 and 30 minutes, respectively.

Due to the rapid reaction rate, only traces of intermediates were detected by ^{19}F NMR (Figure S3). However, this observation highlights the efficiency of the methodology for *N*-acylation of sulfoximines. Additionally, the hydrophobic effect of HPMC prevented ester hydrolysis and ensured the formation of the desired product. Lithium chloride likely coordinates to carbonyl groups, stabilizing reactive intermediates in aqueous conditions.

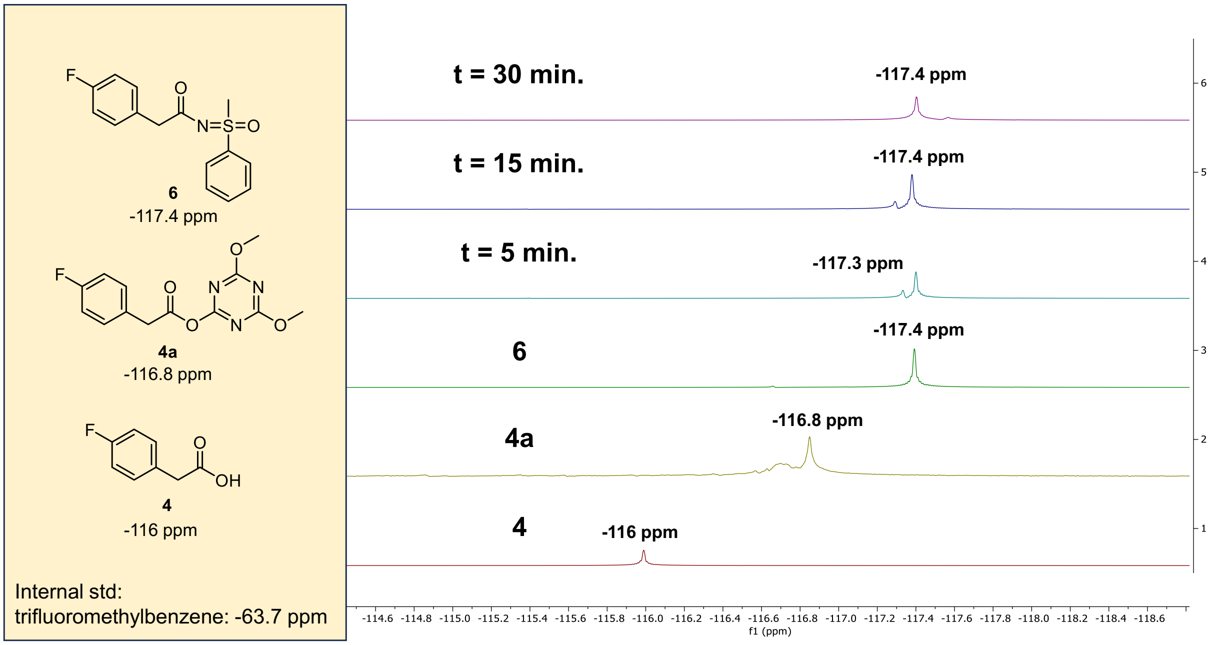

[a]**Conditions:** 4-Fluorobenzoic acid (0.25 mmol, 1.0 equiv.), dimethyl sulfoximine (0.25 mmol, 1.0 equiv.), DMCT (0.28 mmol, 1.1 equiv.), NMM (0.38 mmol, 1.5 equiv.), 0.1 mL THF, 0.5 mL 0.1 wt. % HPMC in H_2O.

Fig. S3. Monitoring the reaction progress involving sp^3 carboxylic acid by ^{19}F NMR spectroscopy.

4. MECHANISTIC STUDY

4.1. Tracing different reaction intermediates

Controlled ^{19}F NMR experiments were conducted to track the formation of byproducts (Figure S4).

Note: Reactions were performed under the standard conditions but in the absence of one of the reaction components and then analyzed by ^{19}F NMR spectroscopy.

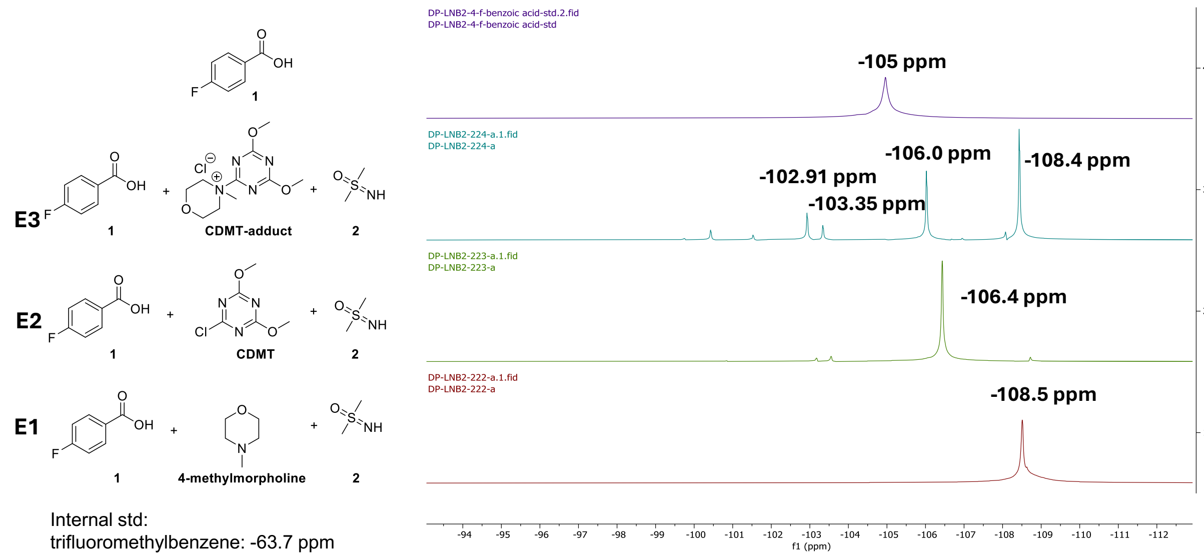

Conditions: The components (in a ratio used in the standard procedure) mentioned on the left of NMR above, 0.1 mL THF, 0.5 mL 0.1 wt. % HPMC in H_2O, 3 h.

Fig. S4. ^{19}F NMR study to track the formation of byproduct.

4.2. Influence of reaction components on the chemical shift of carboxylic acid (in the absence of sulfoximine)

Experiments E1–E4 were conducted in 0.1 wt. % aqueous HPMC to evaluate the effect of different components on the ^{19}F NMR chemical shift of 4-fluorobenzoic acid (Figure S5).

- **E1:** 4-Fluorobenzoic acid (**1**) and NMM (1:1) were mixed and vortexed for 5 minutes at room temperature. This likely forms an acid-base adduct, resulting in a ^{19}F chemical shift from –105 to –108.2 ppm.
- **E2:** 4-Fluorobenzoic acid (**1**) and DMCT (1:1) were mixed and vortexed for 5 minutes at room temperature. This may result in the formation of a hydrogen bond between the acid's OH and the *N*-atom of the triazine ring.
- **E3:** 4-Fluorobenzoic acid (**1**) and dry triazine–morpholine adduct (1:1) were mixed and vortexed for 5 minutes at room temperature.
- **E4:** 4-Fluorobenzoic acid (**1**) and 4,6-dimethoxy-1,3,5-triazin-2(1H)-one (**1a**) (1:1) were mixed, LiCl (1 equiv.) was added, and the mixture was stirred for 2.5 hours at 60 °C.

Outcome: The experiments revealed that both NMM and DMCT significantly influenced the chemical shift of 4-fluorobenzoic acid, indicating their interaction with the carboxylic acid functionality. In contrast, 4,6-dimethoxy-1,3,5-triazin-2(1H)-one (DMT-one)—a potential byproduct of the standard reaction—did not exhibit a notable effect on the chemical shift. This suggests that the observed shift changes are primarily due to the presence of nucleophilic or activating species rather than inert byproducts.

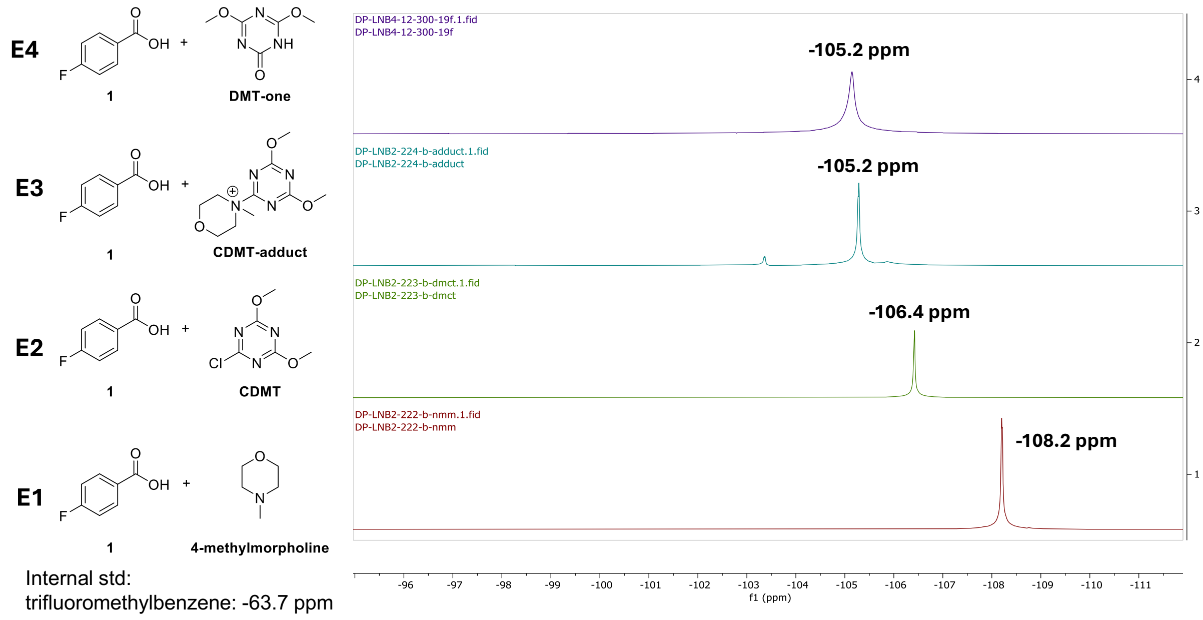

Fig. S5. ^{19}F NMR study to observe the effect of reaction components on chemical shift.

4.3. Verifying the role of sulfoximine on triazine-acid adduct degradation

A control study was conducted to track the fate of triazine-acid adduct in the presence and absence of sulfoximine (Figure S6). ^{19}F signal of triazine-acid adduct appears at –103.4 ppm.

Findings revealed that sulfoximine was not involved in the formation of the intermediates responsible for the signals at –102.9 ppm and –107.2 ppm.

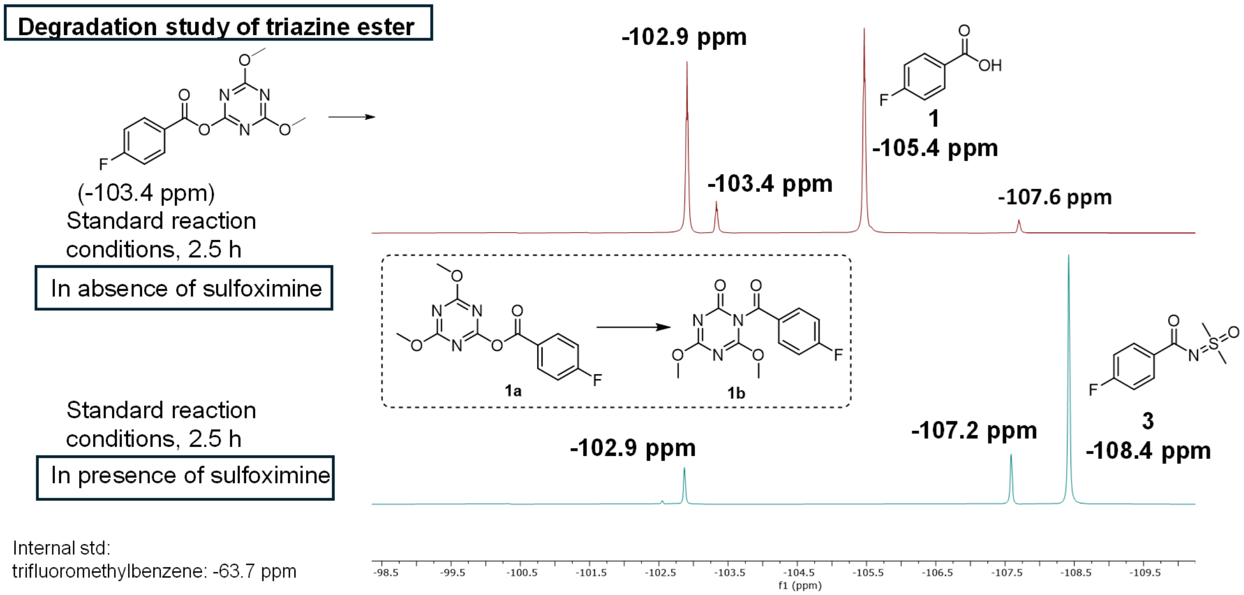

Fig. S6. ^{19}F NMR study on triazine-acid adduct degradation in the presence and absence of sulfoximine.

4.4. Effect of aqueous HPMC on the formation of reactive intermediate at –102.91 ppm

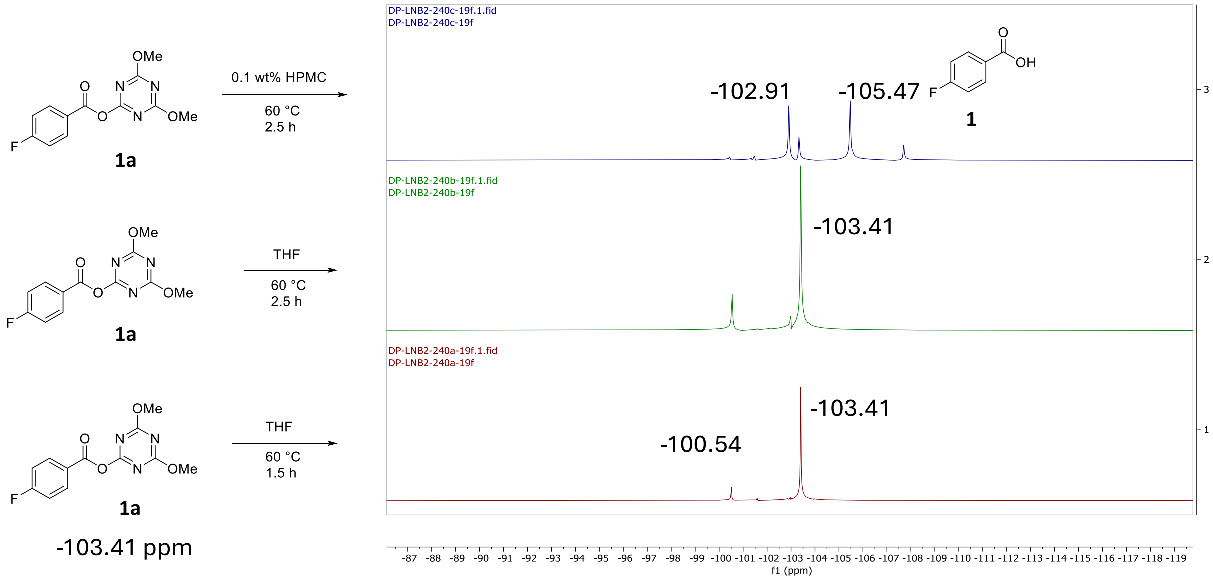

Fig. S7. ^{19}F NMR of triazine-acid adduct in THF and aqueous HPMC.

The intermediate at –102.9 ppm forms exclusively in an aqueous HPMC system; this is a rearranged intermediate similar to **1b**. In the aqueous HPMC environment, and in the absence of a sulfoximine nucleophile, the adduct undergoes hydrolysis to yield the corresponding acid. Conversely, when sulfoximine is present, the desired product is obtained, as demonstrated in previous experiments. These observations indicate that the triazine–acid adduct intermediate exhibits enhanced reactivity in aqueous HPMC, facilitating the rearrangement reaction.

4.5. Plausible reaction pathway

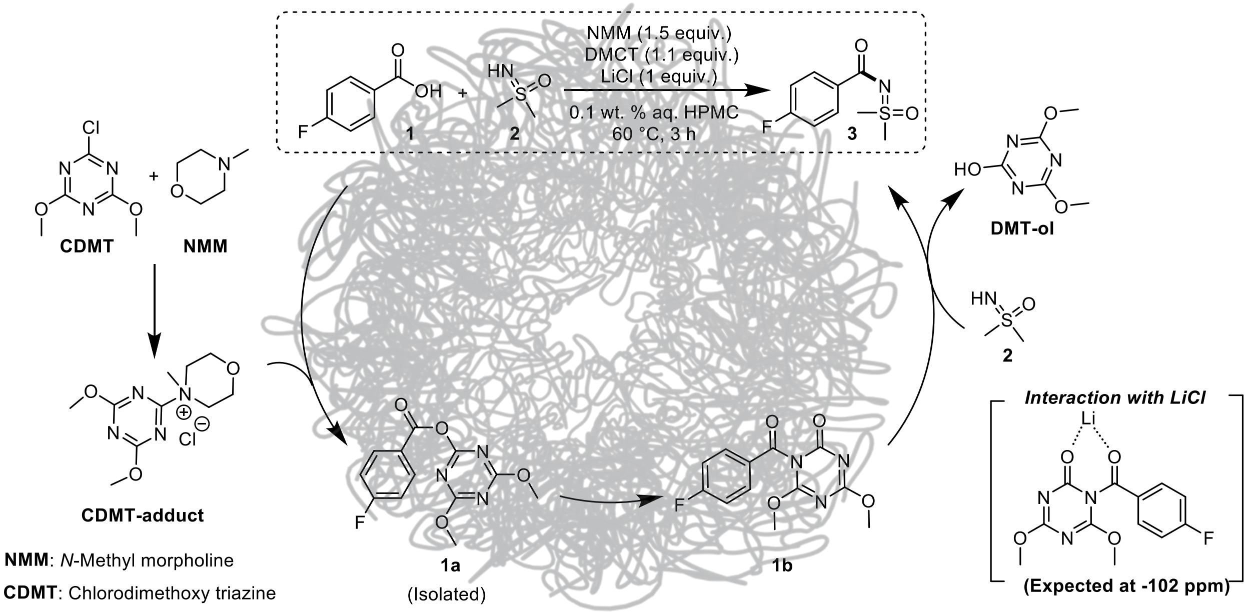

Fig. S8. A plausible reaction pathway.

Adduct formation with Lewis base: *Dimethoxy chlorotriazine* **(CDMT)** reacts with *N-methylmorpholine* **(NMM)** to form a σ-adduct **(CDMT-adduct)**.

- The morpholine nitrogen adds to the electron-deficient triazine ring (S_NAr-like), increasing the electrophilicity of the residual chloro/dimethoxy substituted triazinyl center.
- This adduct is more reactive toward acylation than DMT itself and is often formed in situ.

Carboxylic acid activation → triazine ester (1a): The carboxylate (generated by NMM) attacks the activated triazine, displacing chloride and furnishing the triazine ester (**1a**).

Rearrangement to 1b and halide stabilization: The **1a → 1b** rearrangement typically occurs an intramolecular acyl migration / tautomerization within the triazine scaffold (e.g., O→N acyl shift or site exchange on the triazine ring).

- LiCl stabilizes the rearranged species (**1b**) via tight ion pairing/coordination (Li^+ to carbonyl oxygen; Cl^- as a weakly nucleophilic counterion).
- This stabilization reduces decomposition pathways (hydrolysis, triazine ring opening) and lowers the barrier for subsequent nucleophilic acyl substitution by the sulfoximine.

Acylation of sulfoximine (2): The sulfoximine nitrogen attacks 15ae to form the amide, with the triazinyl moiety as the leaving group.

LiCl can also enhance the rate by increasing the effective acidity of the sulfoximine NH via ion pairing (improves deprotonation/formation of the nucleophilic anion) and organizing the transition state through Li^+ coordination to the carbonyl oxygen, thereby activating the acyl carbon.

5. CONTROL HRMS STUDY

5.1. High-resolution mass spectroscopy (HRMS) of the DMCT-adduct

The DMCT-adduct (**1a**) was synthesized using the general procedure described in section 2.2, page S2.

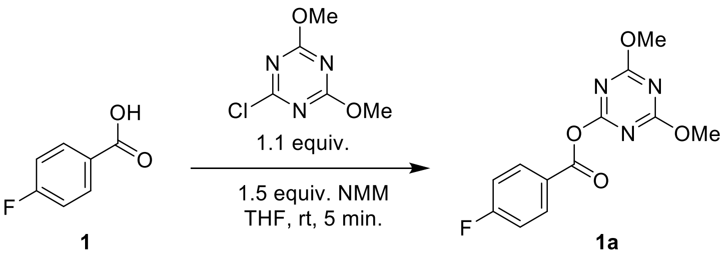

Table S1. Sample details for HRMS study 1

Entry	Constituents
1	**1** (0.25 mmol), chloro-dimethoxy triazine (DMCT, 1.1 equiv.), NMM (1.5 equiv.), THF, then stirred at room temperature for 5 min.

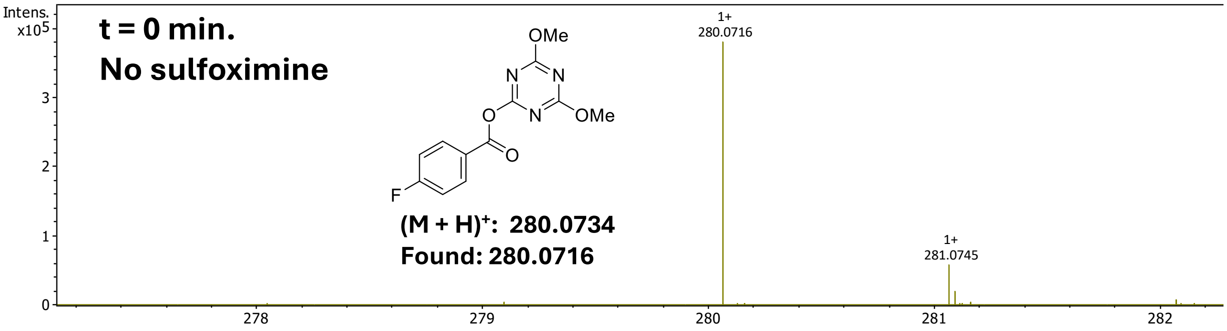

Fig. S9. HRMS spectra of **1a.**

5.2. High-resolution mass spectroscopy of the reaction mixture after 30 min.

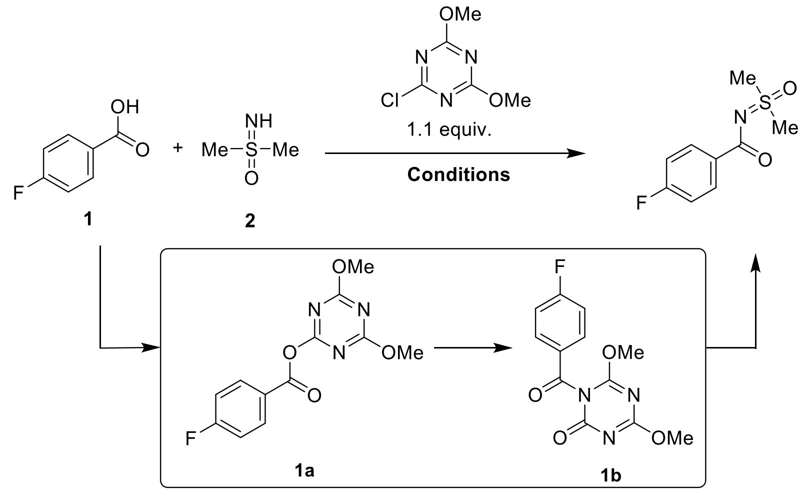

Table S2. Sample details for HRMS study 2 (reaction mixture after 30 min.)

Entry	Constituents
1	**1** (0.25 mmol), **2** (1.0 equiv.), chloro-dimethoxy triazine (DMCT, 1.1 equiv.), NMM (1.5 equiv.), LiCl (1.0 equiv.), THF (0.1 mL), 1.0 mL aq. HPMC, then stirred at 60 °C for 30 min.

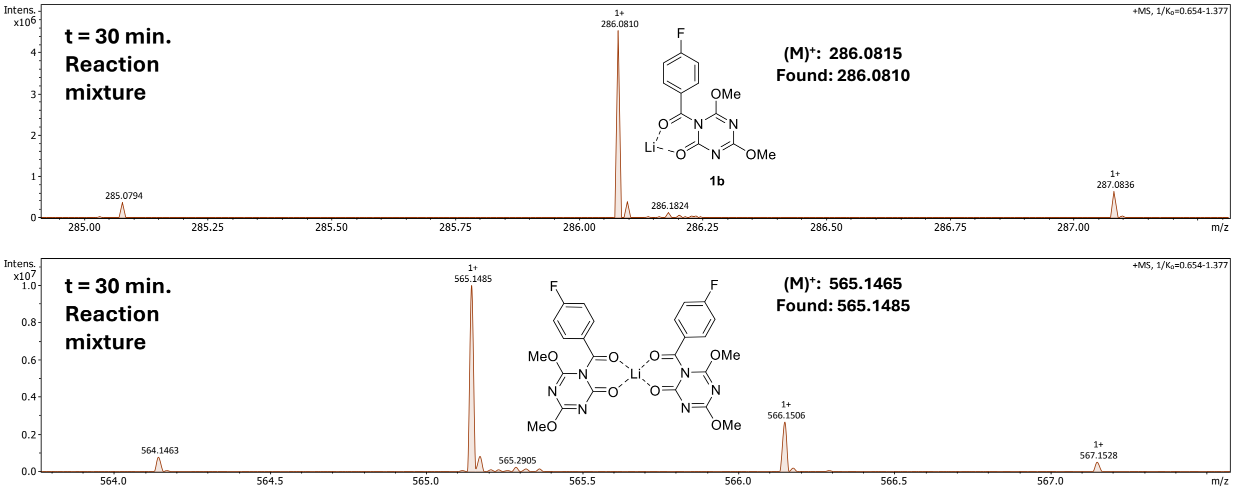

Fig. S10. HRMS spectra of plausible intermediates.

Table S3. Sample details for HRMS study 3 (reaction mixture after 3 h)

Entry	Constituents
1	**1** (0.25 mmol), **2** (1.0 equiv.), chloro-dimethoxy triazine (DMCT, 1.1 equiv.), NMM (1.5 equiv.), LiCl (1.0 equiv.), THF (0.1 mL), 1.0 mL aq. HPMC, then stirred at 60 °C for 3 h.

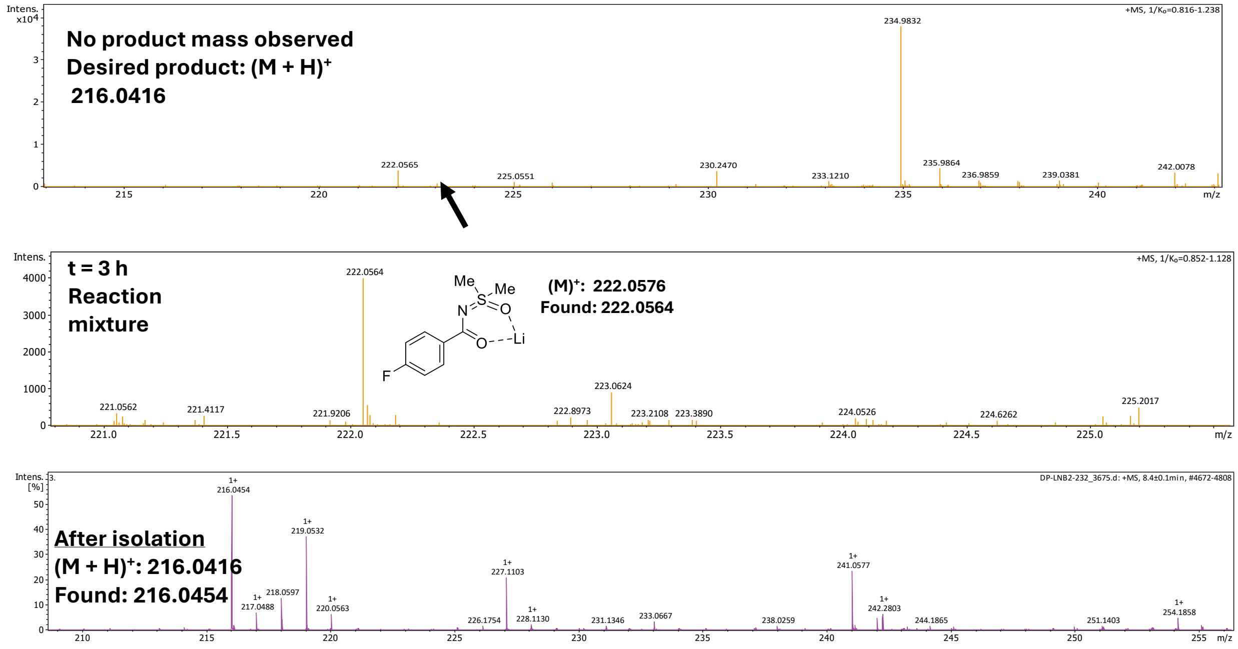

Fig. S11. HRMS spectra of (**3**)-Li adduct after 3 h, and product after isolation.

6. REACTION OPTIMIZATIONS

Note: All optimizations were performed on a 0.25 mmol scale.

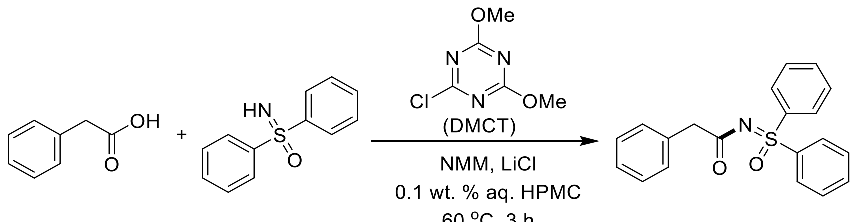

7a 7b 7

Scheme S2. General reaction scheme for the coupling of carboxylic acid and sulfoximine.

Optimizations of the coupling agent

A variety of coupling agents were evaluated for acid–amine bond formation. Due to the lower nucleophilicity of sulfoximines compared to primary amines, most conventional coupling reagents proved ineffective for acid–sulfoximine couplings. COMU exhibited good efficiency for the substrate used during initial optimization; however, its performance was inconsistent and unsuitable for several other substrates screened.

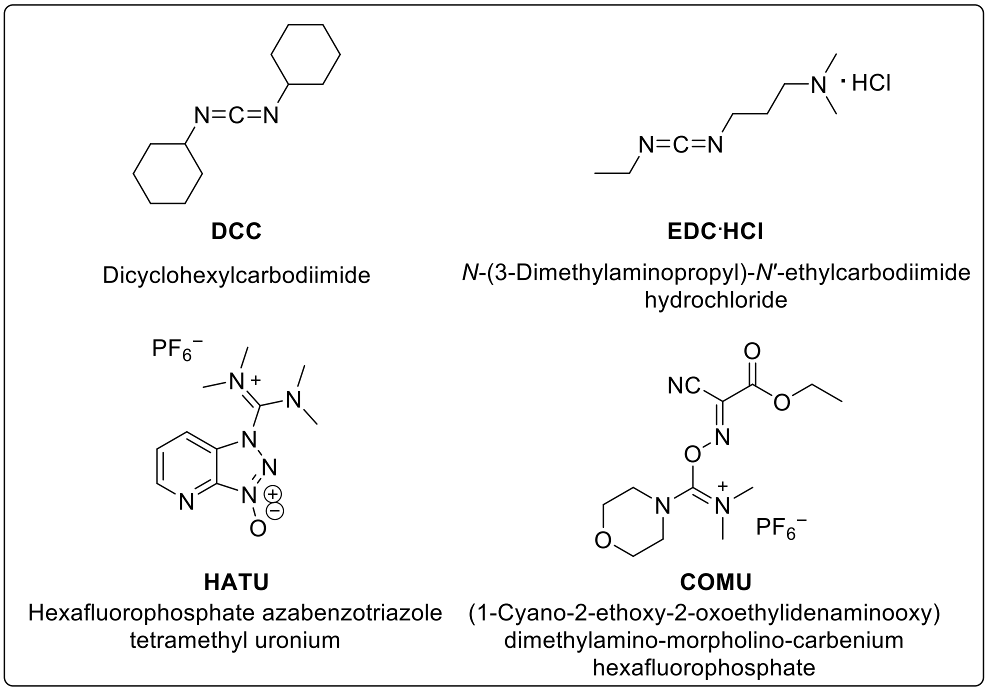

Table S4. Optimal coupling agent

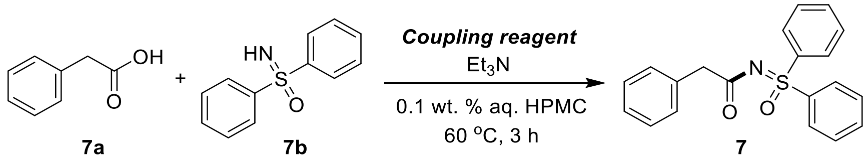

Entry	Coupling reagents	Yield (%)[a]
1	HATU	45
2	EDC•HCl	29
3	DCC	8
4	COMU	61
5[c]	DMCT•NMM	90 (72)[b]

Conditions: Phenylacetic acid (0.25 mmol, 1 equiv.), diphenyl sulfoximine (0.25 mmol, 1 equiv.), **coupling agent** (1.1 equiv.), 0.5 mL 0.1 wt. % HPMC in H_2O, 3 h. [a]Conversion based on HPLC (HPLC conditions: CHIRALPAK AD column, 40% v/v isopropanol/hexanes, 1 mL/min flow rate, at rt). [b]Conversion based on isolated yields. [c]DMCT (0.28 mmol, 1.1 equiv.), NMM (0.38 mmol, 1.5 equiv.) 0.1 mL THF, 0.5 mL 0.1 wt. % HPMC in H_2O.

Table S5. Optimal reaction medium

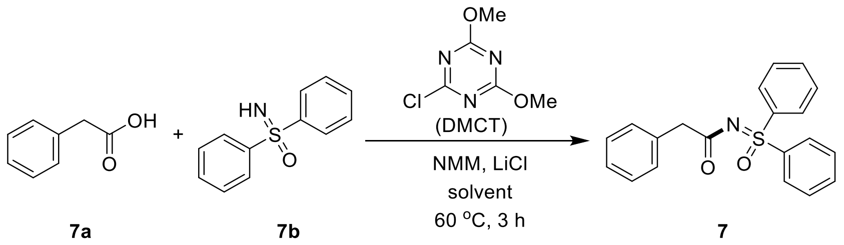

Entry	Solvents (0.5 M)	Yield (%)[a]
1	3.0 wt. % aq. PS-750-M	84
2	3.0 wt. % aq. TPGS	72
3	3.0 wt. % aq. Pluronic	55
4	3.0 wt. % aq. Triton-X	71
5	3.0 wt. % aq. SDS	76
6	3.0 wt. % aq. HPMC	90 (72)[b]

Conditions: Phenylacetic acid (0.25 mmol, 1 equiv.), diphenyl sulfoximine (0.25 mmol, 1 equiv.), DMCT (0.28 mmol, 1.1 equiv.), NMM (0.38 mmol, 1.5 equiv.), 0.1 mL THF, 0.5 mL **solvent**, 3h. [a]Conversion based on HPLC; **HPLC conditions:** CHIRALPAK AD column, 40% v/v isopropanol/hexanes, 1 mL/min flow rate, at rt. [b]Conversion based on isolated yields.

Table S6. Optimal reaction time

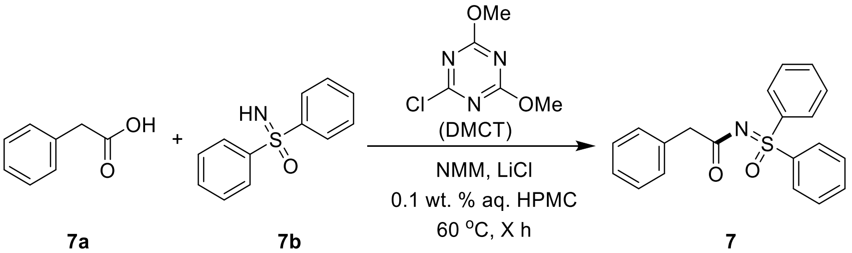

entry	time (h)	yield (%)[a]
1	0.5	80
2	1.0	81
3	3.0	90 (72)[b]

Conditions: Phenylacetic acid (0.25 mmol, 1 equiv.), diphenyl sulfoximine (0.25 mmol, 1 equiv.), DMCT (0.28 mmol, 1.1 equiv.), NMM (0.38 mmol, 1.5 equiv.), 0.1 mL THF, 0.5 mL 0.1 wt. % HPMC in H_2. [a]Conversion based on HPLC; **HPLC conditions:** CHIRALPAK AD column, 40% v/v isopropanol/hexanes, 1 mL/min flow rate, at rt. [b]Conversion based on isolated yields.

Table S7. Optimal reaction temperature

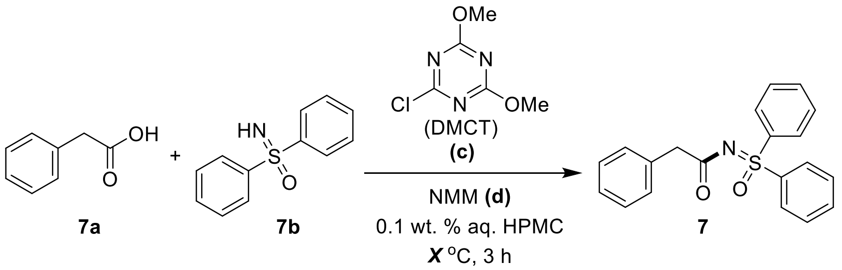

entry	temperature (°C)	yield (%)[a]
1	r.t.	62
2	45	64
3	60	90 (72)[b]

Conditions: Phenylacetic acid (0.25 mmol, 1 equiv.), diphenyl sulfoximine (0.25 mmol, 1 equiv.), DMCT (0.28 mmol, 1.1 equiv.), NMM (0.38 mmol, 1.5 equiv.), 0.1 mL THF, 0.5 mL 0.1 wt. % HPMC in H_2O, 3 h. [a]Conversion based on HPLC; **HPLC conditions:** CHIRALPAK AD column, 40% v/v isopropanol/hexanes, 1 mL/min flow rate, at rt. [b]Conversion based on isolated yields.

7. GRAM SCALE SYNTHESIS

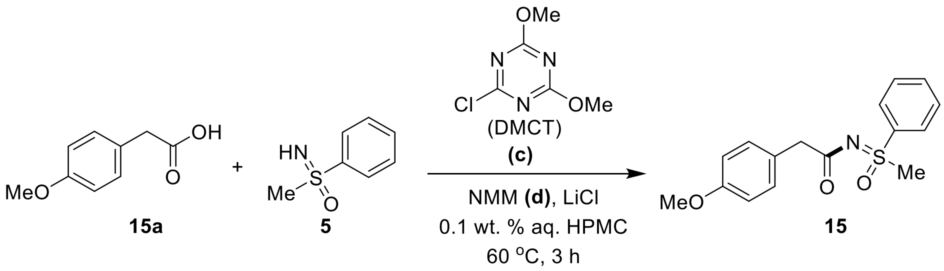

Scheme S3: Scalability test on a gram-scale reaction.

Dimethoxy chlorotriazine (1.16 g, 6.6 mmol, 1.1 equiv.) was dissolved in 2.4 mL of THF in a 50 mL round-bottom flask equipped with a PTFE-coated magnetic stir bar. *N*-methyl morpholine (913 mg, 9.0 mmol, 1.5 equiv.) was added to the solution, resulting in the formation of a white solid adduct. Subsequently, 12 mL of 0.1 wt. % aqueous HPMC was introduced into the mixture, followed by the addition of 2-(4-methoxyphenyl)acetic acid (1.0 g, 6.0 mmol, 1 equiv.), phenyl-methyl sulfoximine (933.8 mg, 6.017 mmol, 1 equiv.), and lithium chloride (256 mg, 6 mmol, 1 equiv.). The flask was sealed with a rubber septum, and the reaction mixture was stirred at 60 °C in a pre-heated oil bath for 3 hours. Reaction progress was monitored by TLC (1:1 ethyl acetate/hexane). Upon completion, the mixture was cooled to room temperature and extracted with ethyl acetate (10 mL). The organic layer was separated, dried over anhydrous sodium sulfate, and concentrated under reduced pressure to afford the crude product. Purification by column chromatography using ethyl acetate/hexane (1:1) yielded 2-(4-methoxyphenyl)-*N*-(methyl(oxo)(phenyl)- λ^6-sulfaneylidene)acetamide as the desired product, 90% yield, 1.635 g.

8. ANALYTICAL DATA

N-(Dimethyl(oxo)- λ^6-sulfaneylidene)-4-fluorobenzamide (3)[2]

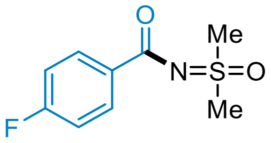

3

White solid, 56 mg (52%), R*f*: 0.4 (1:1, EtOAc/hexane).

^{1}H-NMR (600 MHz, $CDCl_3$): δ 8.12 (d, *J* = 8.4, 2H), 7.05 (d, *J* = 8.4 Hz, 2H), 3.38 (s, 6H).

2-(4-Fluorophenyl)-*N*-(methyl(oxo)(phenyl)- λ^6-sulfaneylidene)acetamide (6)

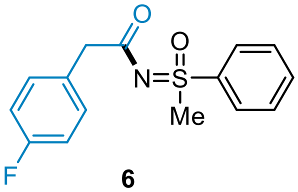

6

White solid, mp 88-90 °C, 113.2 mg (78 %), R*f*: 0.4 (3:7, EtOAc/hexane).

^{1}H NMR (500 MHz, $CDCl_3$): δ 7.83 (d, *J* = 7.9 Hz, 2H), 7.64 – 7.61 (m, 1H), 7.54 – 7.51 (m, 2H), 7.29 – 7.23 (m, 2H), 7.00 – 6.93 (m, 2H), 3.65 (s, 2H), 3.27 (s, 3H); **^{13}C NMR (126 MHz, $CDCl_3$):** δ 180.2, 162.7, 160.8, 138.5, 133.9, 131.0 (d, *J* = 7.8 Hz), 129.7, 127.01, 115.2 (d, J = 21.3 Hz), 45.7, 44.1; **^{19}F NMR (282 MHz, $CDCl_3$):** -117.4; **HRMS:** Calculated $[C_{15}H_{14}FNO_2S+H]^+$ = 292.0807**,** found m/z (ESI) = 292.0824.

N-(Oxodiphenyl- λ^6-sulfaneylidene)-2-phenylacetamide (7)[3]

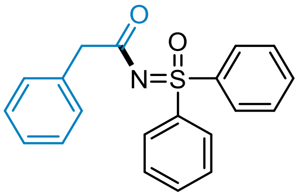

7

White solid, 120 mg (72%), R*f*: 0.4 (3:7, EtOAc/hexane).

^{1}H-NMR (600 MHz, $CDCl_3$): δ 7.76 (s, 5H), 7.55 – 7.29 (m, 10H), 3.73 (s, 2H).

2-(4-Methoxyphenyl)-*N*-(oxodiphenyl- λ^6-sulfaneylidene) acetamide (8)[1]

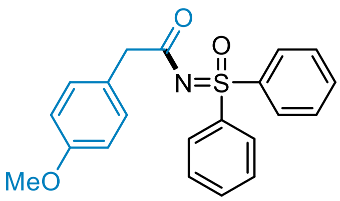

8

White solid, 152 mg (83%), R*f*: 0.4 (3:7, EtOAc/hexane).

¹H NMR (600 MHz, CDCl₃): δ 7.83 – 7.77 (m, 4H), 7.57 – 7.51 (m, 2H), 7.48 – 7.40 (m, 4H), 7.37–7.26 (m, 2H), 6.97–6.85 (m, 2H), 3.79 (s, 3H), 3.69 (s, 2H).

10-Bromo-*N*-(methyl(oxo)(phenyl)-λ⁶-sulfaneylidene)decanamide (9)

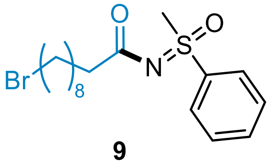

9

White solid, mp 78-80 °C; 135 mg (70%), R*f*: 0.4 (3:7, EtOAc/hexane).

¹H NMR (600 MHz, CDCl₃): δ 7.96 (d, *J* = 7.2 Hz, 2H), 7.66 (t, *J* = 7.2Hz, 1H), 7.59 (t, *J* = 7.8 Hz, 2H), 3.39 (t, *J* = 6.9 Hz, 2H), 3.33 (s, 3H), 2.38 (t, *J* = 8.2 Hz, 2H), 1.85 – 1.81 (m, 2H), 1.65 – 1.60 (m, 2H), 1.42 – 1.39 (m, 2H), 1.28 (s, 8H); **¹³C NMR (151 MHz, CDCl₃)**: δ 183.1, 139.1, 133.8, 129.7, 127.2, 44.3, 39.8, 34.2, 32.9, 29.4, 29.3, 28.8, 28.2, 25.6; **HRMS:** Calculated $[C_{17}H_{26}BrNO_2S+H]^+$ = 388.0868**,** found m/z (ESI) = 388.0887.

10-Bromo-*N*-(oxodiphenyl- λ⁶-sulfaneylidene)decanamide (10)

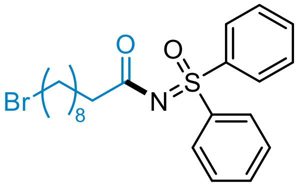

10

Colorless viscous liquid, 94 mg (42%), R*f*: 0.4 (3:7, EtOAc/hexane).

¹H-NMR (600 MHz, CDCl₃): δ 7.97 (d, *J* = 7.2 Hz, 4H), 7.57 – 7.54 (m, 2H), 7.52 – 7.48 (m, 4H), 3.41 – 3.38 (m, 2H), 2.47 (t, *J* = 7.5 Hz, 2H), 1.86 – 1.81 (m, 2H), 1.70 – 1.65 (m, 2H), 1.42 – 1.38 (m, 2H), 1.28 (s, 8H); **¹³C NMR (151 MHz, CDCl₃)**: δ 182.8, 139.8, 133.2, 129.5, 127.6, 39.9, 34.1, 32.8, 29.3, 29.2, 28.7, 28.2, 25.6; **HRMS:** Calculated $[C_{22}H_{28}BrNO_2S+H]^+$ = 450.1096, found m/z (ESI) = 450.1065.

N-(Methyl(oxo)(phenyl)- λ^6-sulfaneylidene)pent-4-enamide (11)

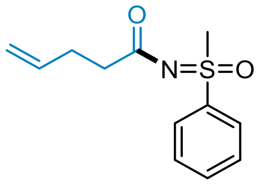

11

Colorless liquid, 79 mg (67%), R_f: 0.4 (1:1, EtOAc/hexane).
^{1}H NMR (600 MHz, CDCl$_3$): δ 7.92 (d, J = 8.2 Hz, 2H), 7.62 (t, J = 7.1 Hz, 1H), 7.58 – 7.52 (m, 2H), 5.86 – 5.77 (m, 1H), 5.00 (d, J = 17.1 Hz, 1H), 4.92 (d, J = 10.2 Hz, 1H), 3.28 (s, 3H), 2.48 – 2.43 (m, 2H), 2.38 – 2.32 (m, 2H); **^{13}C NMR (151 MHz, CDCl$_3$):** δ 182.0, 138.7, 137.6, 133.7, 129.6, 127.0, 114.8, 44.1, 38.6, 29.5; **HRMS:** Calculated $[C_{12}H_{15}NO_2S+H]^+$ = 238.0896**,** found m/z (ESI) = 238.0888.

N-(Oxodiphenyl- λ^6-sulfaneylidene)pent-4-enamide (12)

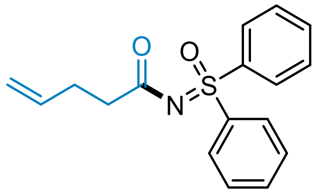

12

White solid, mp 100-105 °C, 64 mg (43%), R_f: 0.4 (3:7, EtOAc/hexane).

^{1}H NMR (600 MHz, CDCl$_3$): δ 8.00 – 7.95 (m, 4H), 7.59 – 7.54 (m, 2H), 7.54 – 7.48 (m, 4H), 5.94 – 5.84 (m, 1H), 5.07 (d, J = 17.1 Hz, 1H), 4.99 (d, J = 10.2 Hz, 1H), 2.59 (t, J = 7.5Hz, 2H), 2.46 – 2.42 (m, 2H); **^{13}C NMR (151 MHz, CDCl$_3$):** δ 181.9, 139.9, 137.8, 133.3, 129.6, 127.7, 115.0, 39.1, 29.8; **HRMS:** Calculated $[C_{17}H_{17}NO_2S+H]^+$ = 300.1053, found m/z (ESI) = 300.1052.

2-(4-Chlorophenyl)-N-(oxodiphenyl- λ^6-sulfaneylidene)acetamide (13)[1]

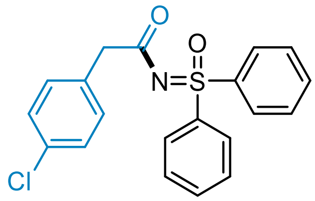

13

White solid, 100.1 mg (54%), R_f: 0.4 (3:7, EtOAc/hexane).

^{1}H-NMR (600 MHz, CDCl$_3$): δ 7.81(d, *J* = 8.0 Hz, 4H), 7.54 – 7.53 (m, 2H), 7.47 – 7.45 (m, 4H), 7.36 – 7.27 (m, 4H), 3.72 (s, 2H).

4,4,4-Trifluoro-*N*-(methyl(oxo)(phenyl)- λ^6-sulfaneylidene)butanamide (14)

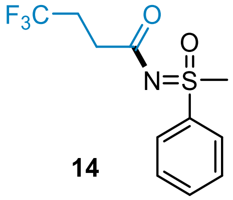

14

Colorless viscous liquid, 119 mg (85%), R*f*: 0.4 (3:7, EtOAc/hexane).

^{1}H NMR (400 MHz, CDCl$_3$): δ 7.96 (d, *J* = 7.9 Hz, 2H), 7.71 – 7.66 (m, 1H), 7.61 (t, *J* = 7.8 Hz, 2H), 3.34 (s, 3H), 2.69 – 2.63 (m, 2H), 2.49–2.40 (m, 2H); **^{13}C NMR (126 MHz, CDCl$_3$):** δ 179.4, 138.5, 134.1, 129.8, 127.1, 44.2, 32.0 (q, *J* = 2.9 Hz), 29.8 (q, *J* = 29.5 Hz); **^{19}F NMR (282 MHz, CDCl$_3$):** δ -66.64; **HRMS:** Calculated $[C_{11}H_{12}F_3NO_2S+H]^+$ =280.0619, found m/z (ESI) = 280.0634.

2-(4-Methoxyphenyl)-*N*-(methyl(oxo)(phenyl)- λ^6-sulfaneylidene)acetamide (15)

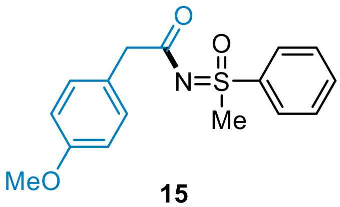

15

Yellow solid, mp 85-90 °C, crystalline solid, 1635 mg (90 %), R*f*: 0.4 (1:1, EtOAc/hexane).

^{1}H NMR (600 MHz, CDCl$_3$): δ 7.87 – 7.82 (m, 2H), 7.63 (d, *J* = 7.5 Hz, 1H), 7.56 – 7.51 (m, 2H), 7.23 (d, *J* = 8.4 Hz, 2H), 6.85 (d, *J* = 8.4 Hz, 2H), 3.79 (s, 3H), 3.64 (s, 2H), 3.29 (s, 3H); **^{13}C NMR (151 MHz, CDCl$_3$):** δ 180.9, 158.5, 138.8, 133.9, 130.6, 129.7, 128.1, 127.2, 113.9, 55.4, 45.9, 44.2; **HRMS:** Calculated $[C_{16}H_{17}NO_3S+H]^+$.=.304.1002, found m/z (ESI) = 304.0988.

***N*-(Dimethyl(oxo)- λ^6-sulfaneylidene)-4-(trifluoromethyl)benzamide (16)[4]**

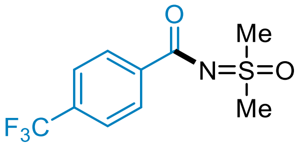

16

White solid, 70 mg (53 %), R*f*: 0.4 (1:1, EtOAc/hexane).

^{1}H-NMR (600 MHz, CDCl$_3$): δ 8.21 (d, *J* = 7.9 Hz, 2H), 7.66 (d, *J* = 7.9 Hz, 2H), 3.41 (s, 6H).

N-(Methyl(oxo)(phenyl)- λ^6-sulfaneylidene)-2-phenylacetamide (17)[3]

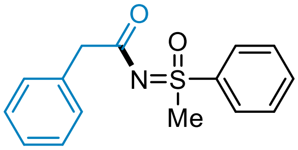

17

White solid, 120 mg (72 %), R*f*: 0.4 (1:1, EtOAc/hexane).

^{1}H-NMR (600 MHz, CDCl$_3$): δ 7.85 (d, *J* = 7.4 Hz, 2H), 7.66 (t, *J* = 7.4 Hz, 1H), 7.55 (t, *J* = 7.8 Hz, 2H), 7.38 – 7.31 (m, 4H), 7.29 – 7.25 (m, 1H), 3.72 (s, 2H), 3.31 (s, 3H).

2-(4-Chlorophenyl)-N-(1-oxidotetrahydro- λ^6-thiophen-1-ylidene)acetamide (18)

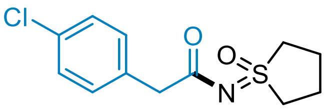

18

White solid, mp 100-102 °C, 103 mg (76 %), R*f*: 0.4 (1:1, EtOAc/hexane).

^{1}H-NMR (600 MHz, CDCl$_3$): δ 7.36 – 7.23 (m, 4H), 3.67 (s, 2H), 3.59 – 3.51 (m, 2H), 3.29 – 3.21 (m, 2H), 2.38 – 2.29 (m, 2H), 2.24 (dd, *J* = 8.5, 3.8 Hz, 2H); **^{13}C NMR (151 MHz, CDCl$_3$):** δ 180.7, 134.3, 132.5, 130.9, 128.5, 52.5, 45.2, 23.7; **HRMS:** Calculated [$C_{12}H_{14}ClNO_2S$+H]$^+$ = 272.0512**,** found m/z (ESI): 272.0523

4-Fluoro-N-(1-oxidotetrahydro- λ^6-thiophen-1-ylidene)benzamide (19) [CAS No:1865268-47-8]

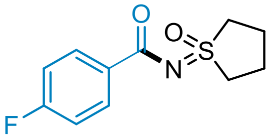

19

White solid, 60 mg (50 %), R*f*: 0.4 (1:1, EtOAc/hexane).

^{1}H-NMR (600 MHz, CDCl$_3$): δ 8.18 – 8.04 (m, 2H), 7.04 (t, *J* = 8.7 Hz, 2H), 3.68 (dt, *J* = 13.6, 6.7 Hz, 2H), 3.33 (dt, *J* = 13.6, 6.7 Hz, 2H), 2.41 – 2.32 (m, 2H), 2.32 – 2.24 (m, 2H).

Benzyl 3-((methyl(oxo)(phenyl)- λ^{6}-sulfaneylidene)carbamoyl)piperidine-1-carboxylate (20)

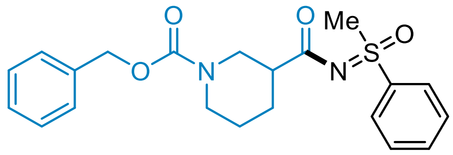

20

White waxy liquid, 68 mg (74%), R$_f$: 0.4 (1:1, EtOAc/hexane). [Note: The reaction was performed on a 0.25 mmol scale]

^{1}H NMR (600 MHz, CDCl$_3$) δ 8.07 – 7.86 (m, 2H), 7.65 (t, J = 7.4 Hz, 1H), 7.57 (q, J = 6.3 Hz, 2H), 7.38 – 7.27 (m, 5H), 5.22 – 5.02 (m, 2H), 4.39 – 4.11 (m, 1H), 4.11 – 3.82 (m, 1H), 3.28 (s, 3H), 3.06 (d, J = 40.4 Hz, 1H), 2.94 – 2.68 (m, 1H), 2.50 (s, 1H), 2.12 (d, J = 33.6 Hz, 1H), 1.82 – 1.55 (m, 2H), 1.47 (s, 1H).**^{13}C NMR (151 MHz, CDCl$_3$):** δ 182.4, 155.1, 138.5, 136.7, 133.7, 129.5, 129.5, 128.3, 127.7, 126.9, 66.8, 53.2, 46.3, 45.4, 44.6, 44.0, 24.3. **HRMS:** Calculated [$C_{21}H_{24}N_2O_4S$+H]$^+$.=.401.1530, found m/z (ESI) = 401.1516.

.

***Tert*-butyl (3-(4-((((2-bromobenzyl)oxy)carbonyl)oxy)phenyl)-1-((1-oxidotetrahydro- λ^{6}-thiophen-1-ylidene)amino)-1-oxopropan-2-yl)carbamate (21)**

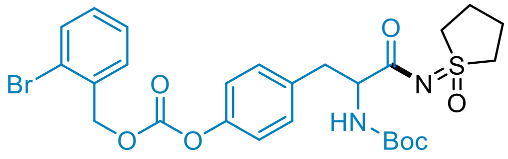

21

Waxy liquid, 180.9 mg (61%), R$_f$: 0.4 (1:1, EtOAc/hexane).

^{1}H-NMR (600 MHz, CDCl$_3$): δ 7.55 (d, J = 7.7 Hz, 1H), 7.45 (d, J = 7.5 Hz, 1H), 7.32–7.30 (m, 1H), 7.18 (d, J = 8.2 Hz, 3H), 7.05 (d, J = 8.1 Hz, 2H), 5.30 (s, 2H), 5.26 (d, J = 7.7 Hz, 1H), 4.48 (d, J = 6.7 Hz, 1H), 3.40–3.31 (m, 2H), 3.12 (d, J = 24.7 Hz, 4H), 2.21 (s, 2H), 2.08 (s, 2H), 1.39 (s, 9H). **^{13}C NMR (151 MHz, CDCl$_3$):** δ 180.0, 155.1, 153.4, 149.8, 135.1, 134.2, 132.9, 130.6, 130.2, 130.0, 127.6, 123.3, 120.6, 79.4, 69.5, 56.9, 52.6, 38.1, 28.3, 23.4; **HRMS:** Calculated [$C_{26}H_{31}BrN_2O_7S$+H]$^+$ = 595.1108**,** found m/z (ESI): 595.1078

***N*-(1-Oxidotetrahydro- λ^{6}-thiophen-1-ylidene)-2,2-diphenylacetamide (22)**

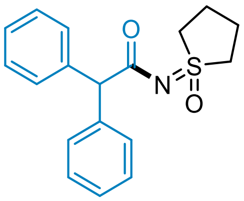

22

White solid, mp 110-115 °C, 71 mg (45%), R$_f$: 0.4 (1:1, EtOAc/hexane).

^{1}H NMR (600 MHz, CDCl$_3$): δ 7.38 – 7.33 (m, 4H), 7.30 (dd, *J* = 8.5, 6.9 Hz, 4H), 7.23 (d, *J* = 7.3 Hz, 2H), 5.09 (s, 1H), 3.58 – 3.50 (m, 2H), 3.24 – 3.16 (m, 2H), 2.33 – 2.24 (m, 2H), 2.23 – 2.14 (m, 2H). **^{13}C NMR (151 MHz, CDCl$_3$):** δ 182.2, 140.4, 129.1, 128.7, 127.1, 60.9, 52.7, 23.9; **HRMS:** Calculated [$C_{18}H_{19}NO_2S$+H]$^+$.=.314.1209, found m/z (ESI) = 314.1199.

Benzyl (1-((1-oxidotetrahydro-1l6-thiophen-1-ylidene)amino)-1-oxobutan-2-yl)carbamate (23)

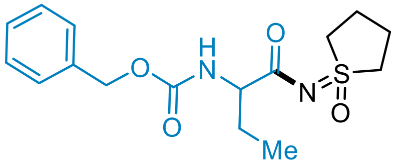

23

Waxy solid 69 mg (41 %), R$_f$: 0.4 (1:1, EtOAc/hexane).

^{1}H-NMR (600 MHz, CDCl$_3$): δ 7.31 (s, 5H), 5.54 (s, 1H), 5.05 (s, 2H), 4.24 (d, *J* = 6.9 Hz, 1H), 3.57 – 3.09 (m, 4H), 2.20 (d, *J* = 55.4 Hz, 4H), 1.91 – 1.88 (m, 1H), 1.73 – 1.69 (m, 1H), 0.88 (t, *J* = 5.9 Hz, 3H); **^{13}C NMR (151 MHz, CDCl$_3$):** δ 181.2, 156.0, 136.7, 128.5, 128.5, 128.0, 66.6, 57.8, 52.6, 26.3, 23.4, 9.4; **HRMS:** Calculated [$C_{16}H_{22}N_2O_4S$+H]$^+$ = 339.1378**,** found m/z (ESI): 339.1396.

***N*-(Methyl(oxo)(phenyl)- λ^6-sulfaneylidene)-3-(1-methyl-1H-indol-3-yl)propanamide (24)**

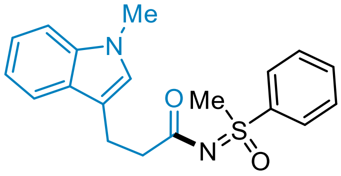

24

White solid, mp 110-112 ºC, 67 mg (78%), R$_f$: 0.4 (1:1, EtOAc/hexane). [Note: The reaction was performed on a 0.25 mmol scale].

^{1}H-NMR (600 MHz, CDCl$_3$): δ 7.82 – 7.77 (m, 2H), 7.66 – 7.60 (m, 2H), 7.51 – 7.49 (m, 2H), 7.29 (d, *J* = 8.2 Hz, 1H), 7.24 – 7.21 (m, 1H), 7.12 – 7.09 (m, 1H), 6.87 (s, 1H), 3.71 (s, 3H), 3.26 (s, 3H), 3.19 – 3.08 (m, 2H), 2.83 (t, *J* = 7.6 Hz, 2H); **^{13}C NMR (151 MHz, CDCl$_3$)** δ 182.3, 138.6, 136.8, 133.5, 129.4, 127.7, 126.9, 126.2, 121.3, 118.9, 118.5, 114.0, 108.9, 43.9, 40.0, 32.4, 21.0. **HRMS:** Calculated $[C_{19}H_{20}N_2O_2S+H]^+$ = 341.1318, found m/z (ESI) = 341.1318.

***Tert*-butyl(1-((1-oxidotetrahydro-1l6-thiophen-1-ylidene)amino)-1-oxo-3-phenylpropan-2-yl)carbamate (25)**

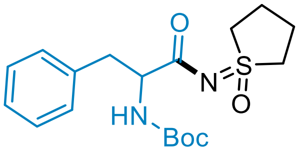

25

White solid, mp 95-98 ºC, 110 mg (60 %), R$_f$: 0.4 (1:1, EtOAc/hexane).

^{1}H-NMR (600 MHz, CDCl$_3$): δ 7.25 – 7.20 (m, 2H), 7.19 – 7.13 (m, 3H), 5.20 (m, 1H), 4.49 (s, 1H), 3.47 – 3.33 (m, 2H), 3.20 – 3.05 (m, 4H), 2.30 – 2.18 (m, 2H), 2.14 – 2.04 (m, 2H), 1.39 (s, 9H);**^{13}C NMR (151 MHz, CDCl$_3$):** δ 180.4, 155.1, 137.1, 129.6, 128.1, 126.6, 79.3, 57.0, 52.5, 38.7, 28.3, 23.5. **HRMS:** Calculated $[C_{18}H_{26}N_2O_4S\text{-Boc}]^+$ = 265.1005, found m/z (ESI) = 265.1011.

***Tert*-butyl(1-((methyl(oxo)(phenyl)-λ^6-sulfaneylidene)amino)-1-oxo-3-phenylpropan-2-yl)carbamate (26)**[5]

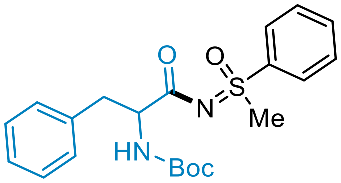

26

White solid, 119 mg (59 %), R$_f$: 0.4 (1:1, EtOAc/hexane).

^{1}H-NMR (600 MHz, CDCl$_3$): A mixture of rotomers. δ 7.88 – 7.83 (m, 2H), 7.71 – 7.66 (m, 1H), 7.61 – 7.56 (m, 2H), 7.31–7.28 (m, 2H), 7.26–7.23 (m, 3H), 5.27–5.25 (m, 1H), 4.63–4.61 (m, 1H), 3.35 (s, 2H), 3.35 (s, 1H), 3.25 – 3.12 (m, 2H), 1.44 (s, 9H).

***N*-(Dimethyl(oxo)- λ^6-sulfaneylidene)-2,2-diphenylacetamide (27)**

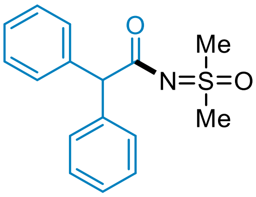

27

White solid, mp 117-120 ºC, 112.7 mg (78 %), R*f*: 0.4 (1:1, EtOAc/hexane).

^{1}H-NMR (600 MHz, CDCl$_3$): δ 7.39 (d, *J* = 7.7 Hz, 4H), 7.34 (t, *J* = 7.6 Hz, 4H), 7.28 (d, *J* = 7.3 Hz, 2H), 5.11 (s, 1H), 3.24 (s, 6H); **^{13}C NMR (151 MHz, CDCl$_3$):** δ 181.1, 140.1, 128.9, 128.5, 126.9, 61.1, 41.5; **HRMS:** Calculated [$C_{16}H_{17}NO_2S$+H]$^+$.=.288.1053, found m/z (ESI) = 288.1038.

***N*-(Dimethyl(oxo)- λ^6-sulfaneylidene)cyclohex-1-ene-1-carboxamide (28)**

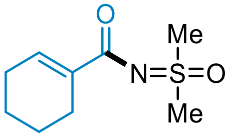

28

Waxy liquid, 50 mg (50 %), R*f*: 0.4 (1:1, EtOAc/hexane).

^{1}H NMR (600 MHz, CDCl$_3$): δ 5.81 (s, 1H), 3.26 (s, 6H), 2.29 – 2.15 (m, 2H), 2.05 – 1.89 (m, 2H), 1.85 – 1.73 (m, 2H), 1.66 – 1.51 (m, 2H); **^{13}C NMR (151 MHz, CDCl$_3$):** δ 184.1, 128.9, 126.1, 45.4, 41.7, 26.1, 24.9, 21.2; **HRMS:** Calculated [$C_9H_{15}NO_2S$+H]$^+$**.**=.202.0896, found m/z (ESI) = 202.0892.

***N*-(Dimethyl(oxo)- λ^6-sulfaneylidene)benzamide (29)[7]**

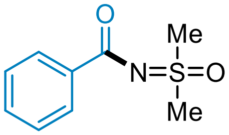

29

White solid, 57 mg (58%), R*f*: 0.4 (1:1, EtOAc/hexane).

^{1}H NMR (600 MHz, CDCl$_3$):δ 8.12 (d, *J* = 8.0, 2H), 7.52–7.49 (m, 1H), 7.42–7.39 (m, 2H), 3.39 (s, 6H).

N-(Dimethyl(oxo)- λ⁶-sulfaneylidene)-2-oxo-2-phenylacetamide (30)[6]

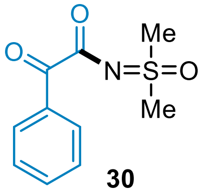

30

White solid, 72 mg (64 %), R_f: 0.4 (1:1, EtOAc/hexane).

¹H-NMR (600 MHz, $CDCl_3$): δ 8.01 (d, J = 7.9 Hz, 2H), 7.61 – 7.56 (m, 1H), 7.46 (t, J = 7.9 Hz, 2H), 3.43 (s, 6H).

N-(Dimethyl(oxo)- λ⁶-sulfaneylidene)-4-ethynylbenzamide (31)

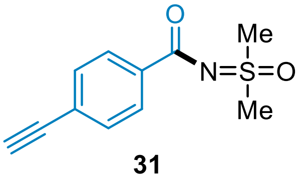

31

White solid, mp 130-133 ºC, 48 mg (43%), R_f: 0.4 (1:1, EtOAc/hexane).

¹H-NMR (600 MHz, $CDCl_3$): δ 8.07 (d, J = 8.3 Hz, 2H), 7.52 (d, J = 8.3 Hz, 2H), 3.39 (s, 6H), 3.20 (s, 1H); **¹³C NMR (151 MHz, $CDCl_3$)**: δ 173.4, 135.7, 132.0, 129.3, 126.0, 83.4, 79.6, 41.9; **HRMS:** Calculated $[C_{11}H_{11}NO_2S+H]^+$ = 222.0588, found m/z (ESI): 222.0594.

N-(Dimethyl(oxo)- λ⁶-sulfaneylidene)-2-phenylpropanamide (32)

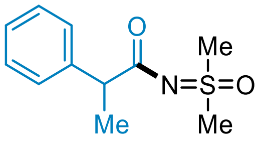

32

White solid, mp 73-75 ºC, 61 mg (54 %), R_f: 0.4 (1:1, EtOAc/hexane).

¹H-NMR (600 MHz, $CDCl_3$): δ 7.34–7.28 (m, 4H), 7.23 – 7.19 (m, 1H), 3.72 (q, J = 7.3 Hz, 1H), 3.20 (s, 3H), 3.16 (s, 3H), 1.47 (d, J = 7.4 Hz, 3H);**¹³C NMR (151 MHz, $CDCl_3$):** δ 183.4, 142.0, 128.3, 127.5, 126.6, 49.5, 41.3, 18.8. **HRMS:** Calculated $[C_{11}H_{15}NO_2S+H]^+$.=.226.0896, found m/z (ESI) = 226.0888.

9. Unsuccessful substrates

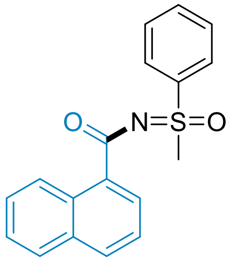

0%

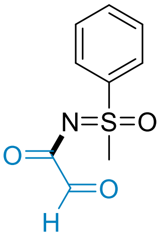

0%

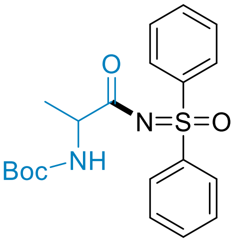

0%

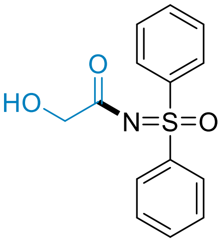

0%

Conditions: Carboxylic acid (0.25 mmol, 1 equiv.), *N*H-sulfoximine (0.25 mmol, 1 equiv.), DMCT (0.28 mmol, 1.1 equiv.), NMM (0.38 mmol, 1.5 equiv.), 0.1 mL THF, 0.5 mL 0.1 wt. % HPMC in H_2O, 3 h.

10. References

1. Li, X.; Huang, J.; Zhao, J. X.; Xu, L.; Liu, P.; Liu, J.; Wei, Y. Electrochemical strategies for N-alkylation and N-acylation of NH-sulfoximines via the decarboxylation and deoxygenation of carboxylic acids. *Org. Chem. Front.* **2025**, *12*, 3445-3453. DOI: https://doi.org/10.1039/D5QO00068H

2. Parthasarathy, K.; Bolm, C. Rhodium (III)-Catalyzed Selective ortho-Olefinations of N-Acyl and N-Aroyl Sulfoximines by C- H Bond Activation. *Chem. Eur. J.* **2014**, *20*, 4896-4900. DOI: https://doi.org/10.1002/chem.201304925

3. Bizet, V.; Buglioni, L.; Bolm, C. Light-Induced Ruthenium-Catalyzed Nitrene Transfer Reactions: A Photochemical Approach towards N-Acyl Sulfimides and Sulfoximines. *Angew. Chem. Int. Ed.* **2014**, *126*, 5745-5748. DOI: https://doi.org/10.1002/ange.201310790

4. Qi, T.; Fang, N.; Huang, W.; Chen, J.; Luo, Y.; Xia, Y. Iron (II)-catalyzed nitrene transfer reaction of sulfoxides with N-acyloxyamides. *Org. Lett.* **2022**, *24*, 5674-5678. DOI: https://doi.org/10.1021/acs.orglett.2c01990

5. Bolm, C.; Müller, D.; Dalhoff, C.; Hackenberger, C. P.; Weinhold, E. The stability of pseudopeptides bearing sulfoximines as chiral backbone modifying element towards proteinase K. *Bioorg. Med. Chem. Lett.* **2003**, *13*, 3207-3211. DOI: https://doi.org/10.1016/S0960-894X(03)00697-8

6. Zou, Y.; Peng, Z.; Dong, W.; An, D. CuI-Mediated α-Ketoacylation of Sulfoximines under Solvent-Free Conditions. *Eur. J. Org. Chem.* **2015**, *2015*, 4913-4921. DOI: https://doi.org/10.1002/ejoc.201500410

7. Kang, C.; Li, M.; Huang, W.; Wang, S.; Peng, M.; Zhao, L.; Ji, F. Electrochemical N-acylation and N-α-ketoacylation of sulfoximines via the selective decarboxylation and dehydration of α-ketoacids. *Green Chem.* **2023**, *25*, 8838-8844. DOI: https://doi.org/10.1039/D3GC02674D

11. NMR data

^{1}H NMR data of *N*-(Dimethyl(oxo)- λ^6-sulfaneylidene)-4-fluorobenzamide (3)

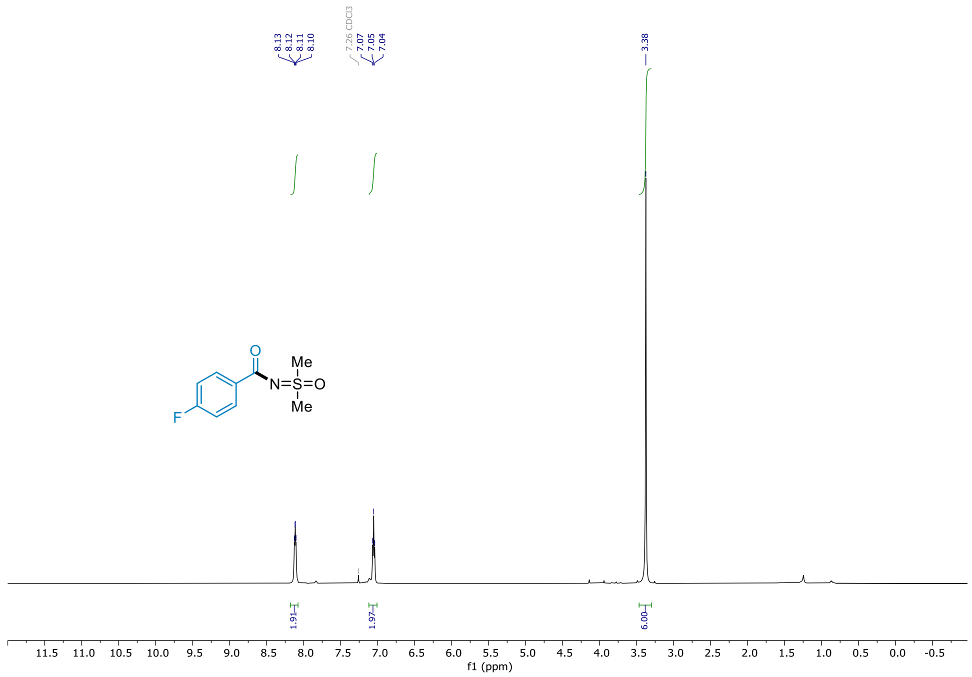

^{1}H NMR data of 2-(4-Fluorophenyl)-*N*-(methyl(oxo)(phenyl)- λ^6-sulfaneylidene)acetamide (6)

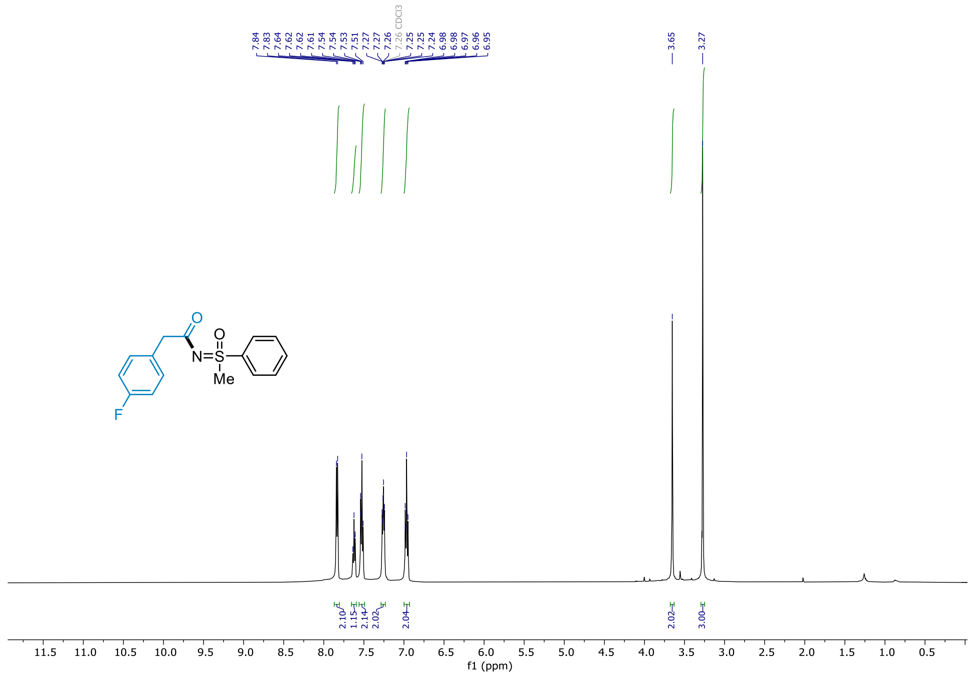

^{13}C NMR data of 2-(4-Fluorophenyl)-*N*-(methyl(oxo)(phenyl)- λ^{6}-sulfaneylidene)acetamide (6)

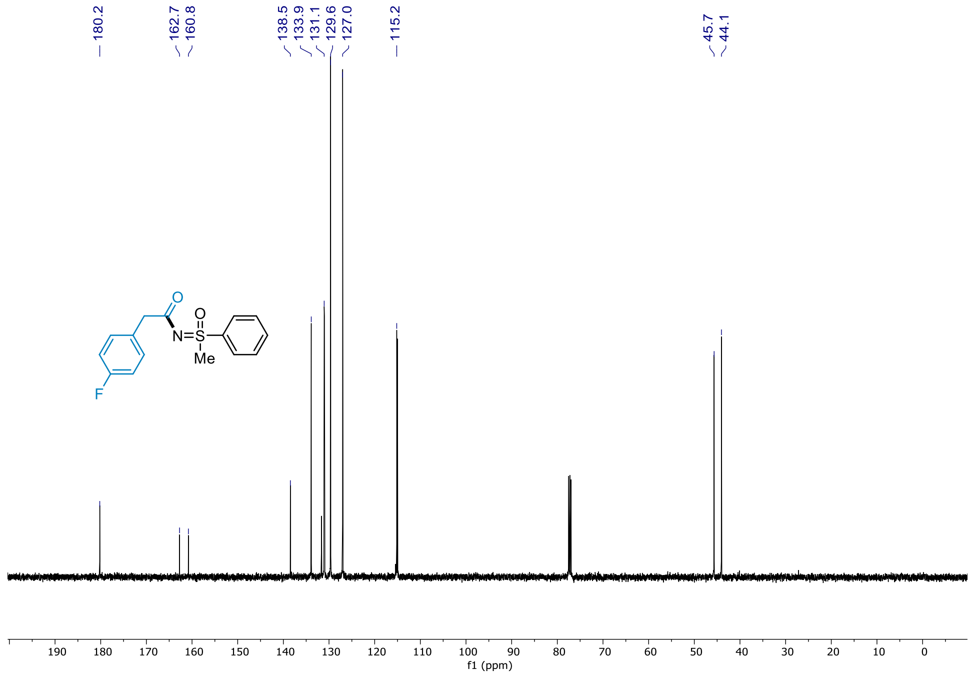

^{19}F NMR data of 2-(4-Fluorophenyl)-*N*-(methyl(oxo)(phenyl)- λ^{6}-sulfaneylidene)acetamide (6)

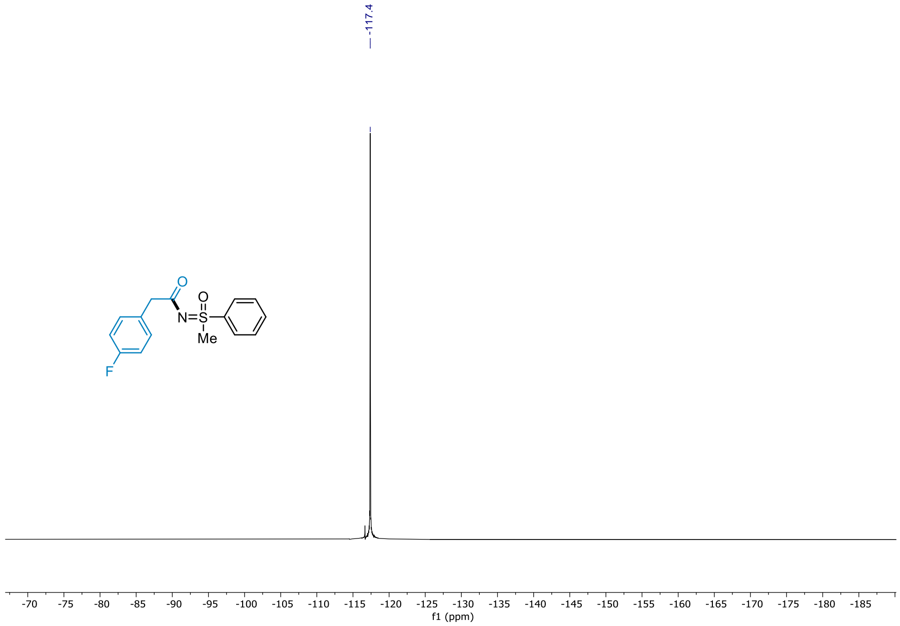

^{1}H NMR data of *N*-(Oxodiphenyl- λ^6-sulfaneylidene)-2-phenylacetamide (7)

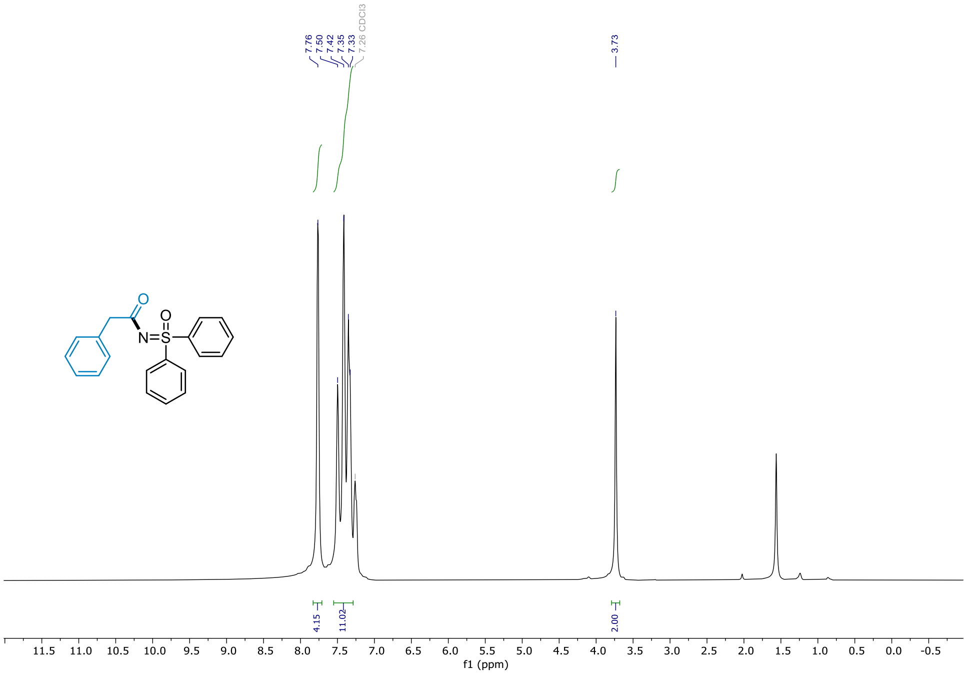

^{1}H NMR data of 2-(4-Methoxyphenyl)-*N*-(oxodiphenyl- λ^6-sulfaneylidene) acetamide (8)

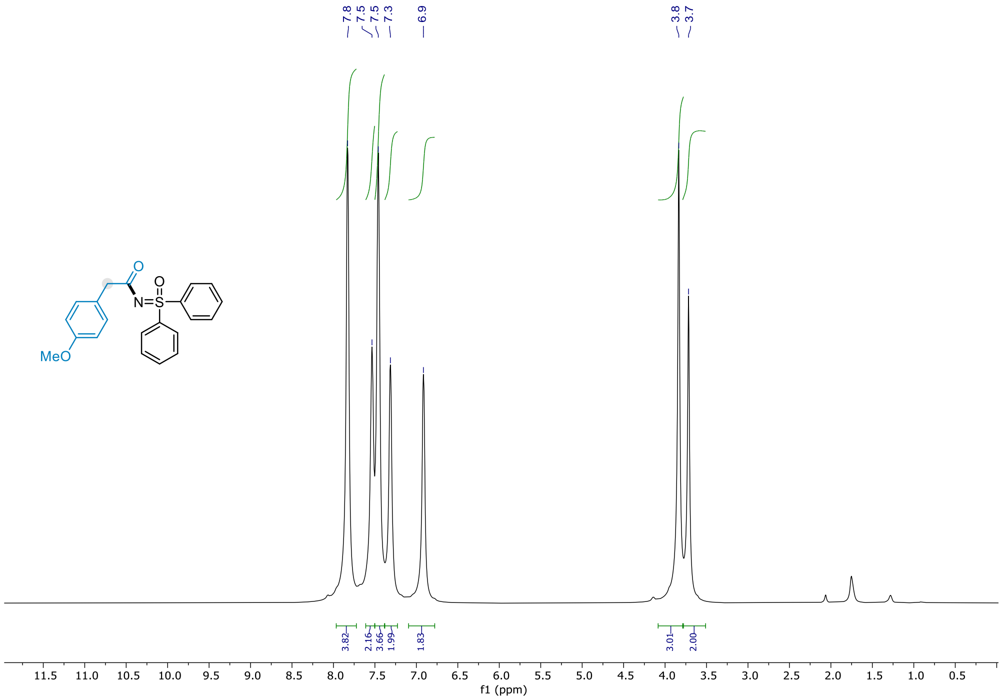

^{1}H NMR data of 10-bromo-*N*-(methyl(oxo)(phenyl)-λ^{6}-sulfaneylidene)decanamide (9)

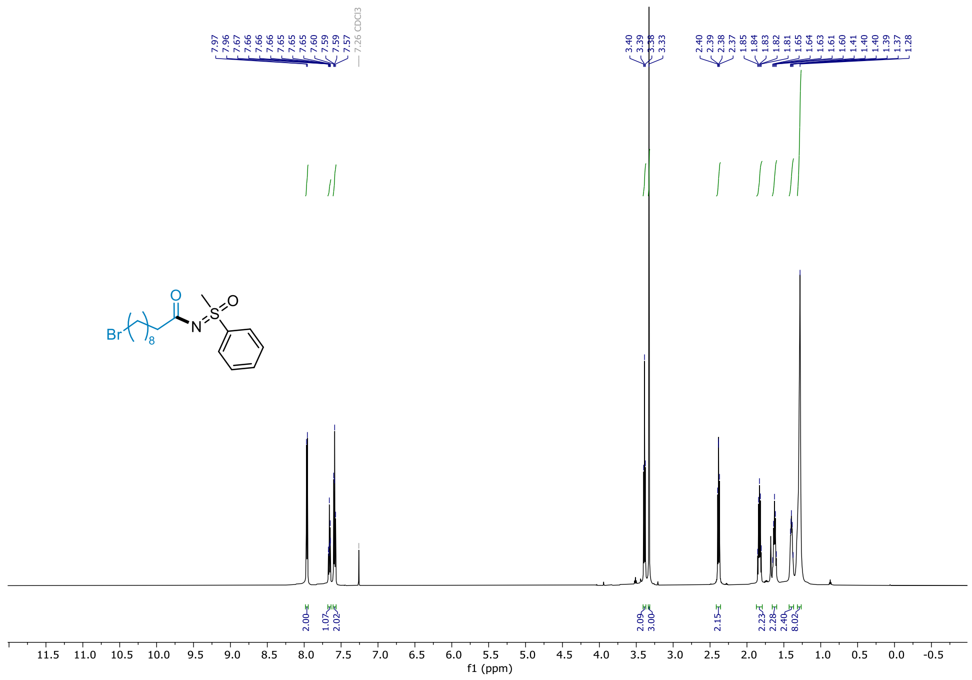

^{13}C NMR data of 10-bromo-*N*-(methyl(oxo)(phenyl)-λ^{6}-sulfaneylidene)decanamide (9)

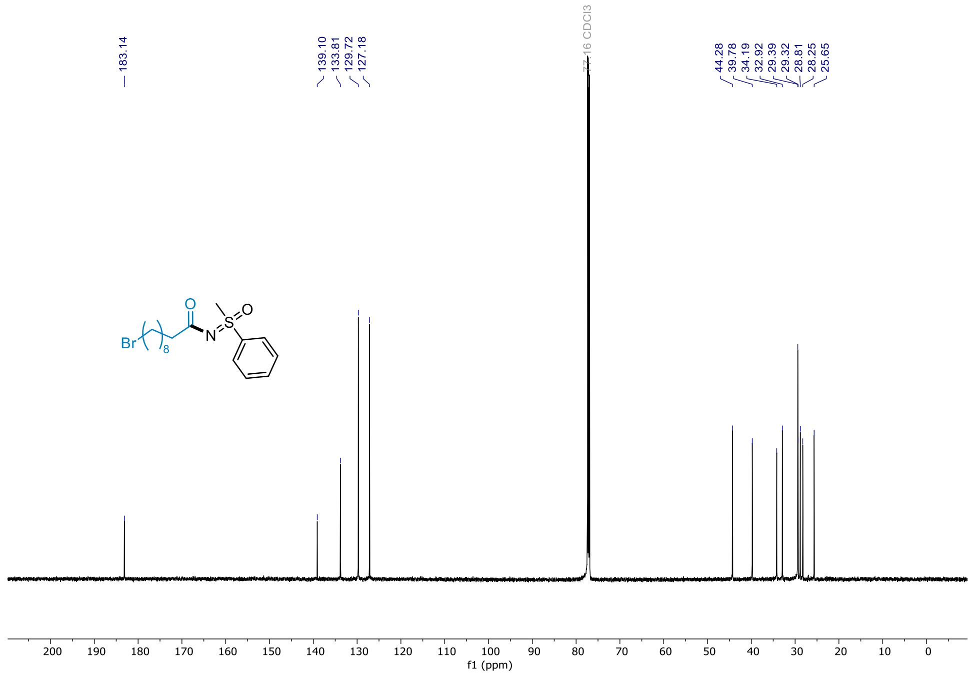

^{1}H NMR data of 10-bromo-*N*-(oxodiphenyl- λ^{6}-sulfaneylidene)decanamide (10)

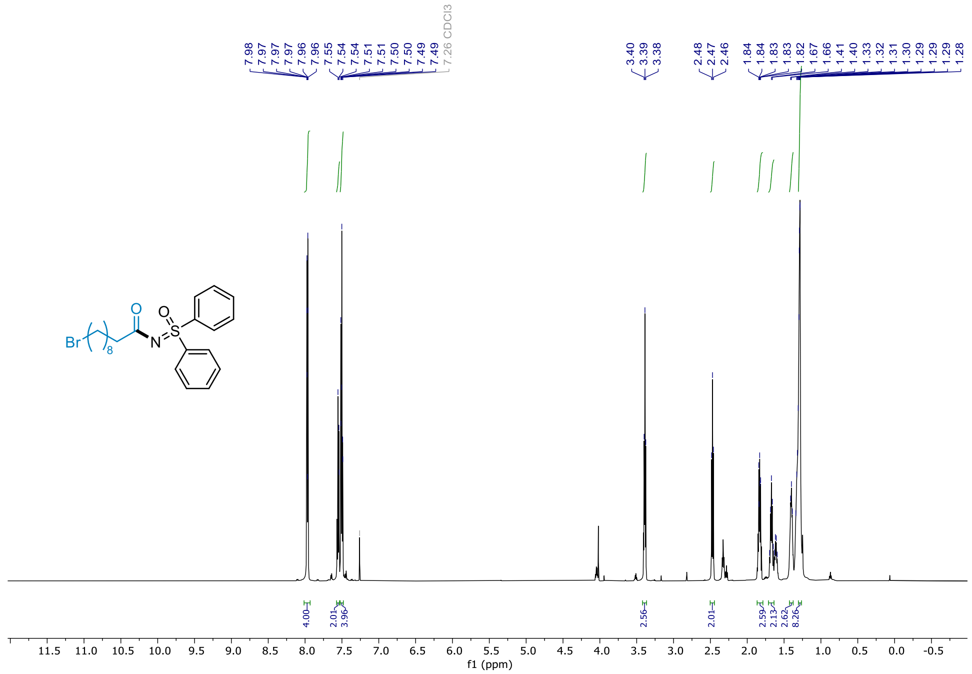

^{13}C NMR data of 10-bromo-*N*-(oxodiphenyl- λ^{6}-sulfaneylidene)decanamide (10)

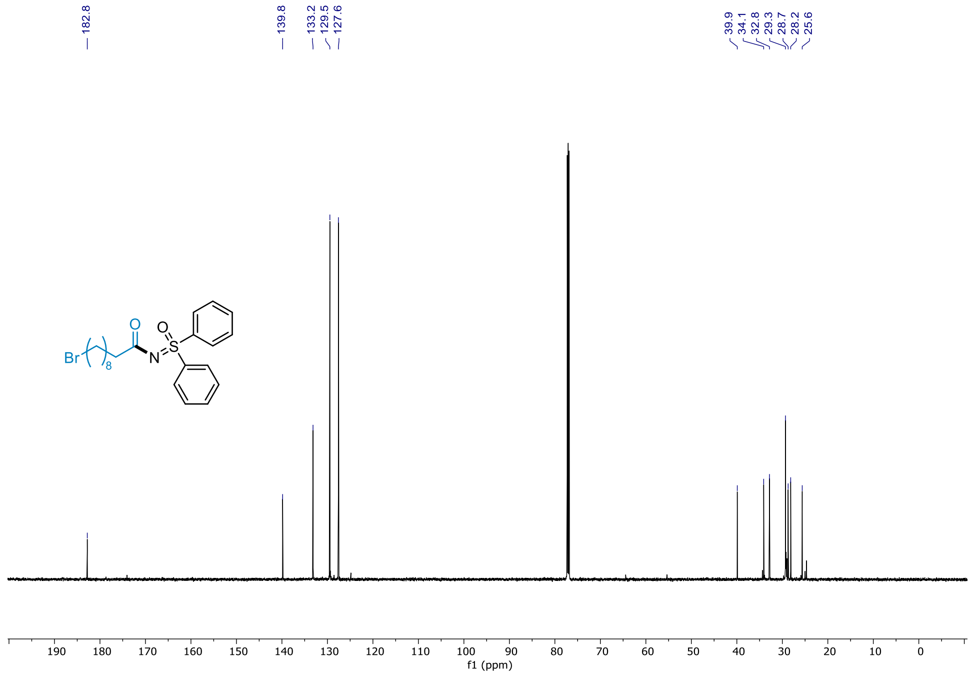

^{1}H NMR data of *N*-(methyl(oxo)(phenyl)- λ^{6}-sulfaneylidene)pent-4-enamide (11)

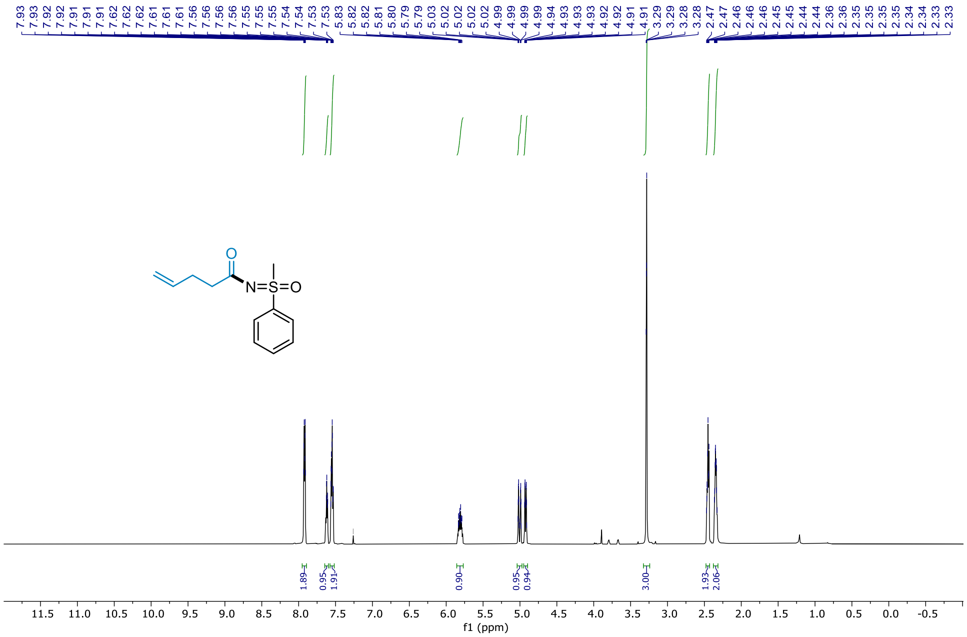

^{13}C NMR data of *N*-(methyl(oxo)(phenyl)- λ^{6}-sulfaneylidene)pent-4-enamide (11)

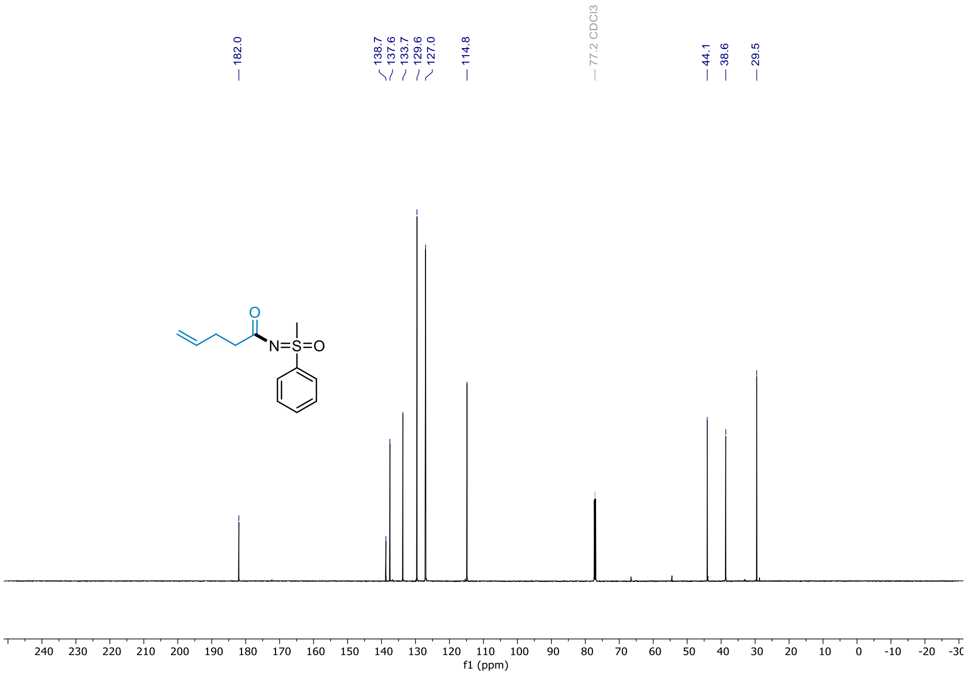

^{1}H NMR data of *N*-(oxodiphenyl- λ^{6}-sulfaneylidene)pent-4-enamide (12)

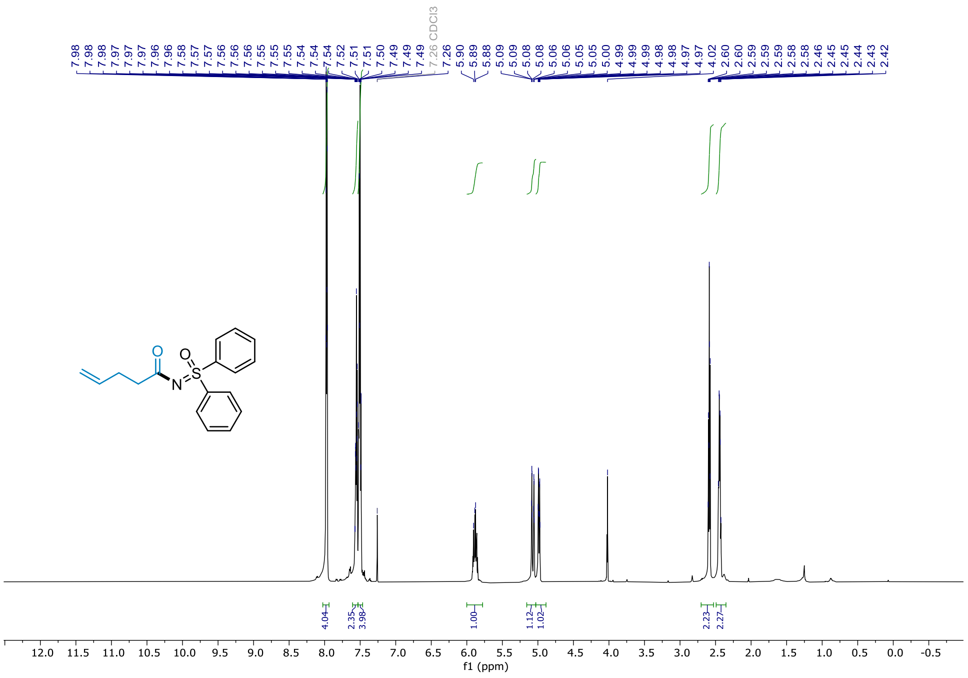

^{13}C NMR data of *N*-(oxodiphenyl- λ^{6}-sulfaneylidene)pent-4-enamide (12)

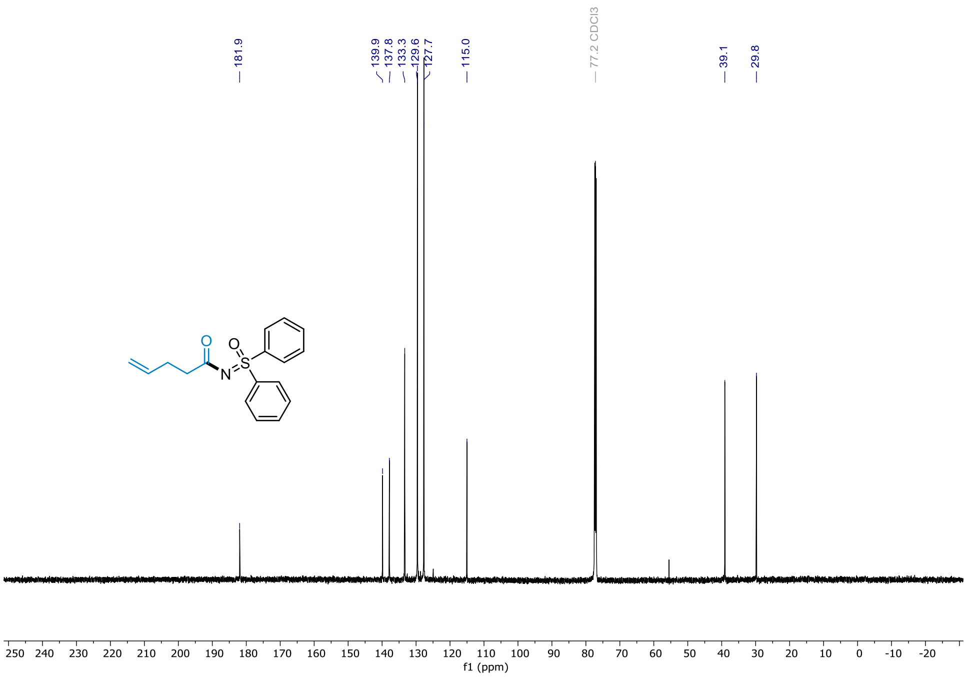

^{1}H NMR data of 2-(4-chlorophenyl)-*N*-(oxodiphenyl- λ^{6}-sulfaneylidene)acetamide (13)

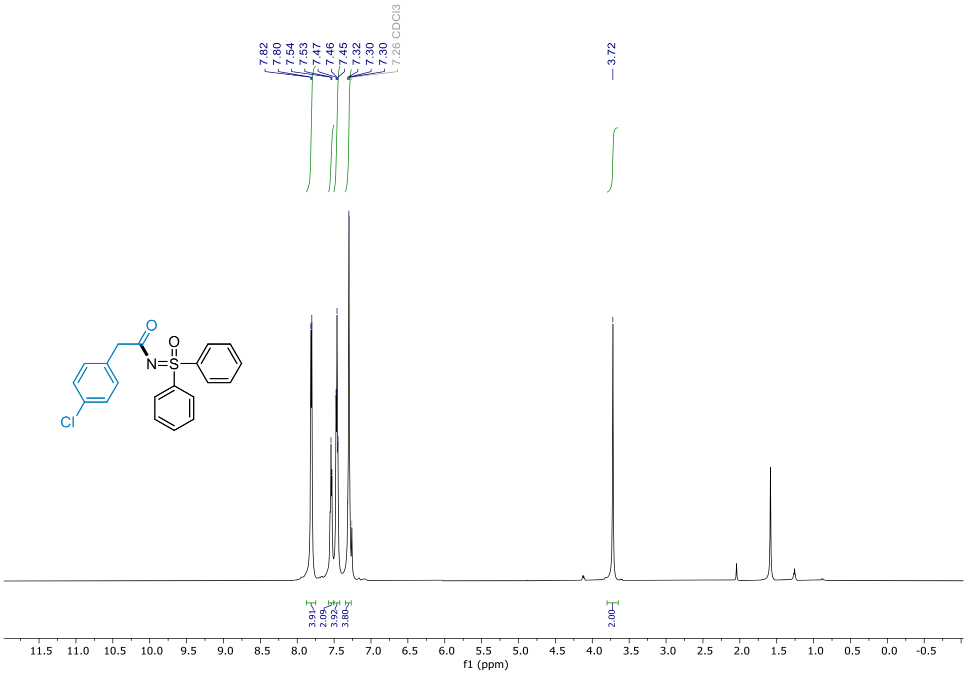

^{1}H NMR data of 4,4,4-trifluoro-*N*-(methyl(oxo)(phenyl)- λ^{6}-sulfaneylidene)butanamide (14)

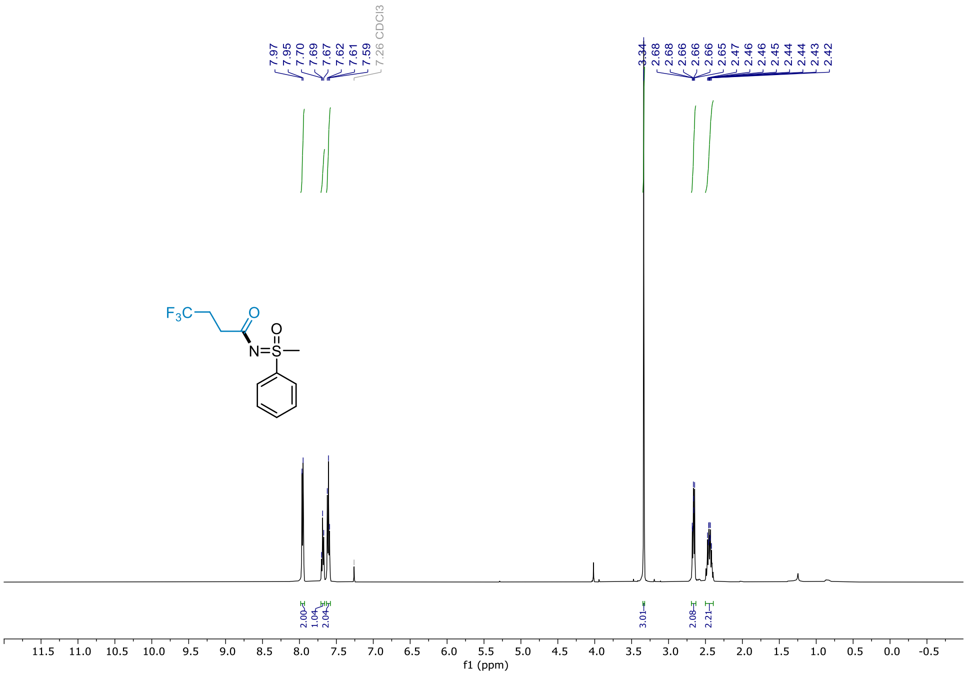

^{13}C NMR data of 4,4,4-trifluoro-*N*-(methyl(oxo)(phenyl)- λ[6]-sulfaneylidene)butanamide (14)

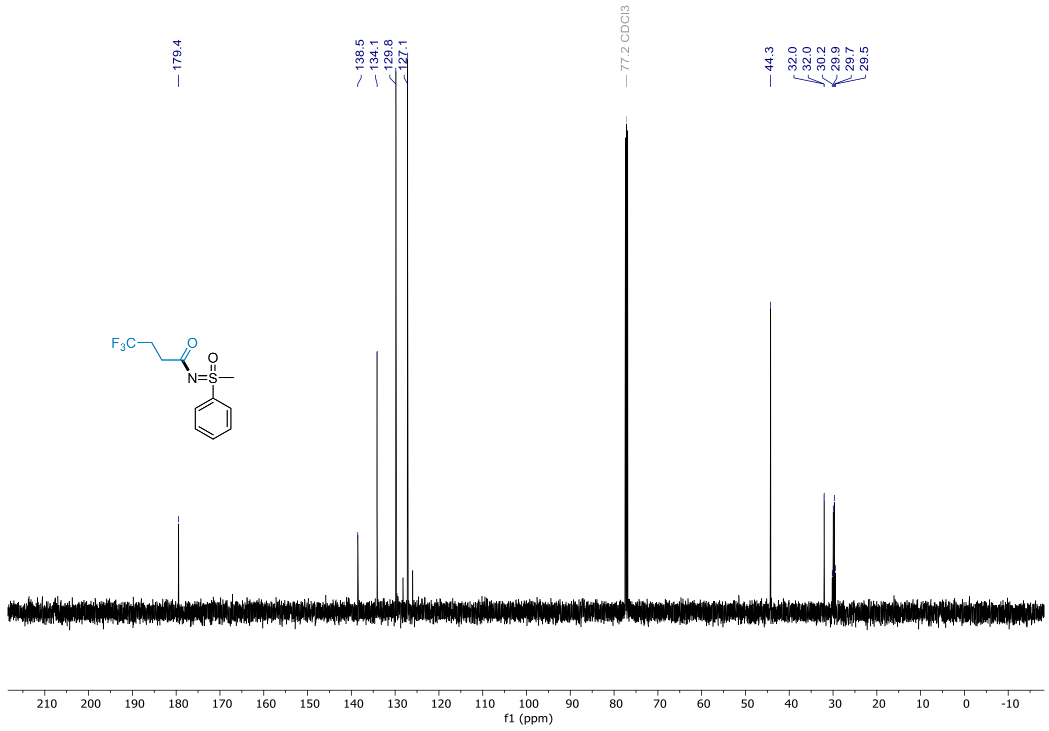

^{19}F NMR data of 4,4,4-trifluoro-*N*-(methyl(oxo)(phenyl)- λ[6]-sulfaneylidene)butanamide (14)

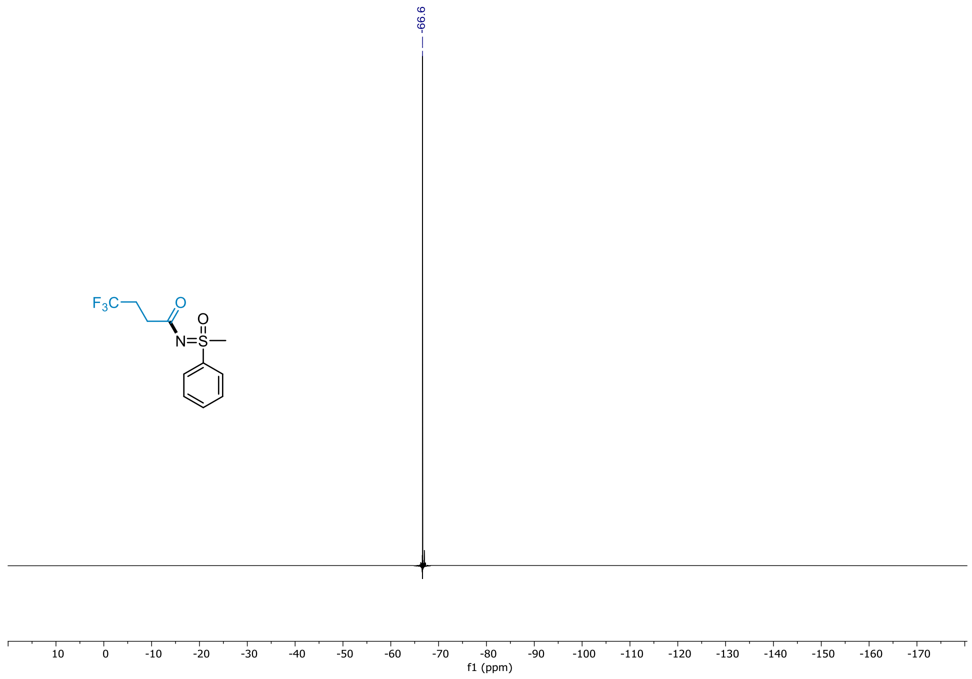

^{1}H NMR data of 2-(4-methoxyphenyl)-*N*-(methyl(oxo)(phenyl)- λ^{6}-sulfaneylidene)acetamide (15)

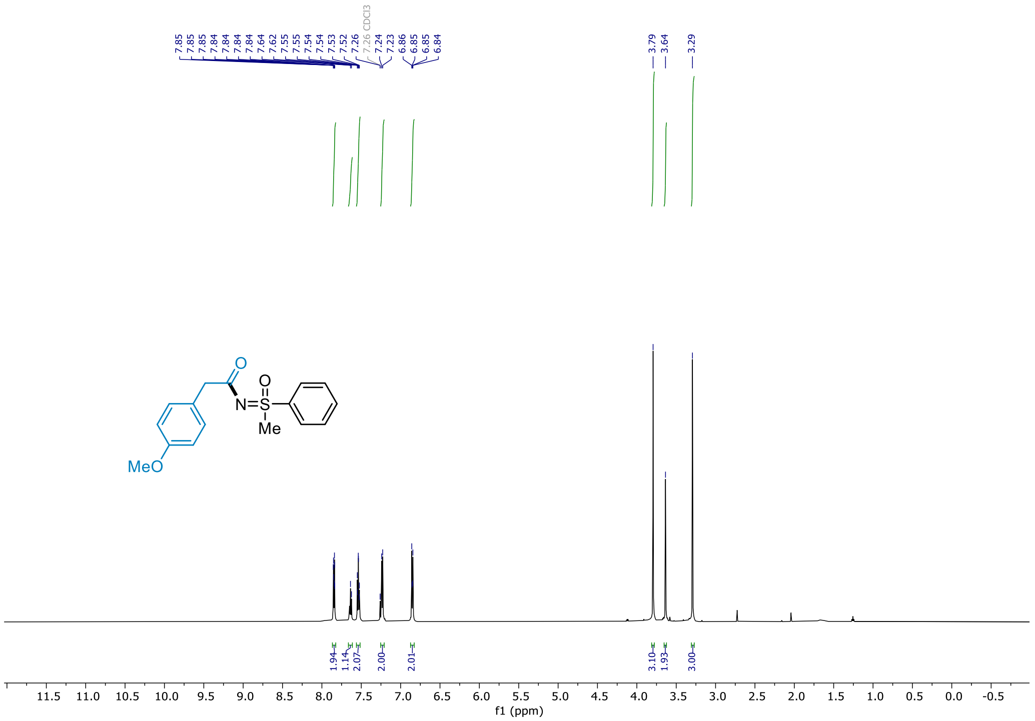

^{13}C NMR data of 2-(4-methoxyphenyl)-*N*-(methyl(oxo)(phenyl)- λ^{6}-sulfaneylidene)acetamide (15)

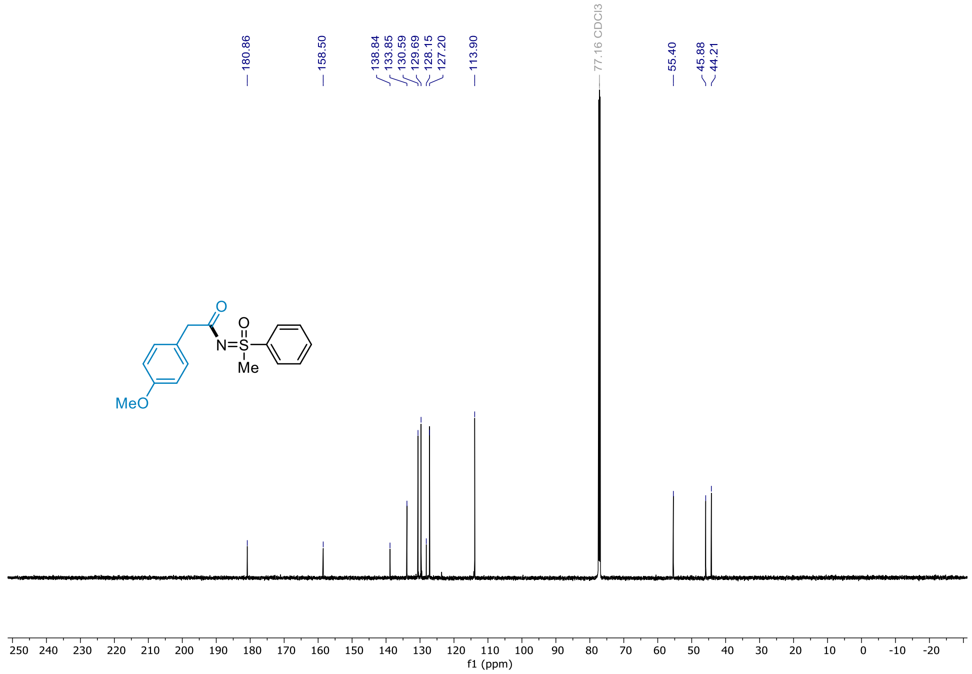

¹H NMR data of *N*-(dimethyl(oxo)- λ⁶-sulfaneylidene)-4-(trifluoromethyl)benzamide (16)

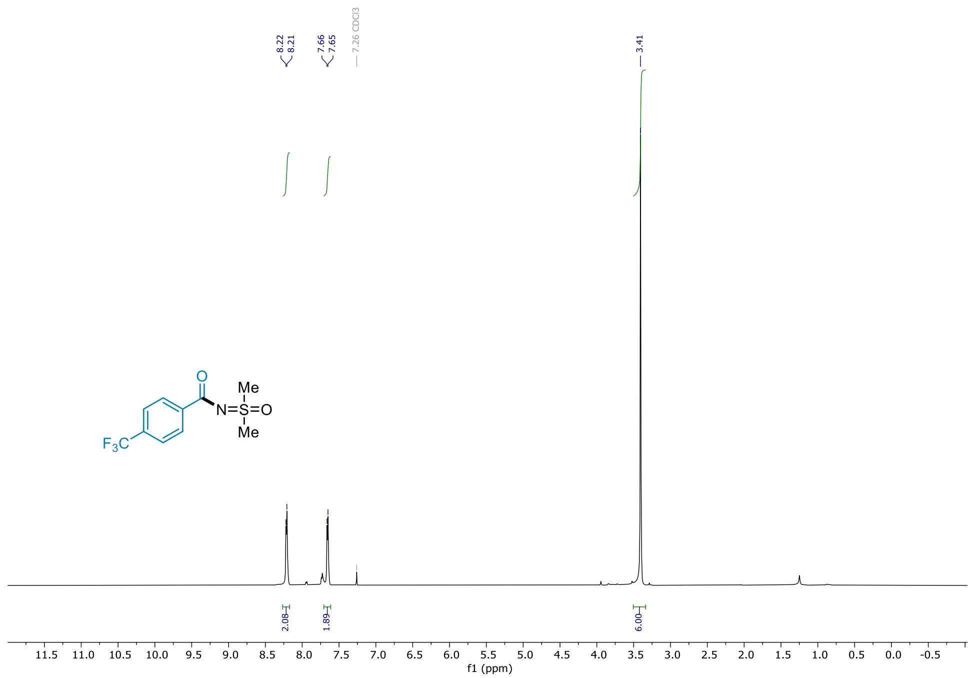

¹H NMR data of *N*-(methyl(oxo)(phenyl)- λ⁶-sulfaneylidene)-2-phenylacetamide (17)

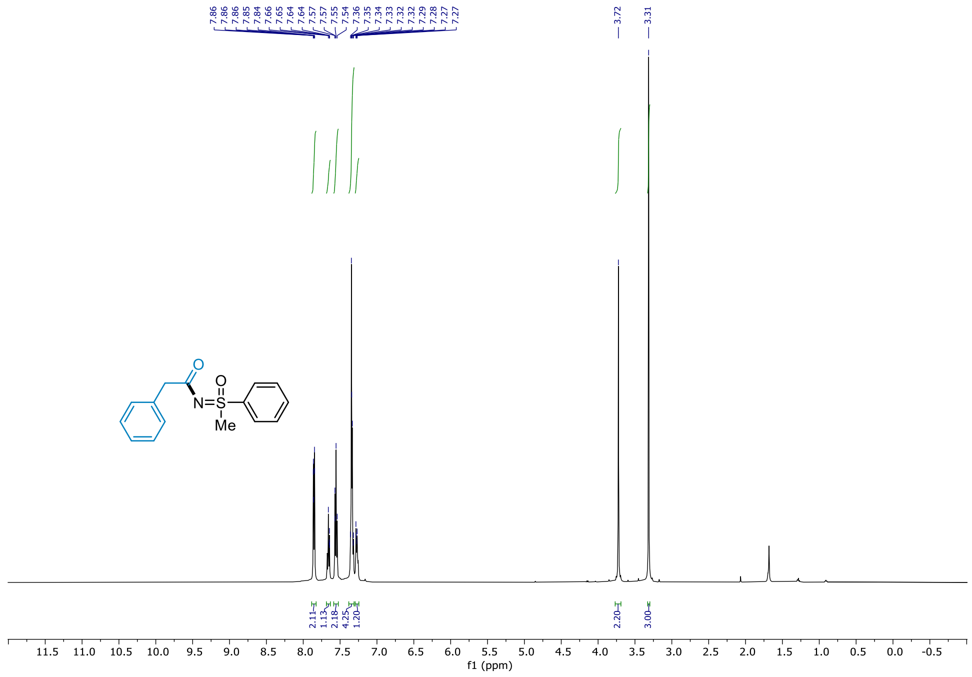

^{1}H NMR data of 2-(4-chlorophenyl)-*N*-(1-oxidotetrahydro- λ^{6}-thiophen-1-ylidene)acetamide (18)

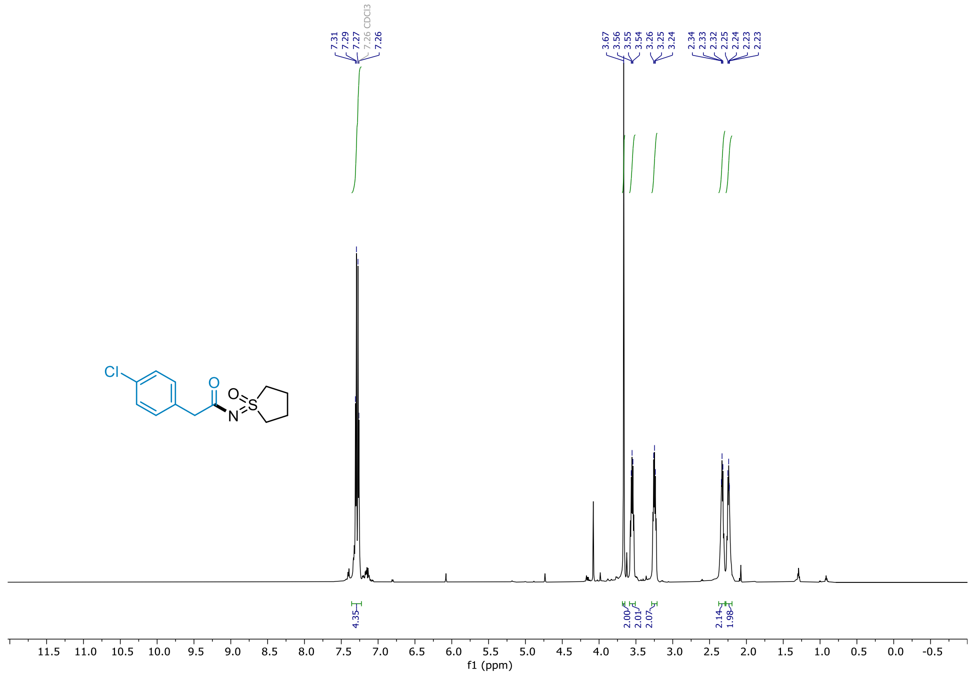

^{13}C NMR data of 2-(4-chlorophenyl)-*N*-(1-oxidotetrahydro- λ^{6}-thiophen-1-ylidene)acetamide (18)

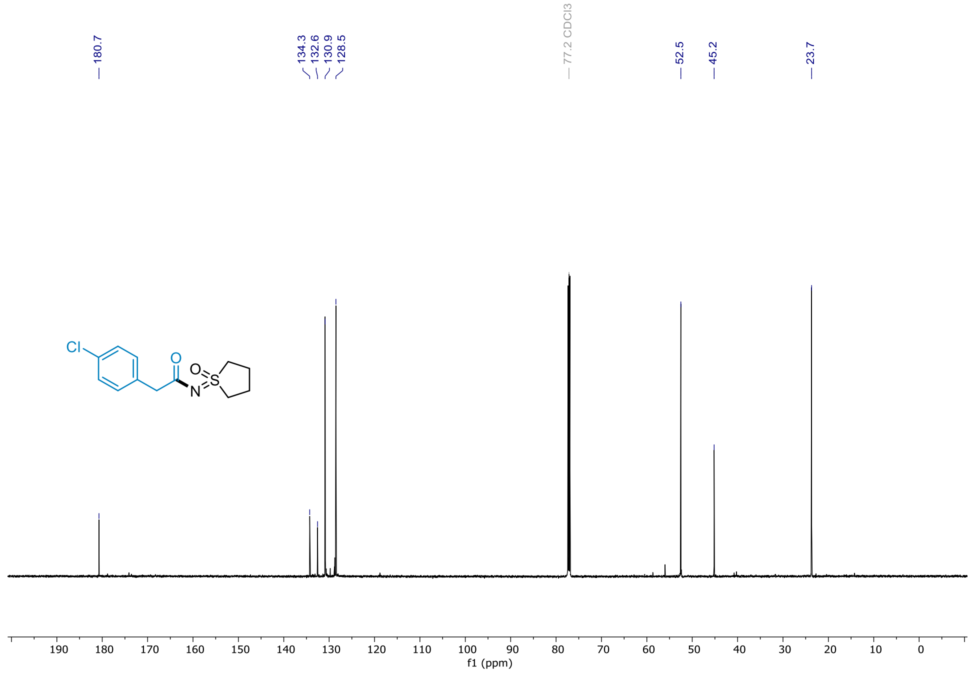

^{1}H NMR data of 4-fluoro-*N*-(1-oxidotetrahydro- λ^{6}-thiophen-1-ylidene)benzamide (19)

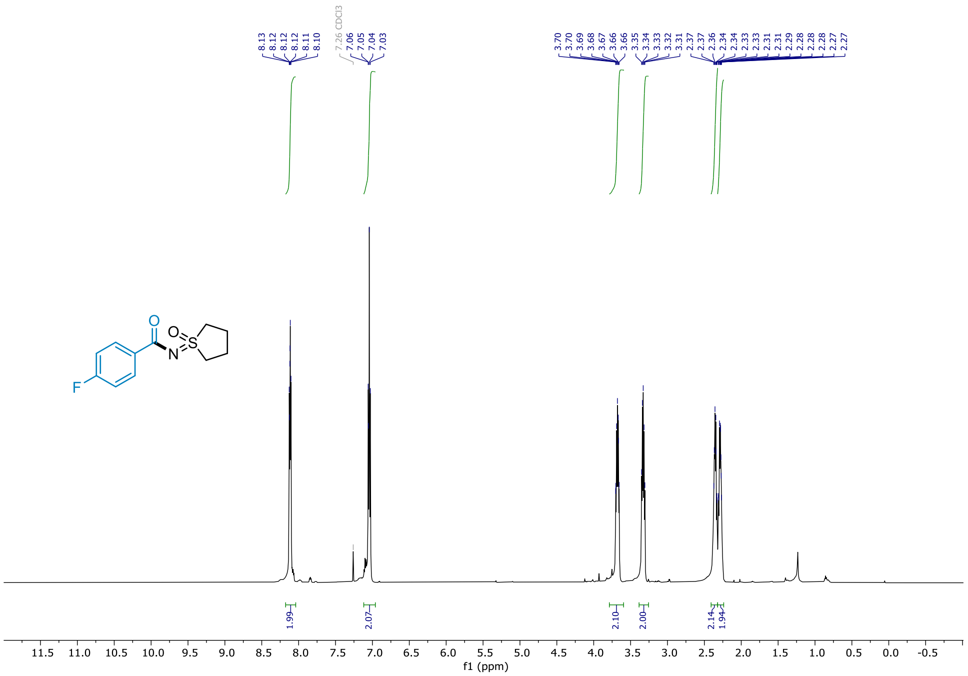

^{1}H NMR data of benzyl 3-((methyl(oxo)(phenyl)- λ^{6}-sulfaneylidene)carbamoyl)piperidine-1-carboxylate (20)

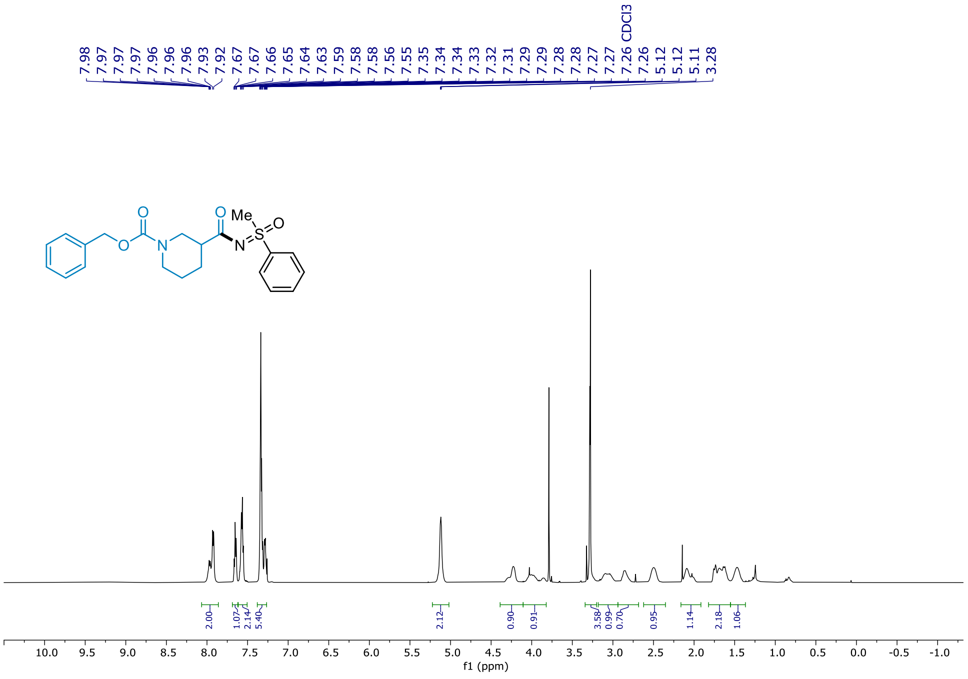

^{1}H NMR data of benzyl 3-((methyl(oxo)(phenyl)- λ[6]-sulfaneylidene)carbamoyl)piperidine-1-carboxylate (20)

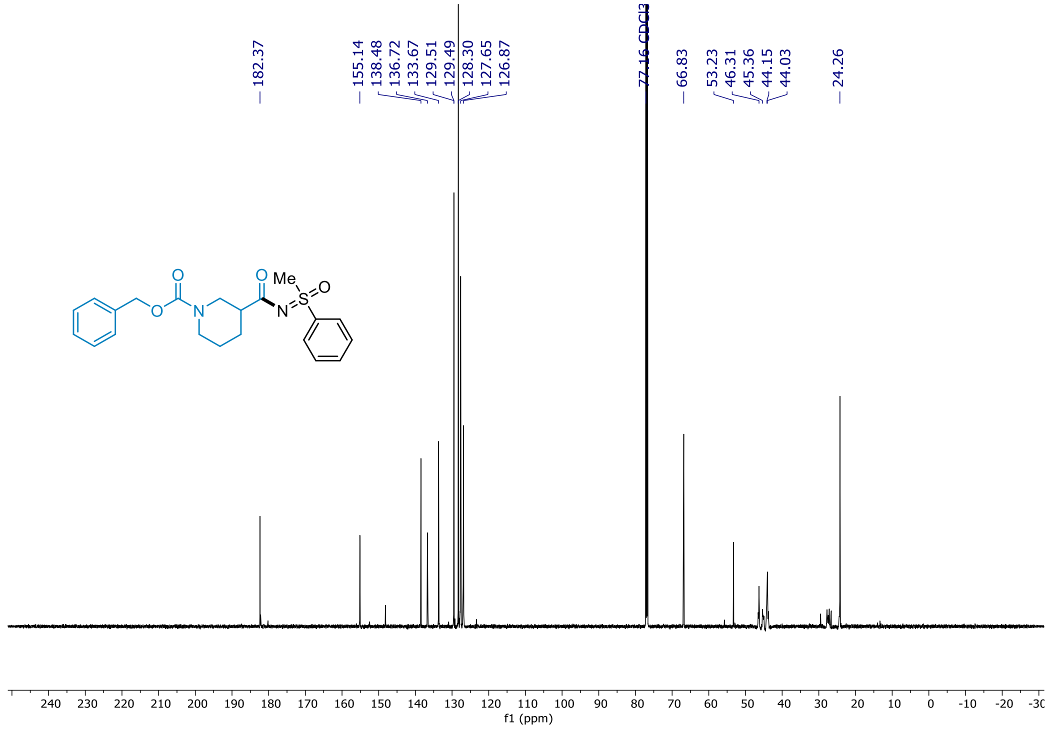

^{1}H NMR data of *tert*-butyl (3-(4-((((2-bromobenzyl)oxy)carbonyl)oxy)phenyl)-1-((1-oxidotetrahydro- λ[6]-thiophen-1-ylidene)amino)-1-oxopropan-2-yl)carbamate (21)

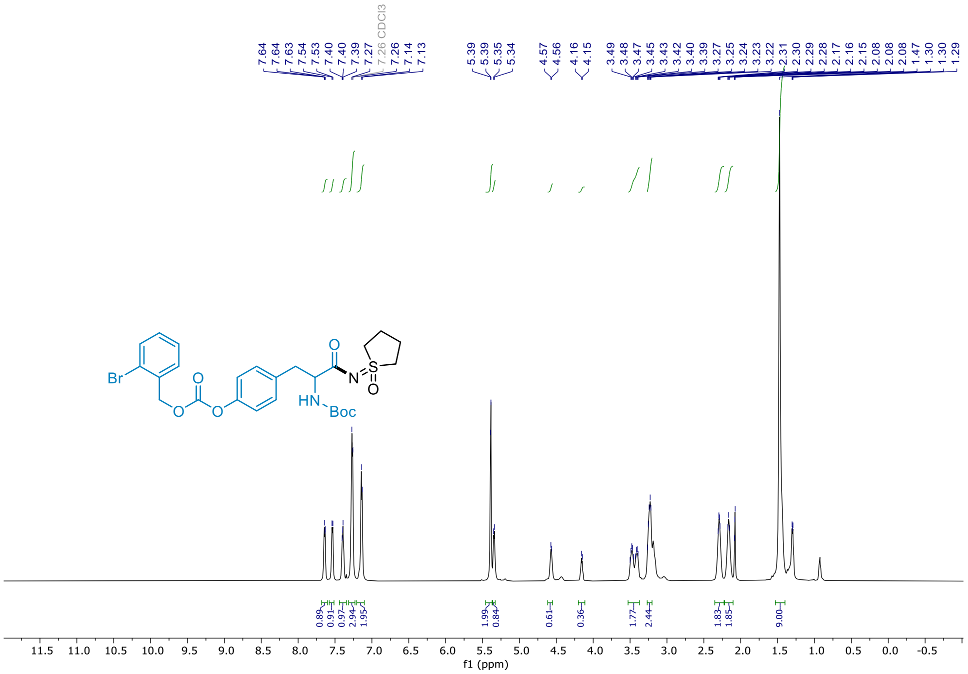

^{13}C NMR data of *tert*-butyl (3-(4-((((2-bromobenzyl)oxy)carbonyl)oxy)phenyl)-1-((1-oxidotetrahydro- λ^6-thiophen-1-ylidene)amino)-1-oxopropan-2-yl)carbamate (21)

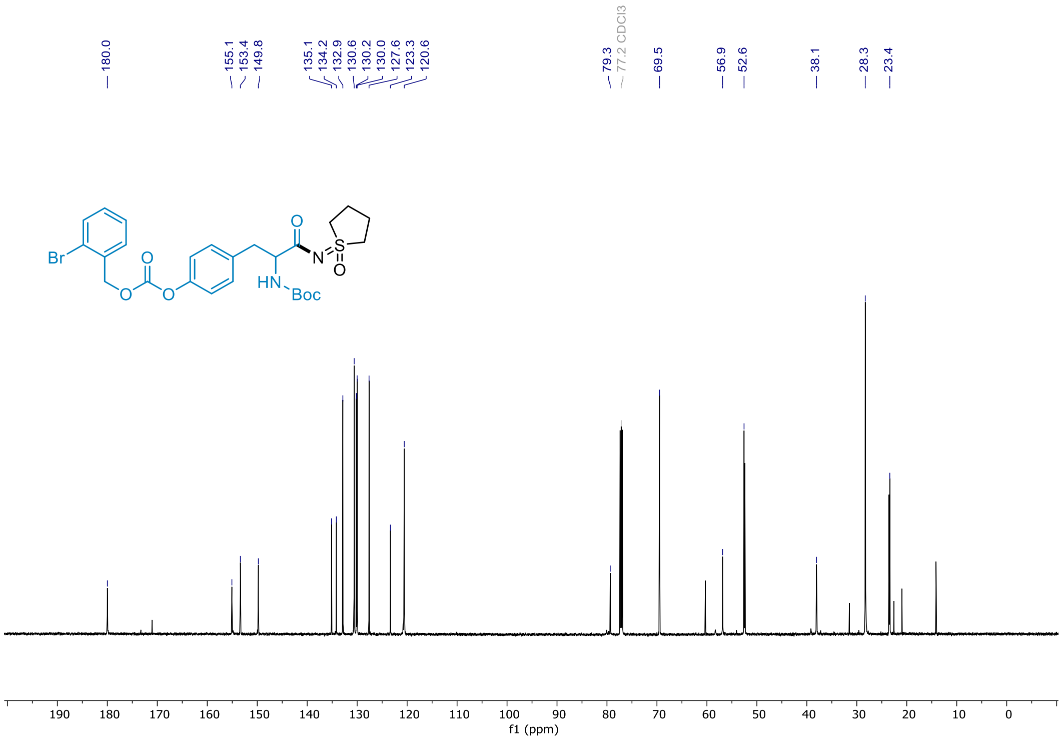

^{1}H NMR data of *N*-(1-oxidotetrahydro- λ^6-thiophen-1-ylidene)-2,2-diphenylacetamide (22)

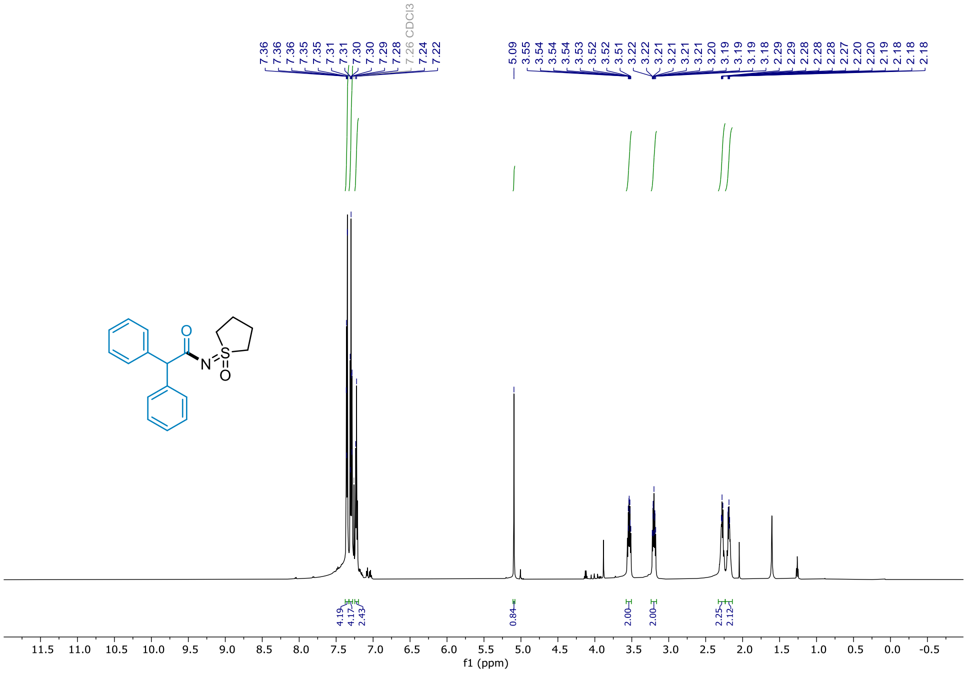

^{13}C NMR data of *N*-(1-oxidotetrahydro- λ^6-thiophen-1-ylidene)-2,2-diphenylacetamide (22)

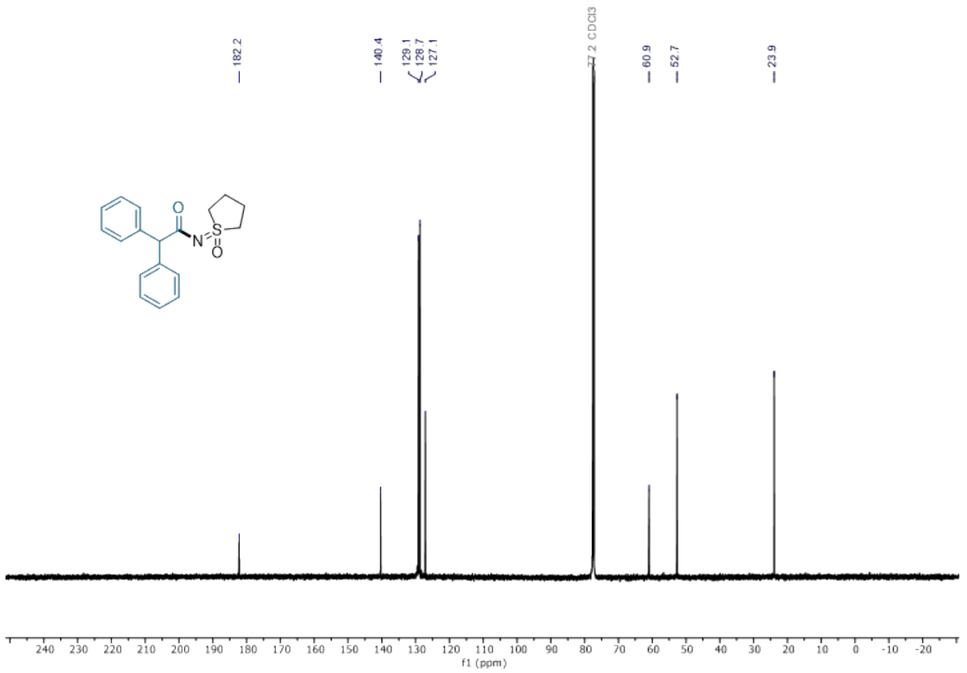

^{1}H NMR data of benzyl (1-((1-oxidotetrahydro-1l6-thiophen-1-ylidene)amino)-1-oxobutan-2-yl)carbamate (23)

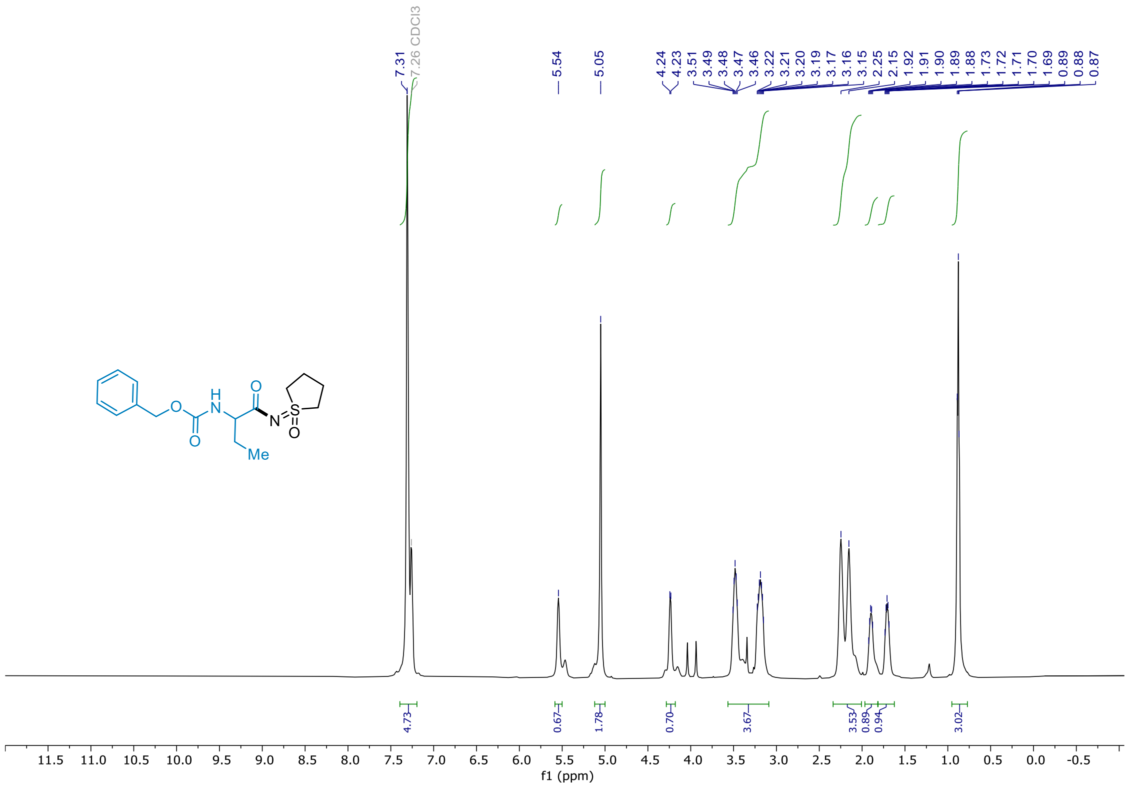

^{13}C NMR data of benzyl (1-((1-oxidotetrahydro-1l6-thiophen-1-ylidene)amino)-1-oxobutan-2-yl)carbamate (23)

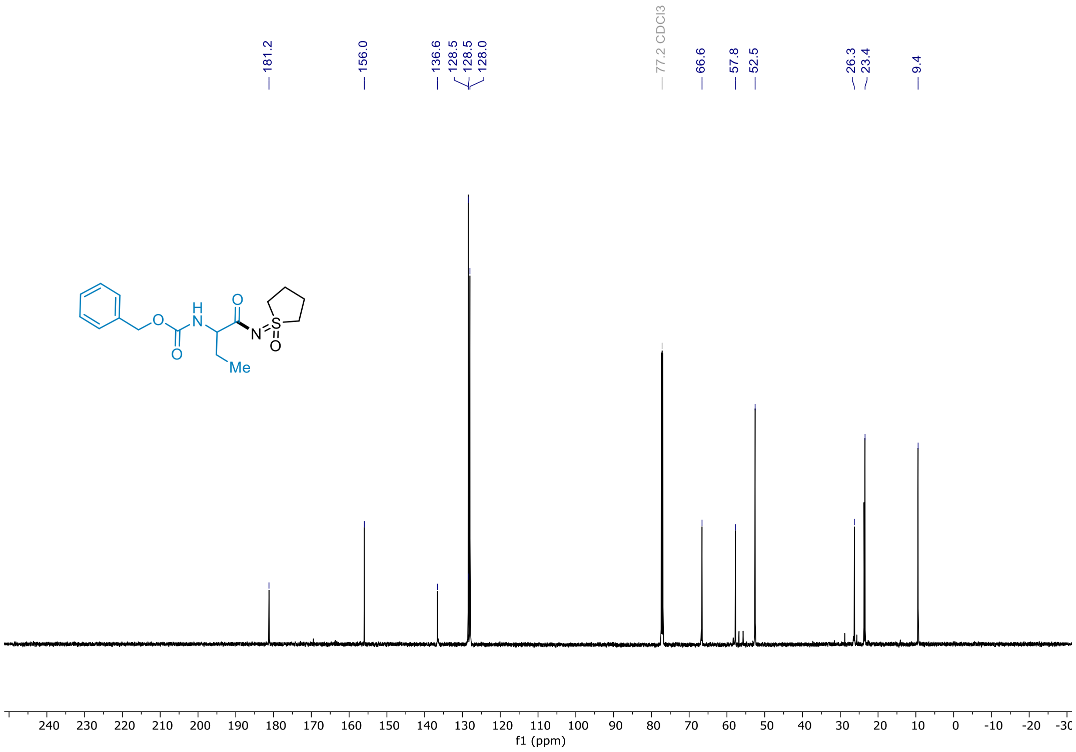

^{1}H NMR data of *N*-(methyl(oxo)(phenyl)- λ^{6}-sulfaneylidene)-3-(1-methyl-1H-indol-3-yl)propanamide (24)

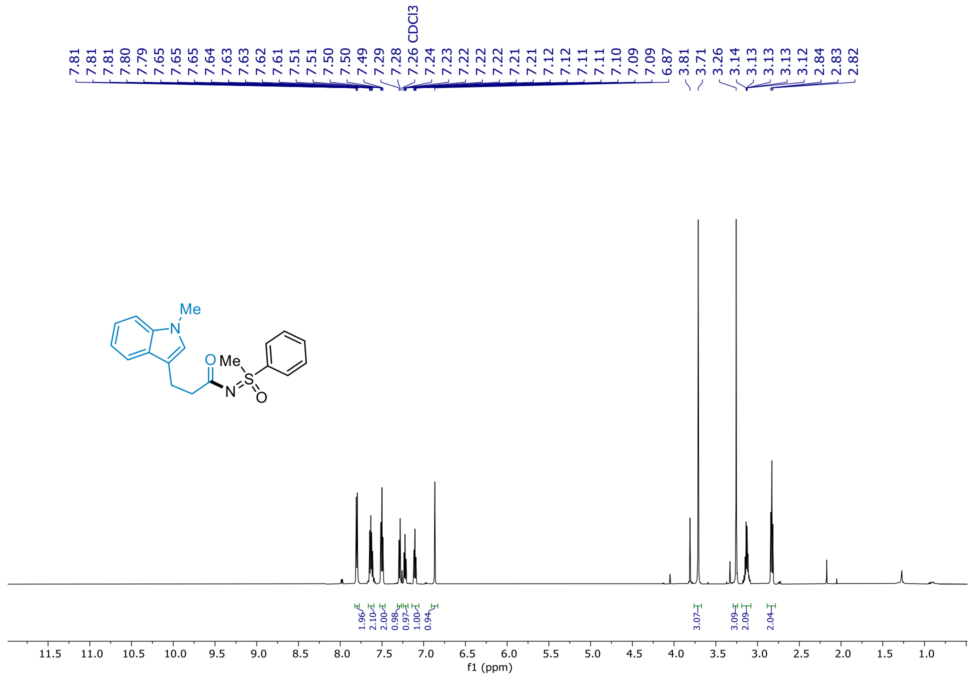

^{13}C NMR data of *N*-(methyl(oxo)(phenyl)- λ^6-sulfaneylidene)-3-(1-methyl-1H-indol-3-yl)propanamide (24)

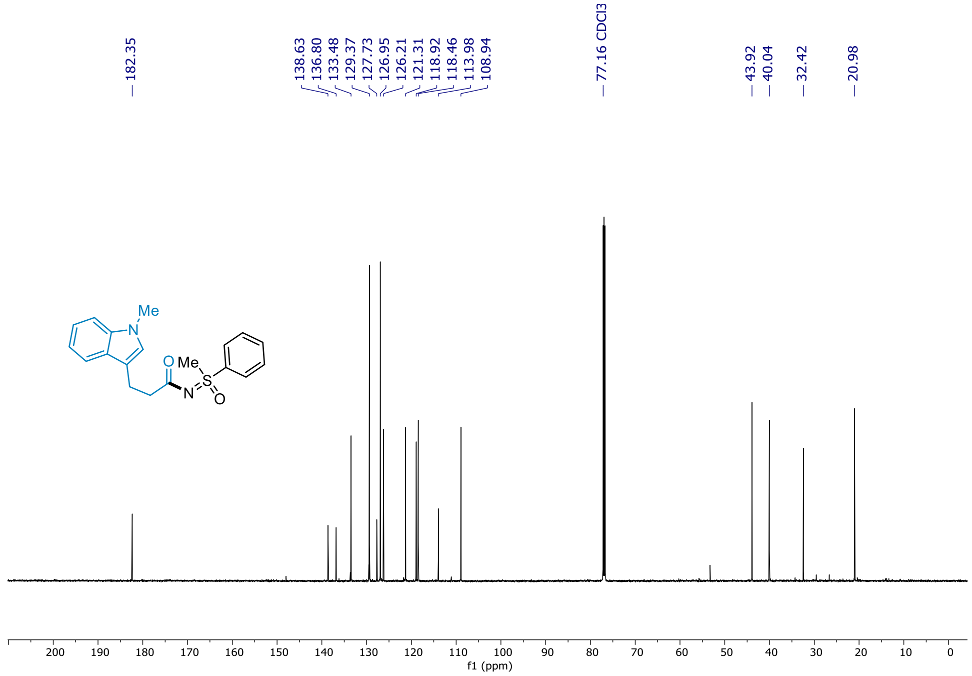

^{1}H NMR data of *tert*-butyl(1-((1-oxidotetrahydro-1l6-thiophen-1-ylidene)amino)-1-oxo-3-phenylpropan-2-yl)carbamate (25)

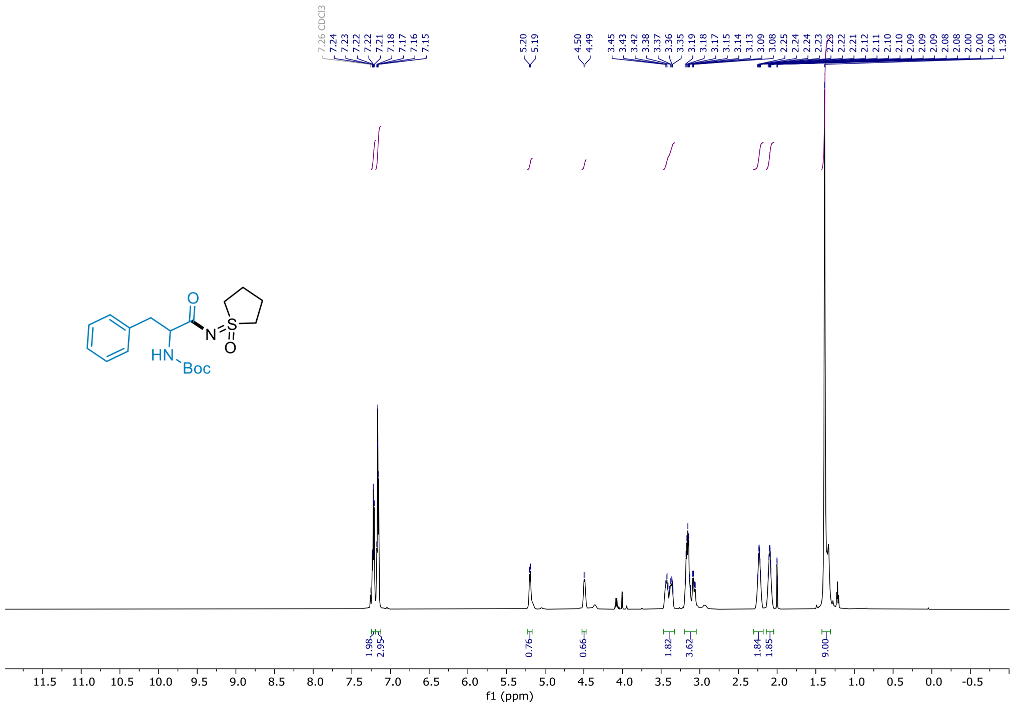

^{13}C NMR data of *tert*-butyl(1-((1-oxidotetrahydro-1l6-thiophen-1-ylidene)amino)-1-oxo-3-phenylpropan-2-yl)carbamate (25)

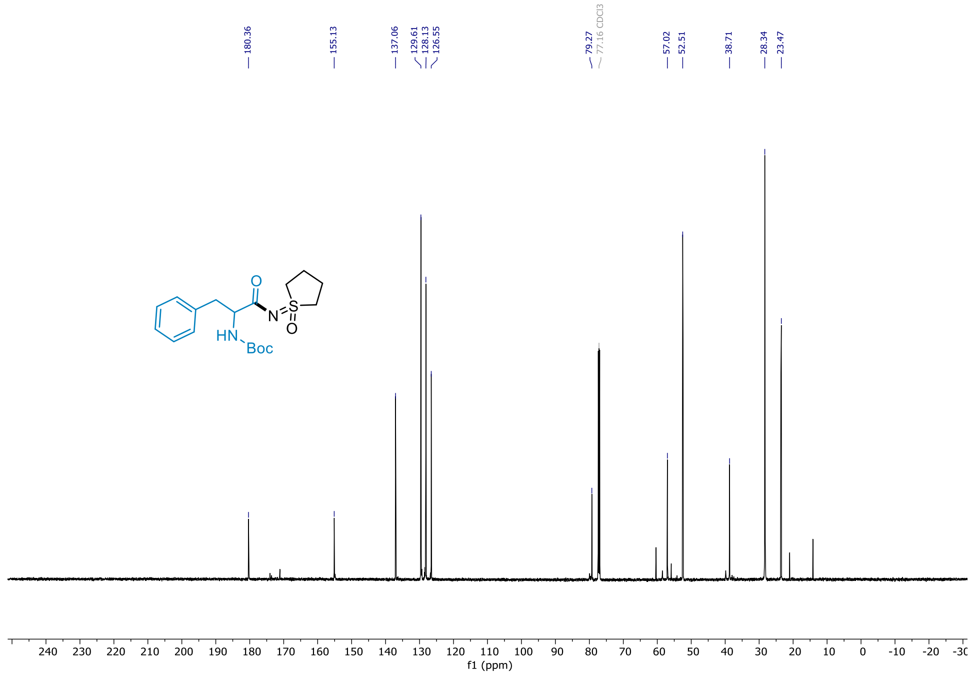

^1H NMR data of *tert*-butyl(1-((methyl(oxo)(phenyl)-λ^6-sulfaneylidene)amino)-1-oxo-3-phenylpropan-2-yl)carbamate (26)

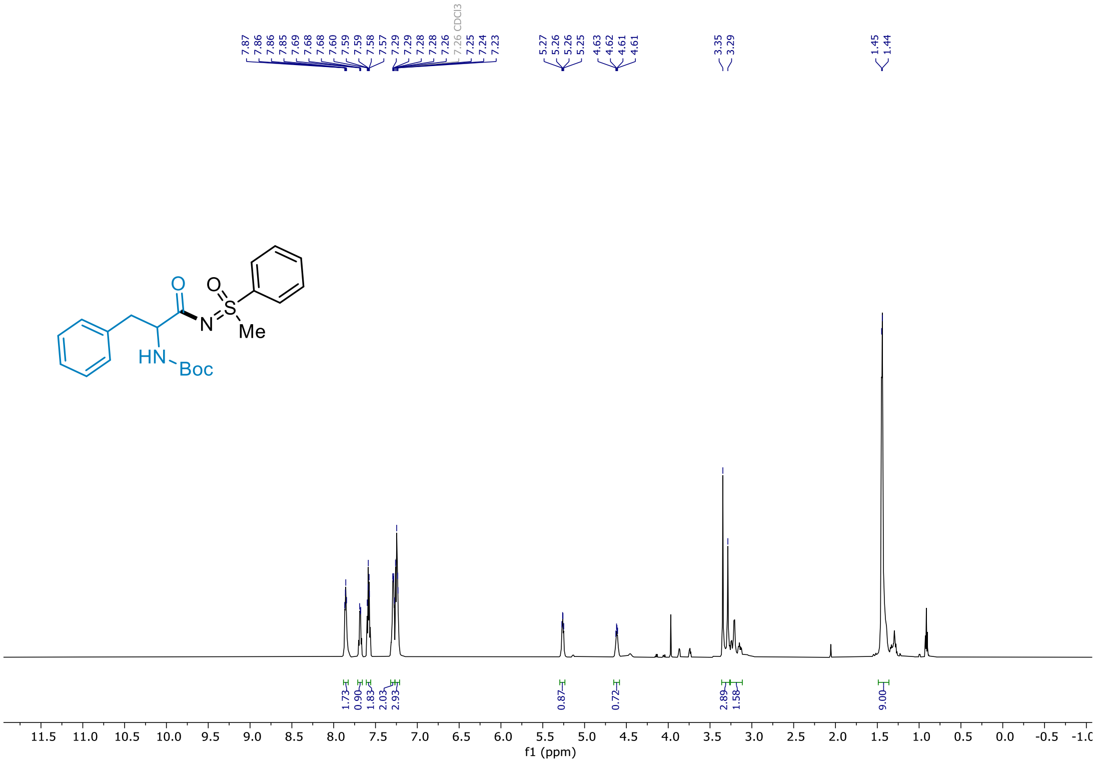

^{1}H NMR data of *N*-(dimethyl(oxo)- λ^{6}-sulfaneylidene)-2,2-diphenylacetamide (27)

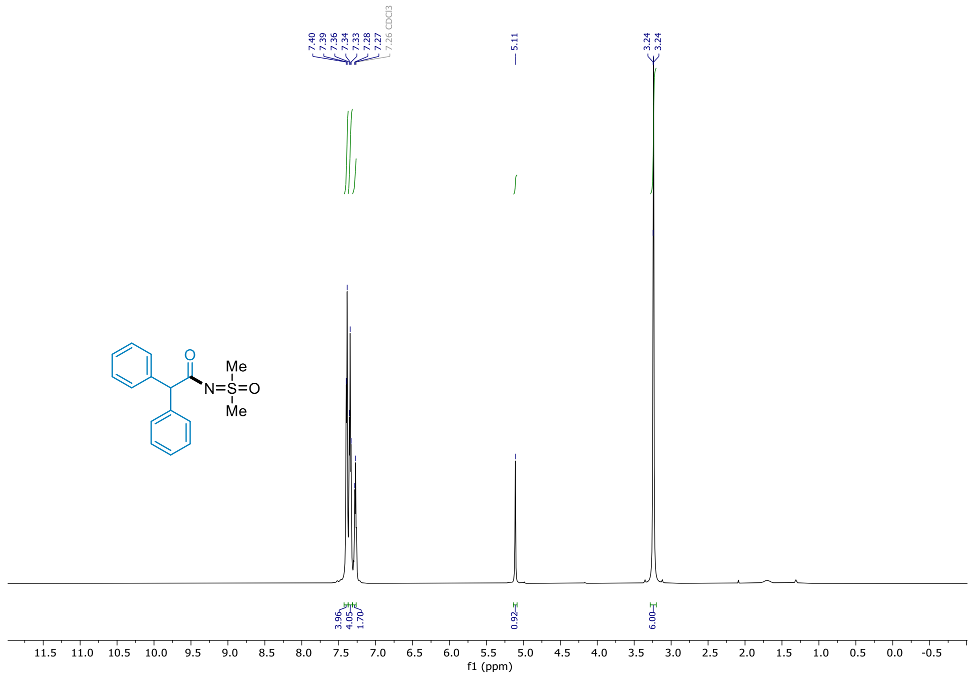

^{1}H NMR data of *N*-(dimethyl(oxo)- λ^{6}-sulfaneylidene)-2,2-diphenylacetamide (27)

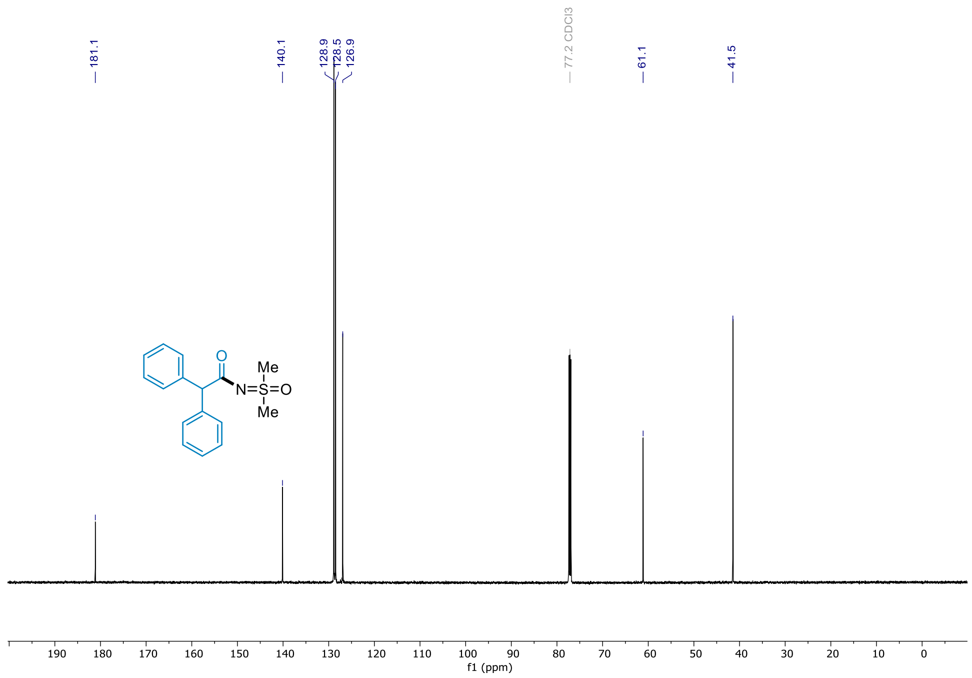

^{1}H NMR data of *N*-(dimethyl(oxo)- λ^{6}-sulfaneylidene)cyclohex-1-ene-1-carboxamide (28)

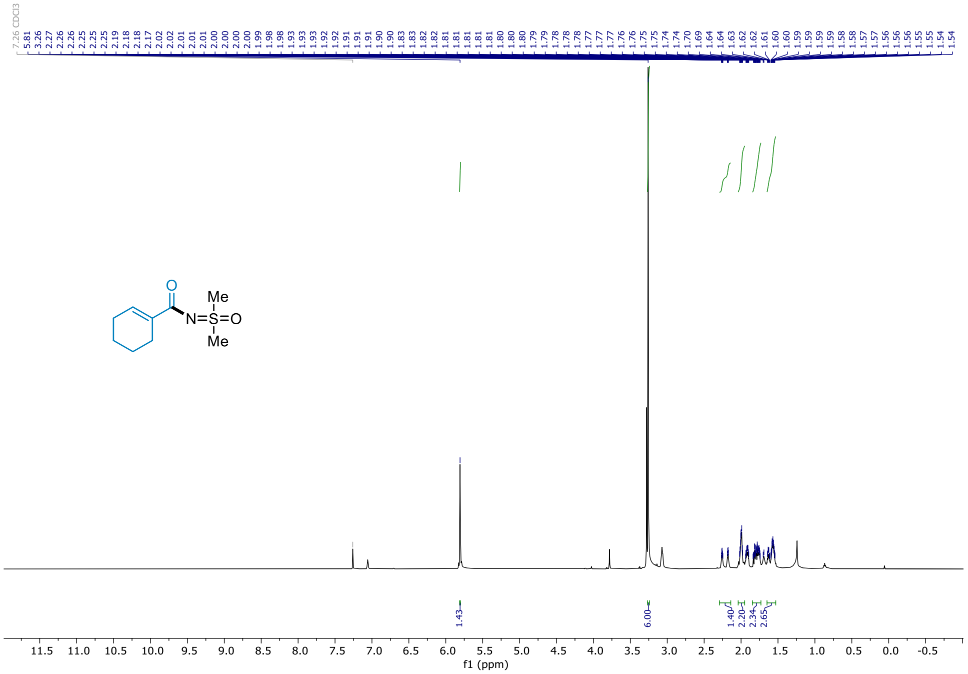

^{13}C NMR data of *N*-(dimethyl(oxo)- λ^{6}-sulfaneylidene)cyclohex-1-ene-1-carboxamide (28)

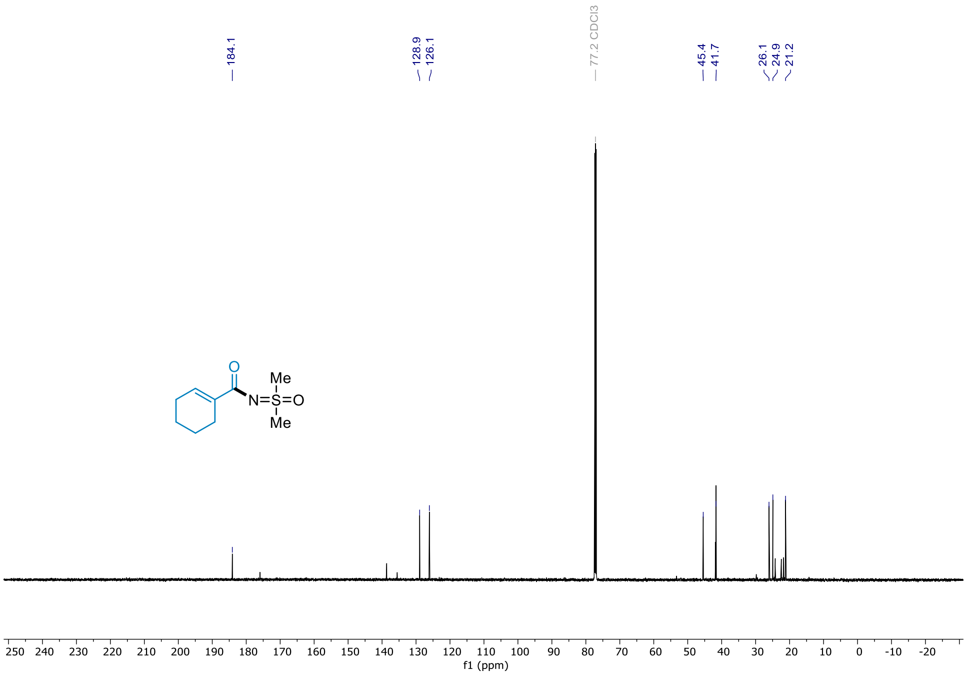

^{1}H NMR data of *N*-(dimethyl(oxo)- λ[6]-sulfaneylidene)benzamide (29)

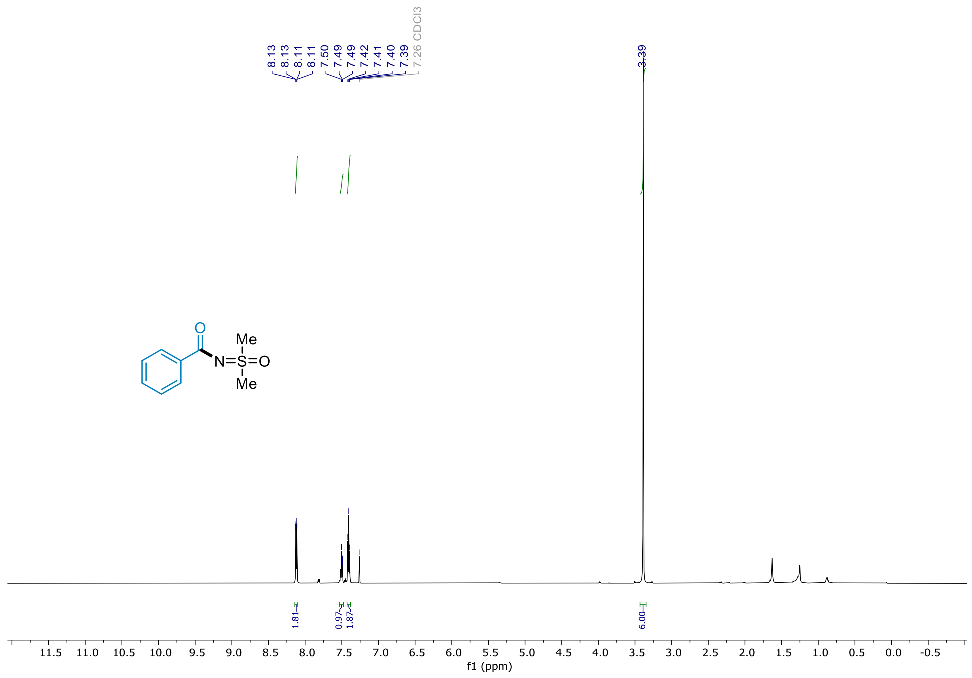

^{1}H NMR data of *N*-(dimethyl(oxo)- λ[6]-sulfaneylidene)-2-oxo-2-phenylacetamide (30)

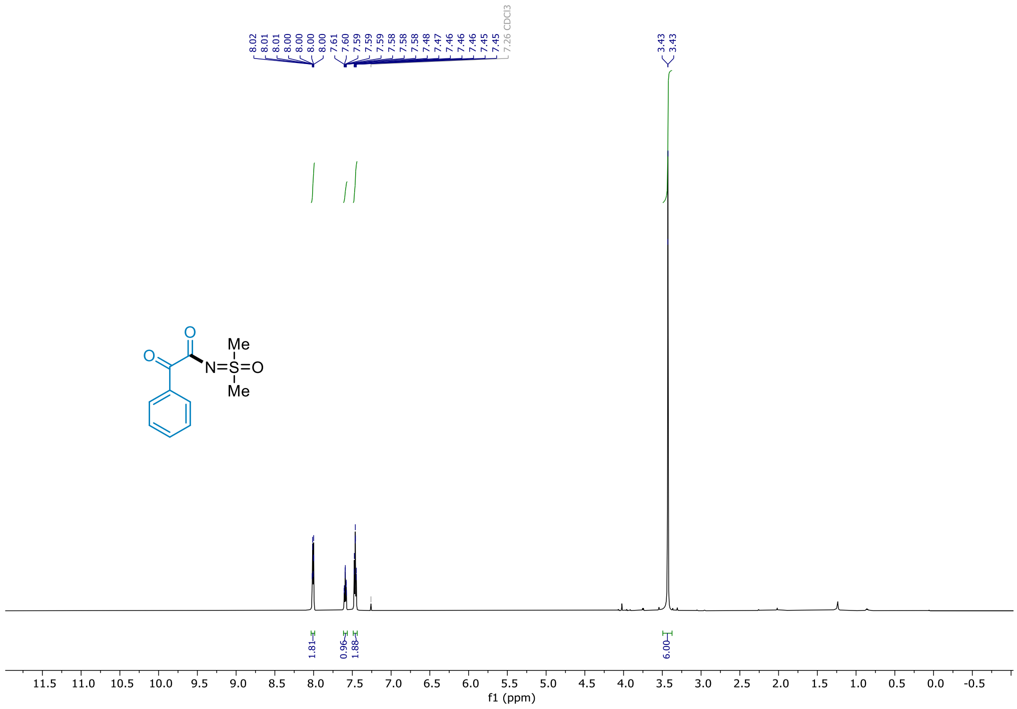

^{1}H NMR data of *N*-(dimethyl(oxo)- λ[6]-sulfaneylidene)-4-ethynylbenzamide (31)

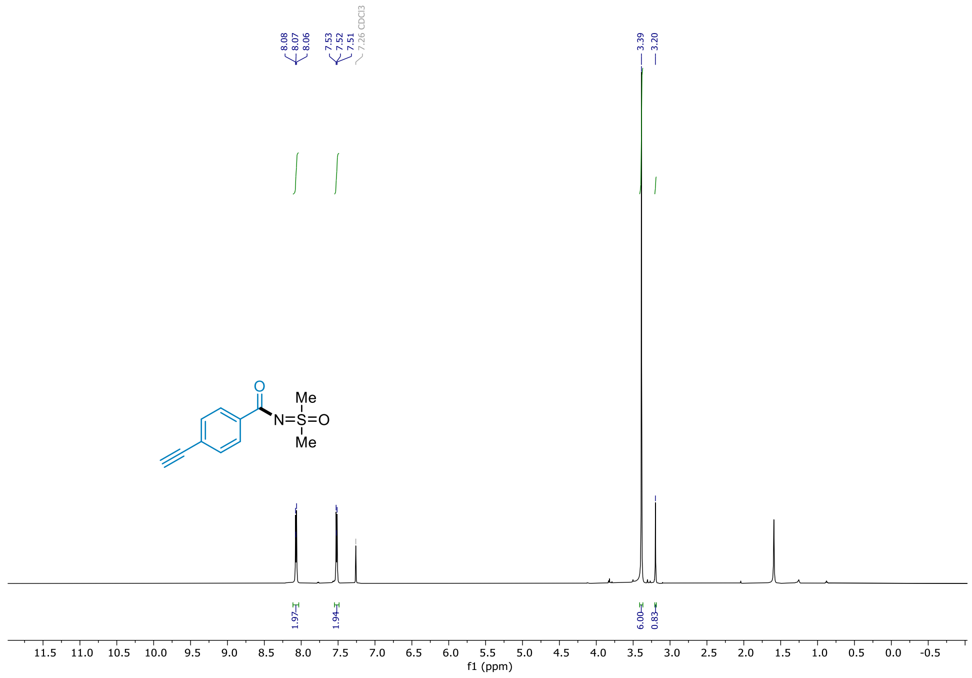

^{13}C NMR data of *N*-(dimethyl(oxo)- λ[6]-sulfaneylidene)-4-ethynylbenzamide (31)

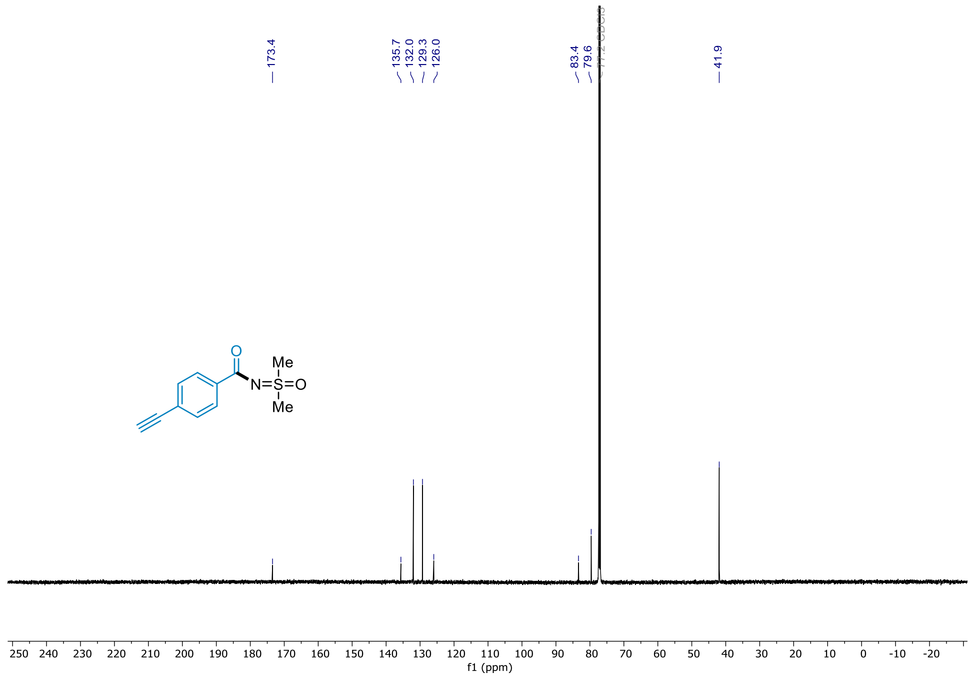

^{1}H NMR data of *N*-(dimethyl(oxo)- λ[6]-sulfaneylidene)-2-phenylpropanamide (32)

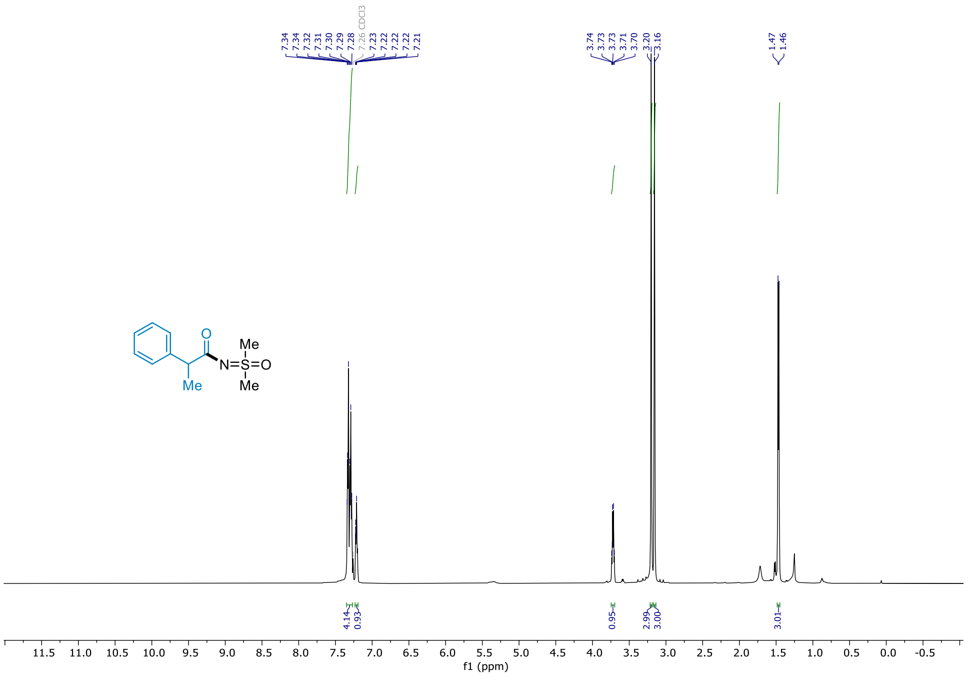

^{13}C NMR data of *N*-(dimethyl(oxo)- λ[6]-sulfaneylidene)-2-phenylpropanamide (32)

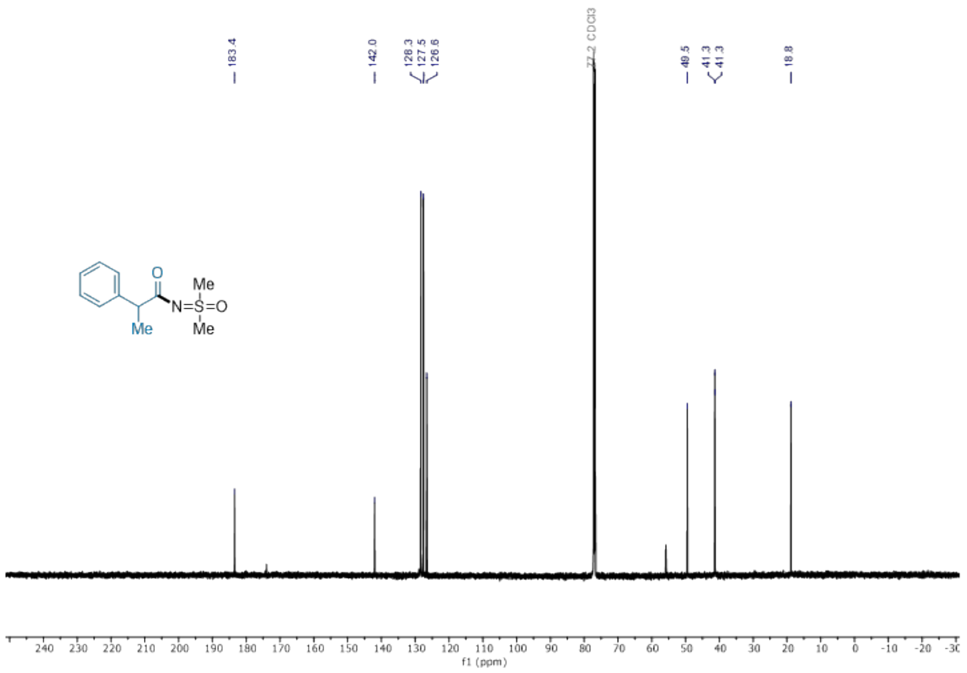

12. HRMS DATA

In addition to experimental HRMS, we provided all calculated/predicted isotopic distributions on the right side of each spectrum

2-(4-Fluorophenyl)-*N*-(methyl(oxo)(phenyl)- λ^6-sulfaneylidene)acetamide (6)

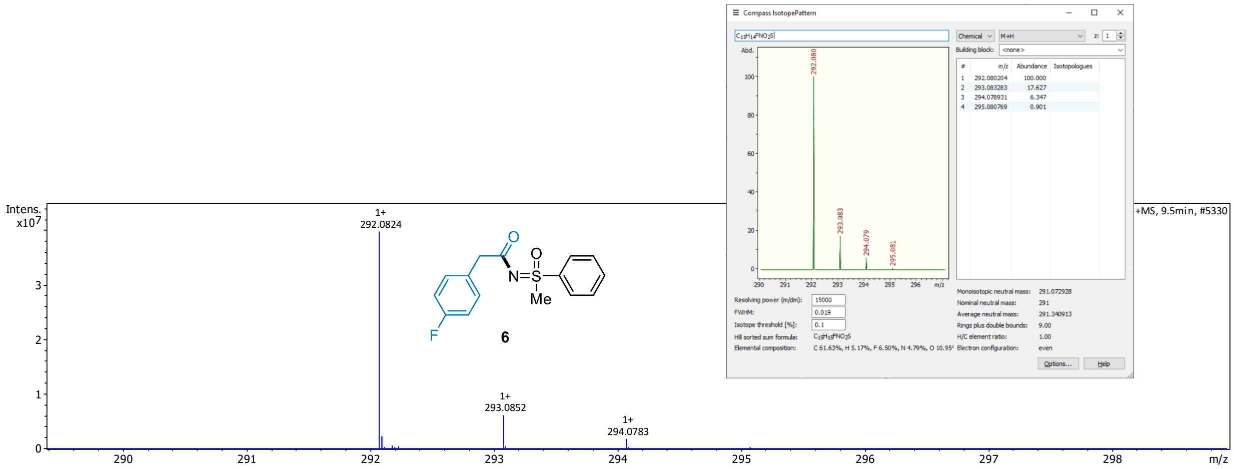

10-Bromo-*N*-(methyl(oxo)(phenyl)-λ^6-sulfaneylidene)decanamide (9)

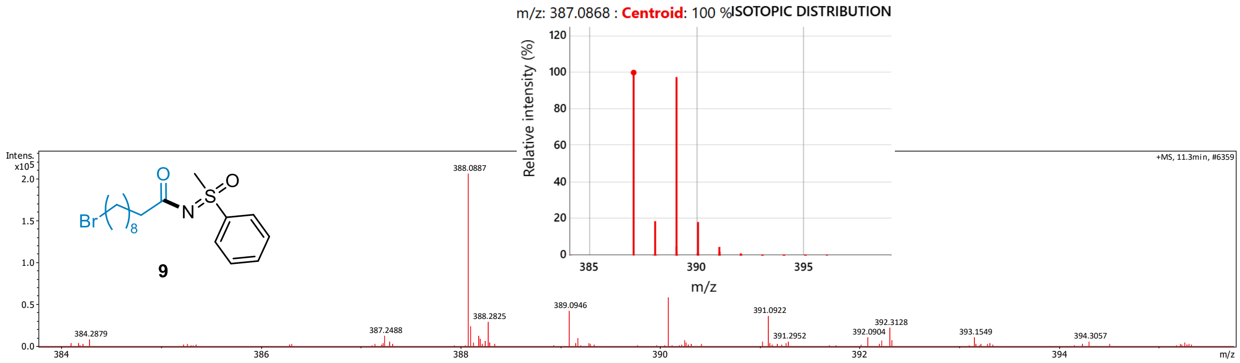

10-Bromo-*N*-(oxodiphenyl- λ⁶-sulfaneylidene)decanamide (10)

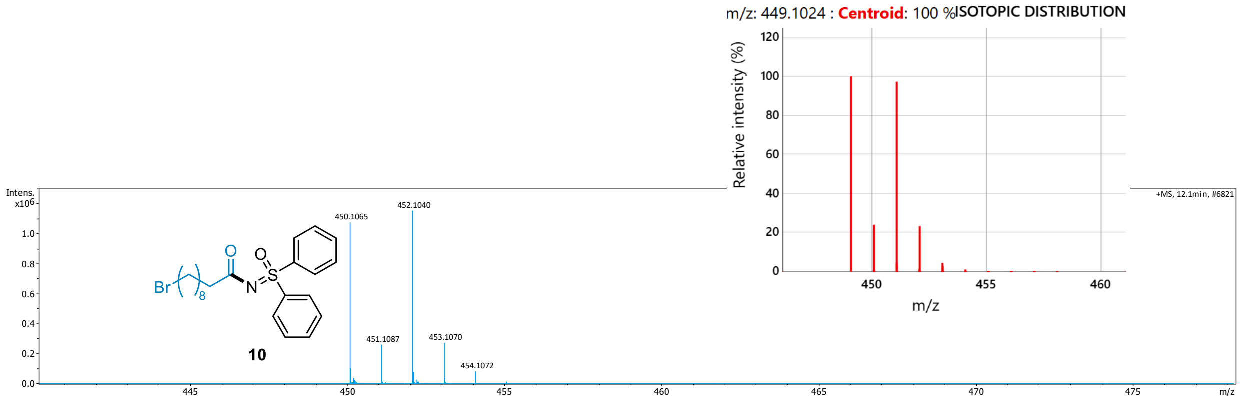

N-(Methyl(oxo)(phenyl)- λ⁶-sulfaneylidene)pent-4-enamide (11)

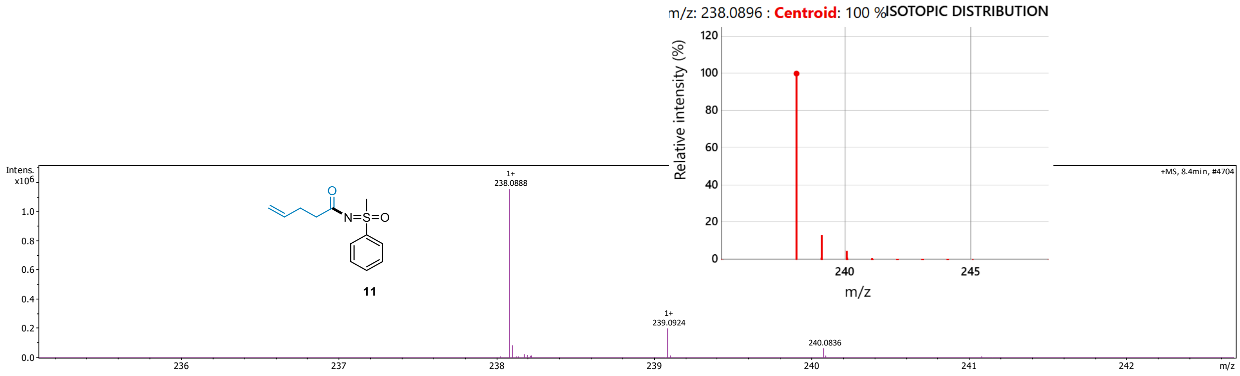

N-(Oxodiphenyl- λ⁶-sulfaneylidene)pent-4-enamide (12)

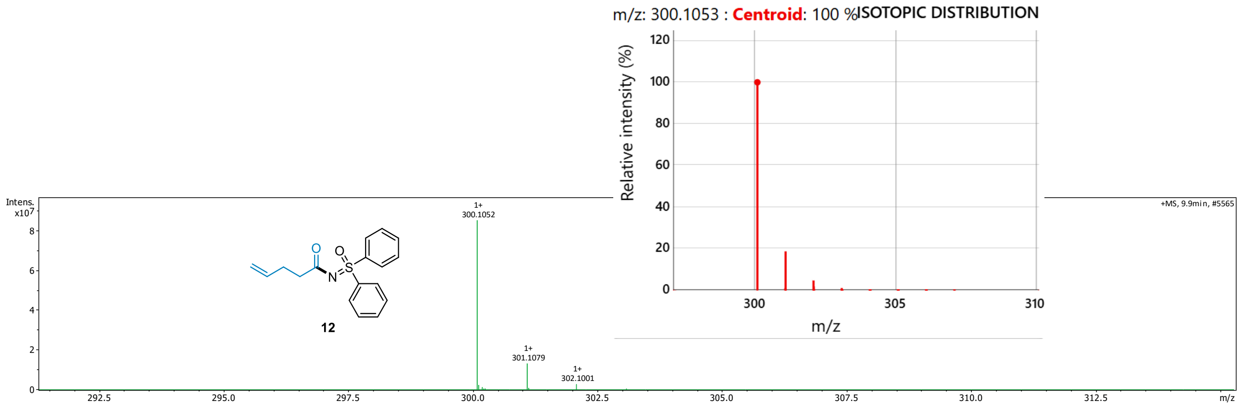

4,4,4-Trifluoro-*N*-(methyl(oxo)(phenyl)- λ^6-sulfaneylidene)butanamide (14)

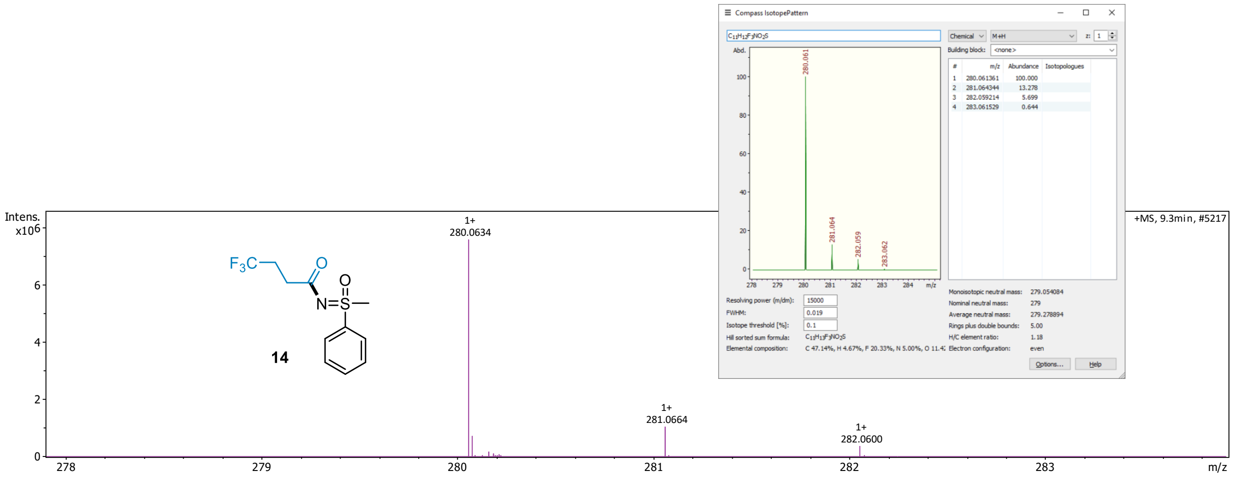

2-(4-Methoxyphenyl)-*N*-(methyl(oxo)(phenyl)- λ^6-sulfaneylidene)acetamide (15)

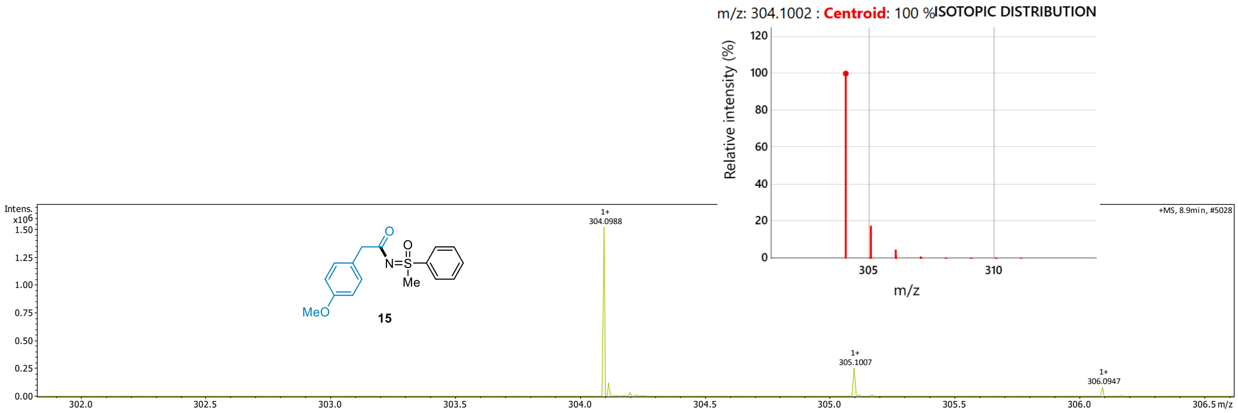

2-(4-Chlorophenyl)-*N*-(1-oxidotetrahydro- λ^6-thiophen-1-ylidene)acetamide (18)

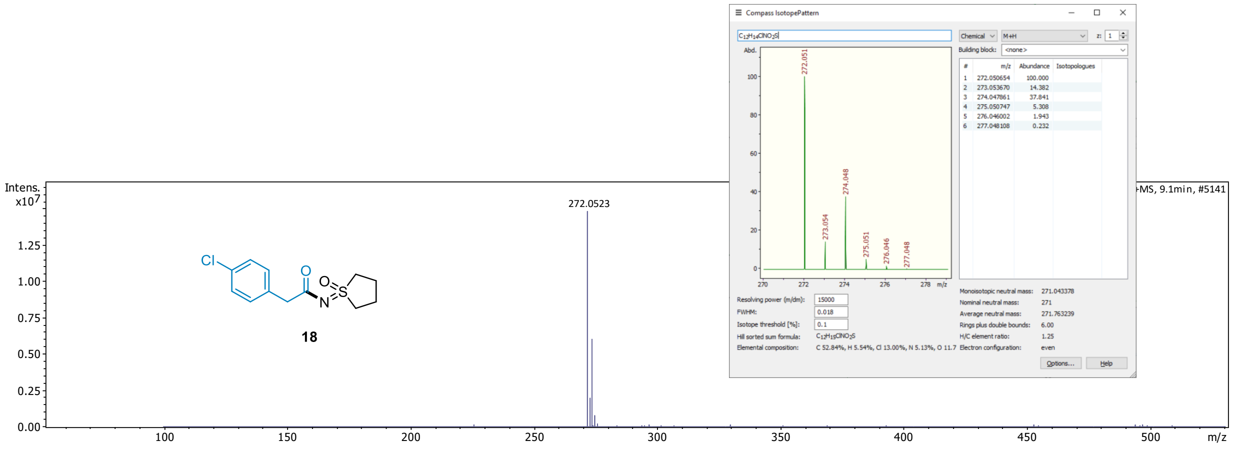

Benzyl 3-((methyl(oxo)(phenyl)- λ6-sulfaneylidene)carbamoyl)piperidine-1-carboxylate (20)

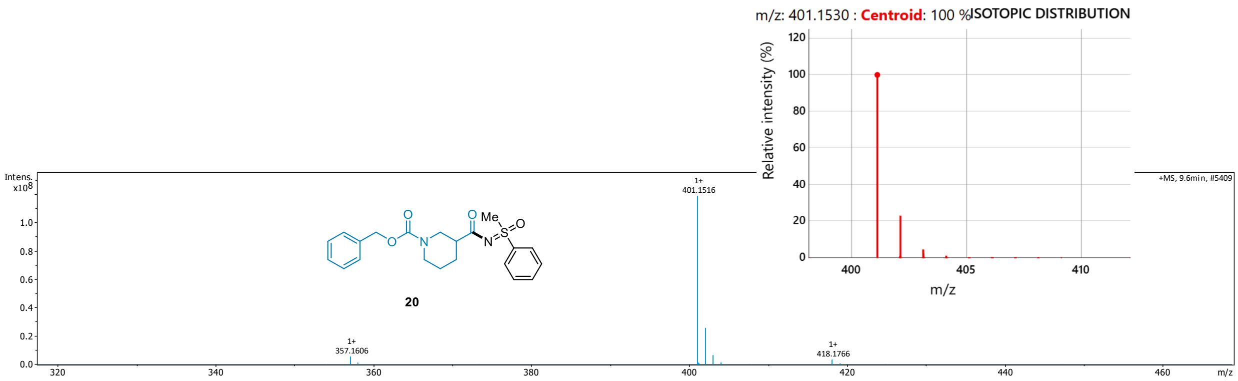

***Tert*-butyl (3-(4-((((2-bromobenzyl)oxy)carbonyl)oxy)phenyl)-1-((1-oxidotetrahydro- λ6-thiophen-1-ylidene)amino)-1-oxopropan-2-yl)carbamate (21)**

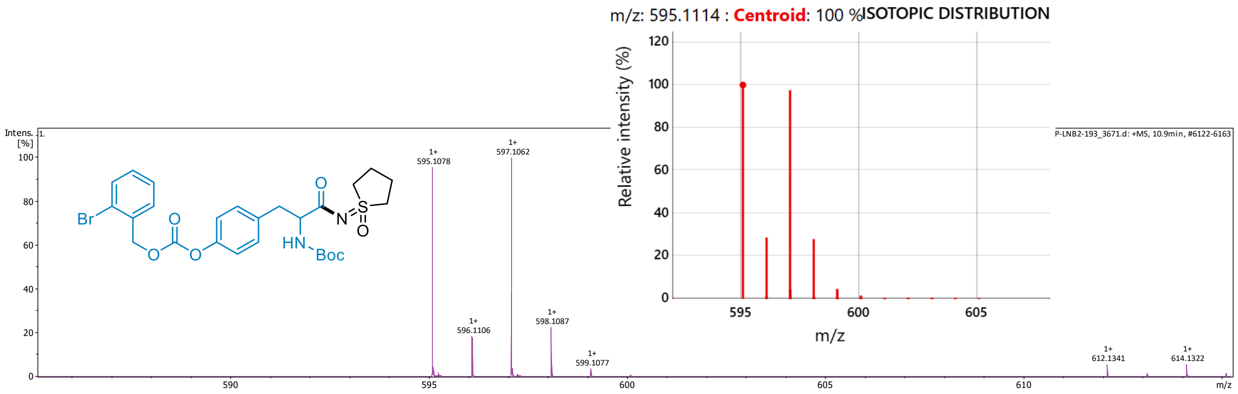

***N*-(1-Oxidotetrahydro- λ6-thiophen-1-ylidene)-2,2-diphenylacetamide (22)**

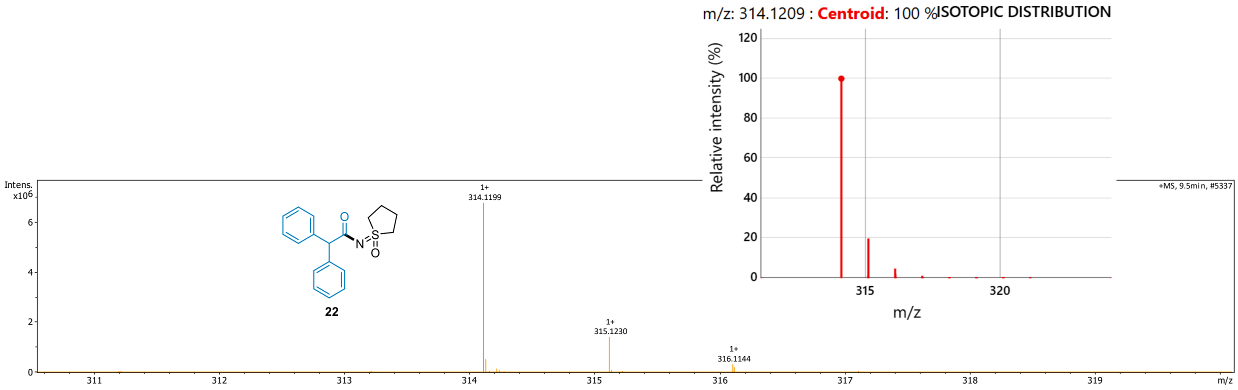

Benzyl (1-((1-oxidotetrahydro-1l6-thiophen-1-ylidene)amino)-1-oxobutan-2-yl)carbamate (23)

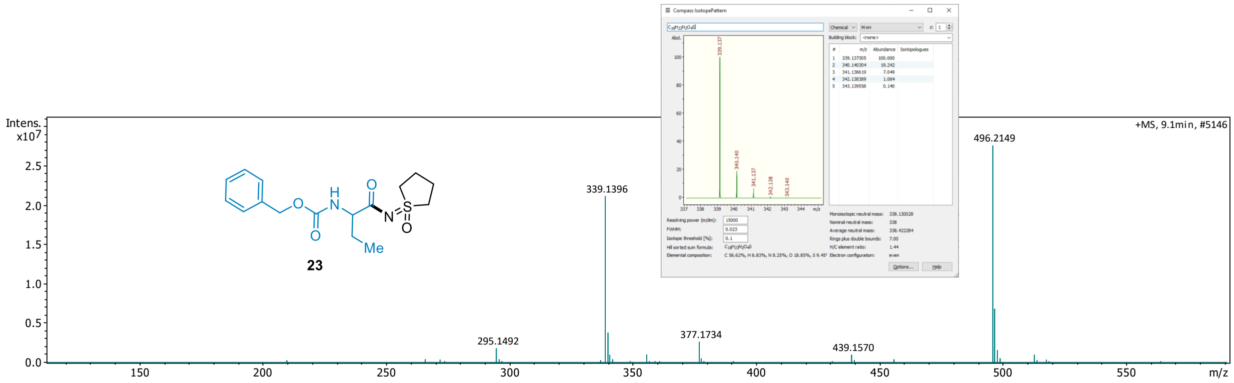

***N*-(methyl(oxo)(phenyl)- λ^6-sulfaneylidene)-3-(1-methyl-1H-indol-3-yl)propanamide (24)**

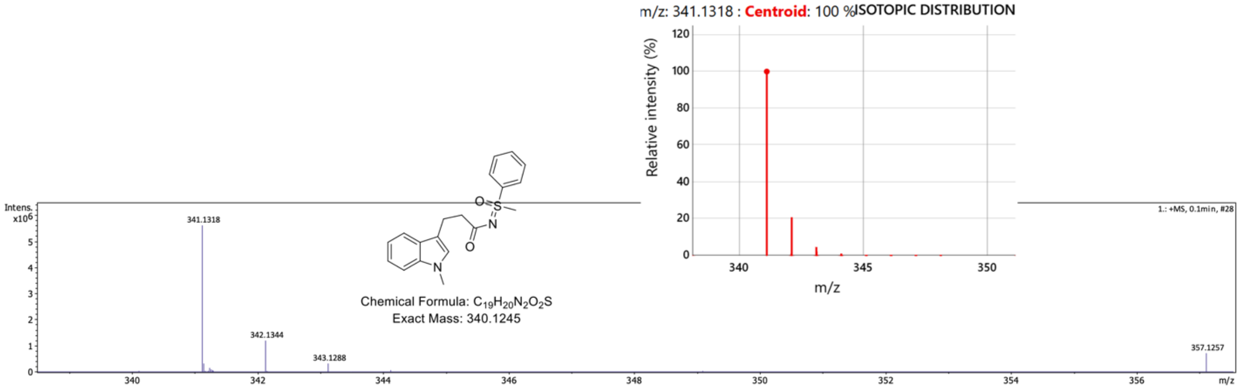

***Tert*-butyl(1-((1-oxidotetrahydro-1l6-thiophen-1-ylidene)amino)-1-oxo-3-phenylpropan-2-yl)carbamate (25)**

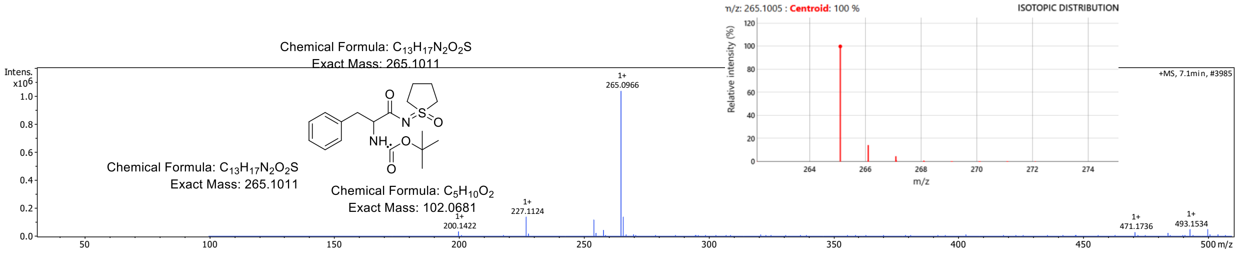

N-(dimethyl(oxo)- λ[6]-sulfaneylidene)-2,2-diphenylacetamide (27)

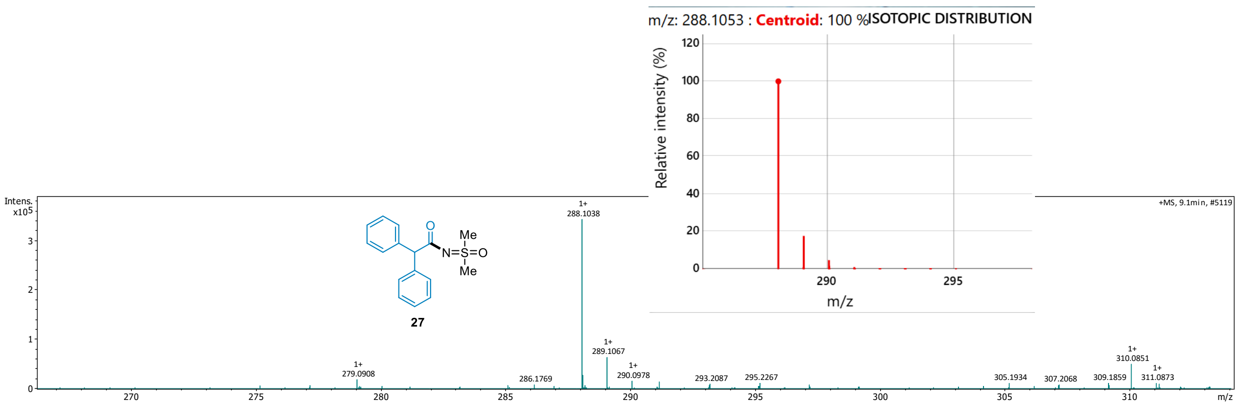

N-(Dimethyl(oxo)- λ[6]-sulfaneylidene)cyclohex-1-ene-1-carboxamide (28)

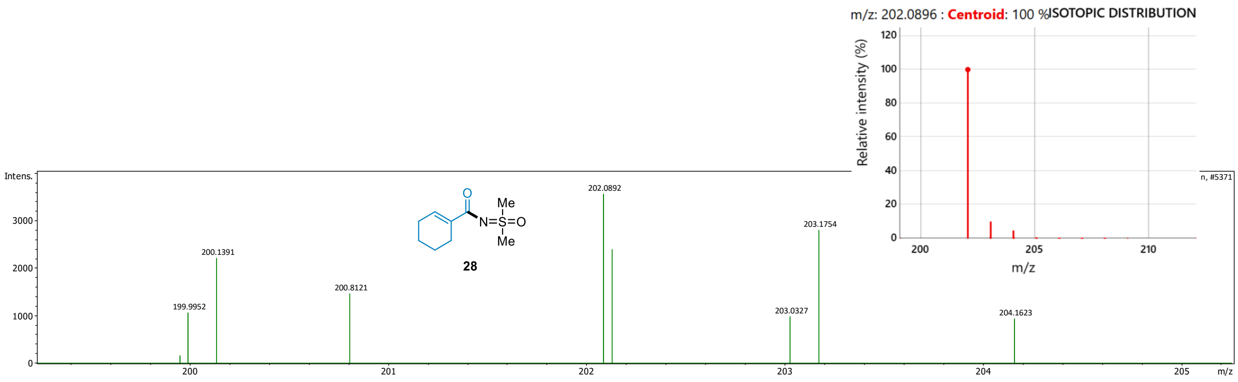

N-(Dimethyl(oxo)- λ[6]-sulfaneylidene)-4-ethynylbenzamide (31)

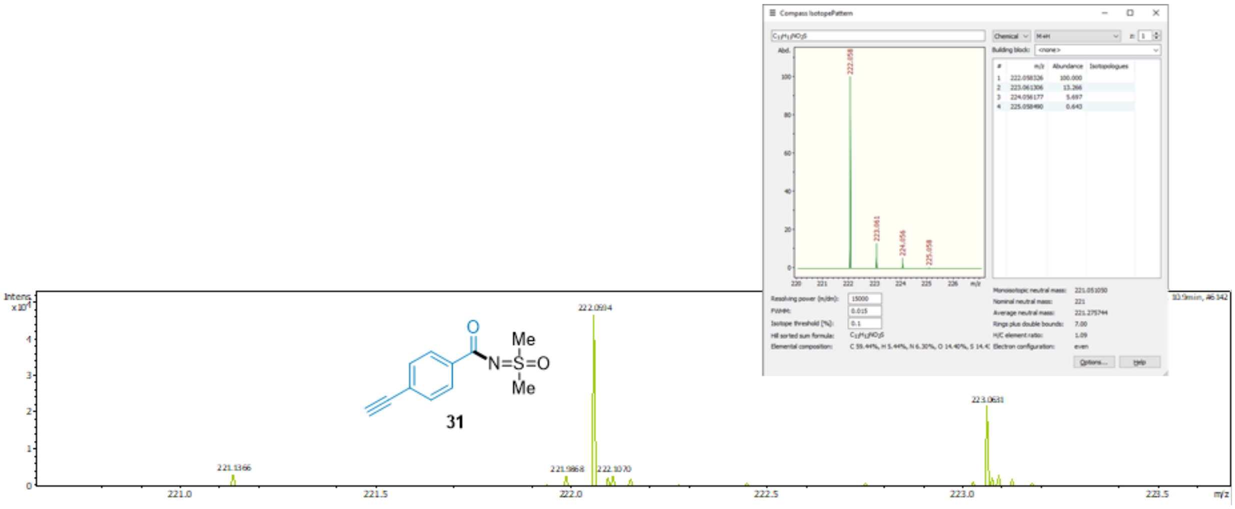

N-(Dimethyl(oxo)- λ^6-sulfaneylidene)-2-phenylpropanamide (32)

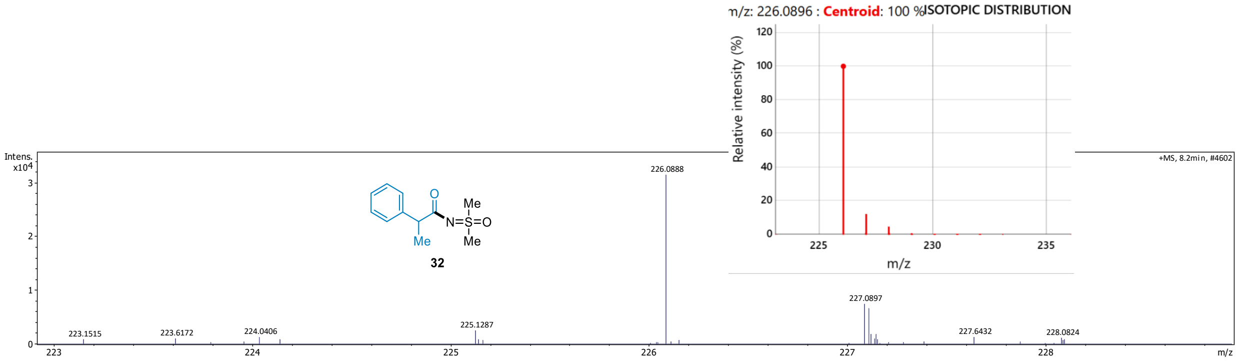

Supplement: Supplementary file 1 [file op6c00160_si_001.pdf]
